# Machine learning reveals distinct gene signature profiles in lesional and nonlesional regions of inflammatory skin diseases

**DOI:** 10.1126/sciadv.abn4776

**Published:** 2022-04-29

**Authors:** Brittany A. Martínez, Sneha Shrotri, Kathryn M. Kingsmore, Prathyusha Bachali, Amrie C. Grammer, Peter E. Lipsky

**Affiliations:** AMPEL BioSolutions, LLC and the RILITE Research Institute, Charlottesville, VA, USA.

## Abstract

Analysis of gene expression from cutaneous lupus erythematosus, psoriasis, atopic dermatitis, and systemic sclerosis using gene set variation analysis (GSVA) revealed that lesional samples from each condition had unique features, but all four diseases displayed common enrichment in multiple inflammatory signatures. These findings were confirmed by both classification and regression tree analysis and machine learning (ML) models. Nonlesional samples from each disease also differed from normal samples and each other by ML. Notably, the features used in classification of nonlesional disease were more distinct than their lesional counterparts, and GSVA confirmed unique features of nonlesional disease. These data show that lesional and nonlesional skin samples from inflammatory skin diseases have unique profiles of gene expression abnormalities, especially in nonlesional skin, and suggest a model in which disease-specific abnormalities in “prelesional” skin may permit environmental stimuli to trigger inflammatory responses leading to both the unique and shared manifestations of each disease.

## Teaser

Gene expression profiling of inflammatory skin diseases reveals common and distinct patterns in lesional and nonlesional regions.

## INTRODUCTION

Autoimmune and inflammatory diseases, such as systemic lupus erythematosus (SLE), can affect many organs, including the skin. Skin manifestations of lupus, known as cutaneous lupus erythematosus (CLE), are common and occur in 70 to 85% of lupus patients (*1*–*3*). Historically, CLE is classified into three subtypes based on clinical and serological features: acute CLE (ACLE), subacute CLE (SCLE), and chronic CLE (CCLE) (*4*). Of these subtypes, discoid lupus erythematosus (DLE) is a chronic form of CLE; DLE is also the most common form of CLE and is characterized by circumscribed regions of inflammation and scarring affecting the skin on the face, head, and below the neck (*4*). The heterogeneity of CLE makes it difficult to determine particular cytokines or inflammatory pathways to target therapeutically, and as a result, no therapies are specifically approved for CLE (*5*). Both an innate immune response, coordinated through Toll-like receptor activation, and multiple adaptive immune responses have been reported in the initiation and propagation of CLE (*2*). Targeting B cells with belimumab (*6*) and type 1 interferon (IFN) with anifrolumab (*7*) shows some benefit in decreasing cutaneous manifestations of SLE. In contrast, other inflammatory skin diseases, such as psoriasis (PSO), have numerous approved therapies (*8*), and dupilumab, an inhibitor of interleukin-4 (IL-4) receptor signaling, is an effective therapy for both atopic dermatitis (AD) and PSO (*9*). This overlap of central, nonredundant pathways between PSO and AD illustrates that diseases with markedly different clinical phenotypes may have similar immunopathogenic underpinnings.

Although independent transcriptomic analyses have provided insight into the molecular landscape of CLE, a complete molecular characterization of the disease is limited by small patient cohorts (*10*–*17*). Previous bulk gene expression studies focused on specific aspects of lupus skin disease, such as the presence of T helper (T_H_) 17 cells (*10*) or specific macrophage populations (*11*), the correlation between inflammatory cell populations and fibroblast marker expression (*12*), cytokine expression (*13*), inflammasome signaling (*14*), or IFN signaling (*15*–*17*). However, there remains a need to examine the interplay of inflammatory cells, nonhematopoietic cells, and pathway perturbations to understand the molecular events in CLE pathogenesis in further detail.

Whereas the dissimilarities in clinical manifestations of CLE and other inflammatory skin diseases have been well documented, the molecular differences between CLE and other inflammatory skin diseases are less completely studied. For instance, keratinocytes, one of the predominant nonhematopoietic cell populations in the skin, have been implicated in PSO pathogenesis (*18*) and shown to be hypersensitive to IFN signaling in CLE (*15*), yet understanding of their role in CLE is limited. Moreover, systemic sclerosis (SSc), another inflammatory skin disease, is characterized by fibrosis and vascular damage due to excessive deposits of extracellular matrix and differentiation of fibroblasts to myofibroblasts (*19*), but little is known about the role of fibrosis in the pathogenesis of CLE. Last, AD is characterized by an allergic reaction owing to a loss in skin barrier function, fibrosis, and T_H_2 cell signaling (*20*), but these functions in CLE have not been explored. Detailed comparison among the molecular signatures of CLE, PSO, AD, and SSc could achieve better understanding of the primary pathogenic mechanisms and provide direction for previously unidentified therapeutic avenues in these conditions.

In this study, we compared the gene expression signatures of four inflammatory skin diseases: CLE, PSO, AD, and SSc. To achieve a read depth sufficient to maintain the in vivo proportions of cellular signals in the biopsies without technical distortion and to capture the majority of molecular pathways, we analyzed bulk RNA. In addition, we used analytic tools to deconvolute transcriptomic data and determine cellular and pathway signals enriched across heterogeneous cohorts of patients from each of the diseases. Using gene set variation analysis (GSVA), we determined that lesional skin of the four skin diseases expressed both shared and unique molecular signatures. Machine learning (ML) demonstrated that both lesional and nonlesional samples of each disease could be classified as distinct from control samples as well as from each other. Notably, nonlesional skin of each disease was more distinct than lesional skin, as there were more common features in ML classification of lesional skin among the four diseases. GSVA confirmed the molecular differences between uninvolved skin of the various conditions. These results suggest a model in which the nonlesional skin of patients with inflammatory skin diseases harbors unique abnormalities that potentially make the skin differentially sensitive to specific environmental stimuli. Inciting stimuli appear to induce responses with many overlapping inflammatory molecular features shared by the diseases. Together, this suggests that therapeutics used in the treatment of one inflammatory skin disease may be useful in the treatment of additional diseases and confirms the utility of gene expression analysis in understanding the immunopathogenesis of clinical and preclinical skin disease.

## RESULTS

### Comprehensive gene expression analysis of DLE reveals similarities and differences with other inflammatory skin diseases

**Fig. 1.**
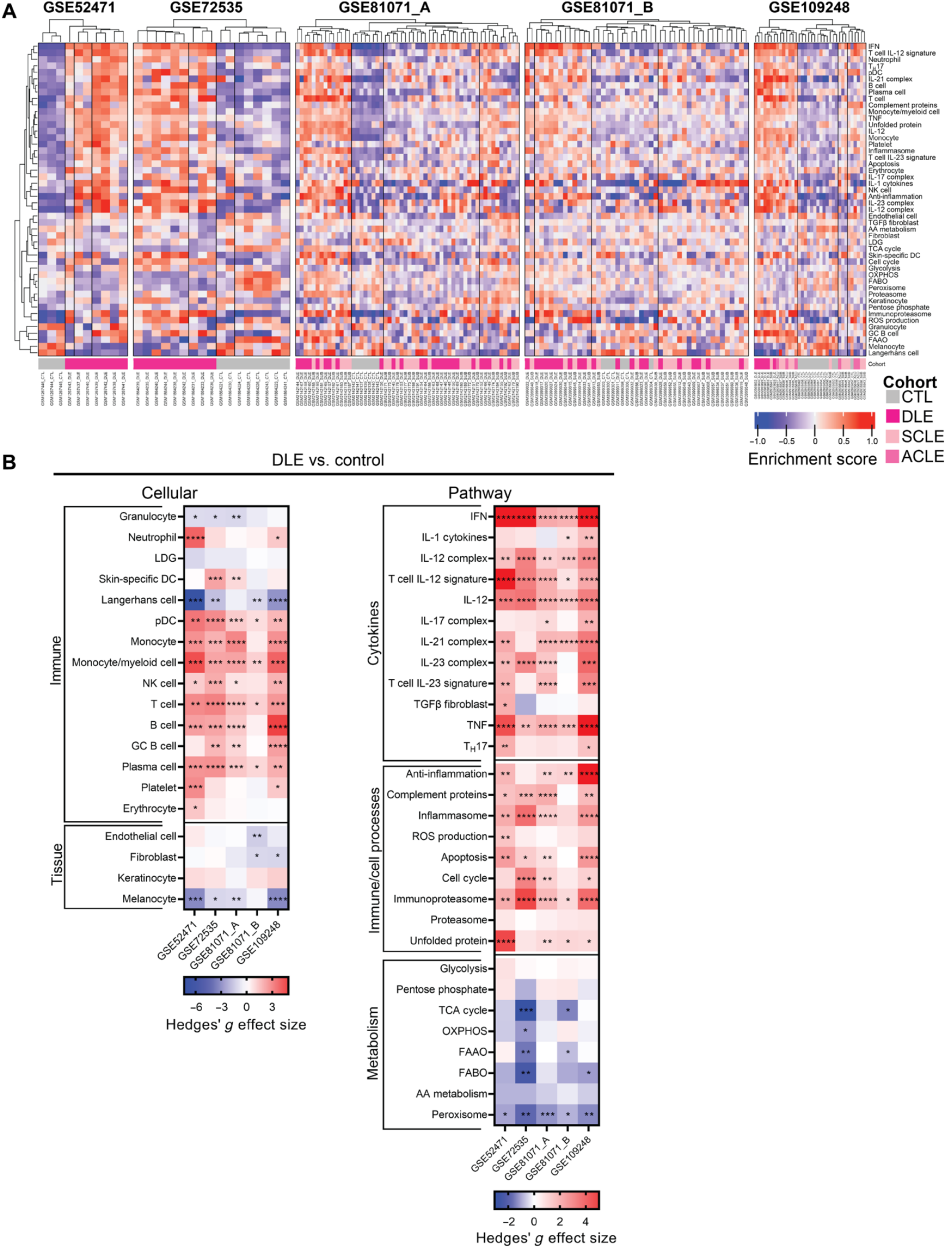
DLE is characterized by enrichment of inflammatory cell and cytokine signatures, including the IFN, IL-12, and TNF signatures. (**A**) Hierarchical clustering (*k* = 4 clusters) of DLE and healthy control samples from five lupus datasets using GSVA enrichment scores of cellular and pathway gene signatures. (**B**) Hedges’ *g* effect sizes of cellular (left) and pathway (right) gene signatures for DLE compared to healthy control samples in five lupus datasets. Heatmap visualization uses red (enriched signature, >0) and blue (decreased signature, <0). Welch’s *t* test: *P < 0.05; **P < 0.01; ***P < 0.001; ****P < 0.0001. AA, amino acid; FAAO, fatty acid α oxidation; FABO, fatty acid β oxidation; LDG, low-density granulocyte; OXPHOS, oxidative phosphorylation; ROS, reactive oxygen species; TCA, tricarboxylic acid.

We carried out a comprehensive transcriptomic analysis of five independent datasets of samples biopsied from both patients with DLE and healthy controls (table S1). DLE was chosen for primary analysis of lupus skin because it is the most frequent subset of CLE (*4*), and it comprised the largest number of available cutaneous lupus samples. To examine cellular and pathway signaling on an individual patient level, we carried out GSVA using a total of 48 informative gene signatures (table S2, A and B). Hierarchical clustering of GSVA enrichment scores demonstrated that DLE was molecularly separable from healthy skin (Fig. 1A). Despite some interpatient heterogeneity in each dataset, signatures for plasmacytoid dendritic cells (pDCs), monocytes, monocyte/myeloid cells, natural killer (NK) cells, T cells, B cells, and plasma cells were consistently enriched in patients with DLE as compared to healthy skin (Fig. 1B, left). Conversely, signatures representative of granulocytes, Langerhans cells, and melanocytes were consistently down-regulated in lupus-affected skin. As previously shown, the IFN gene signature (IGS) was increased in all DLE datasets, as were the IL-12 and tumor necrosis factor (TNF) gene signatures (Fig. 1B, right) (*14*, *17*). There was also enrichment of gene expression of other inflammatory pathways, such as IL-21 and IL-23, complement, and the immunoproteasome. In addition, genes reflective of most metabolic processes were decreased in DLE samples, whereas signatures representing glycolysis and the pentose phosphate pathway remained mostly unchanged.

**Fig. 2.**
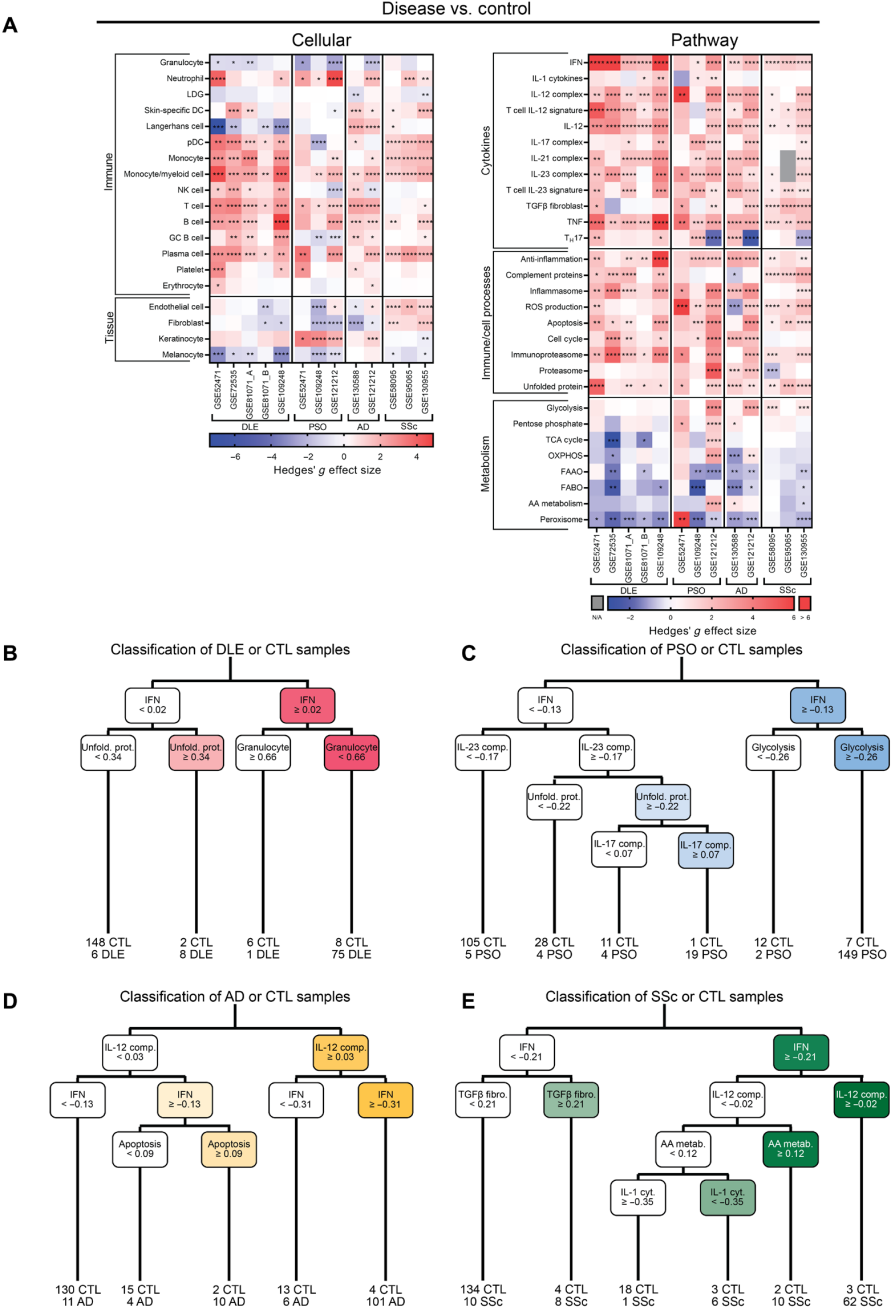
Enrichment of myeloid, lymphoid, IFN, IL-12, IL-23, and TNF signatures is shared among lesional DLE, PSO, AD, and SSc. (**A**) Hedges’ *g* effect sizes of cellular (left) and pathway (right) gene signatures for disease samples compared to their respective control samples in five DLE, three PSO, two AD, and three SSc datasets. Heatmap visualization uses red (enriched signature, >0) and blue (decreased signature, <0). Welch’s *t* test: *P < 0.05; **P < 0.01; ***P < 0.001; ****P < 0.0001. CART analysis for disease or control classification using GSVA enrichment scores in (**B**) DLE, (**C**) PSO, (**D**) AD, and (**E**) SSc. Sample numbers below bottom leaves represent the number of samples of each group classified into that leaf.

To understand the molecular landscape of cutaneous lupus in the context of other inflammatory skin diseases, we examined gene expression data derived from skin biopsies of patients with PSO, AD, or SSc. Overall, there was enrichment of most myeloid and lymphoid-derived cell signatures across all four diseases as compared to control, whereas expression of skin-specific dendritic cells (DCs) differed among the diseases (Fig. 2A, left, and figs. S1 to S4). Monocyte gene signatures were consistently enriched in DLE, AD, and SSc. In contrast, the endothelial cell gene signature was enriched in SSc only. Inflammatory cytokine gene expression was largely enriched in all four diseases, especially pathways involving IFN, IL-12, IL-23, and TNF. DLE and SSc exhibited gene expression enrichment in the complement signature, whereas PSO and AD did not (Fig. 2A, right, and figs. S1 to S4). We noted heterogeneity among datasets of each disease; for example, the IL-17 complex signature was up-regulated in two of three PSO datasets and the Langerhans cell signature was down-regulated in four of five DLE datasets.

Next, we used classification and regression tree (CART) analysis using GSVA enrichment scores of 48 cellular and pathway gene signatures to discern the gene expression variables that best classified the inflammatory skin diseases (Fig. 2, B to E). CART analyses demonstrated that the IGS was the most important feature in disease classification for three of four diseases (DLE, PSO, and SSc). The IGS, unfolded protein, and granulocyte gene signatures were the most important signatures for classifying control or DLE (Fig. 2B), whereas the IGS and glycolysis signatures together classified 83% of PSO samples (Fig. 2C). In contrast, the IL-12 complex, IGS, and apoptosis signatures classified AD or control (Fig. 2D), and the IGS, transforming growth factor β (TGFβ) fibroblast, IL-12 complex, amino acid metabolism, and IL-1 cytokine signatures were the most important features in classifying SSc or control (Fig. 2E). These data show that both common and disease-specific molecular pathway signatures classify the involved skin of the different inflammatory skin diseases.

### ML classification of lesional inflammatory skin samples confirms unique and common molecular pathways

**Fig. 3.**
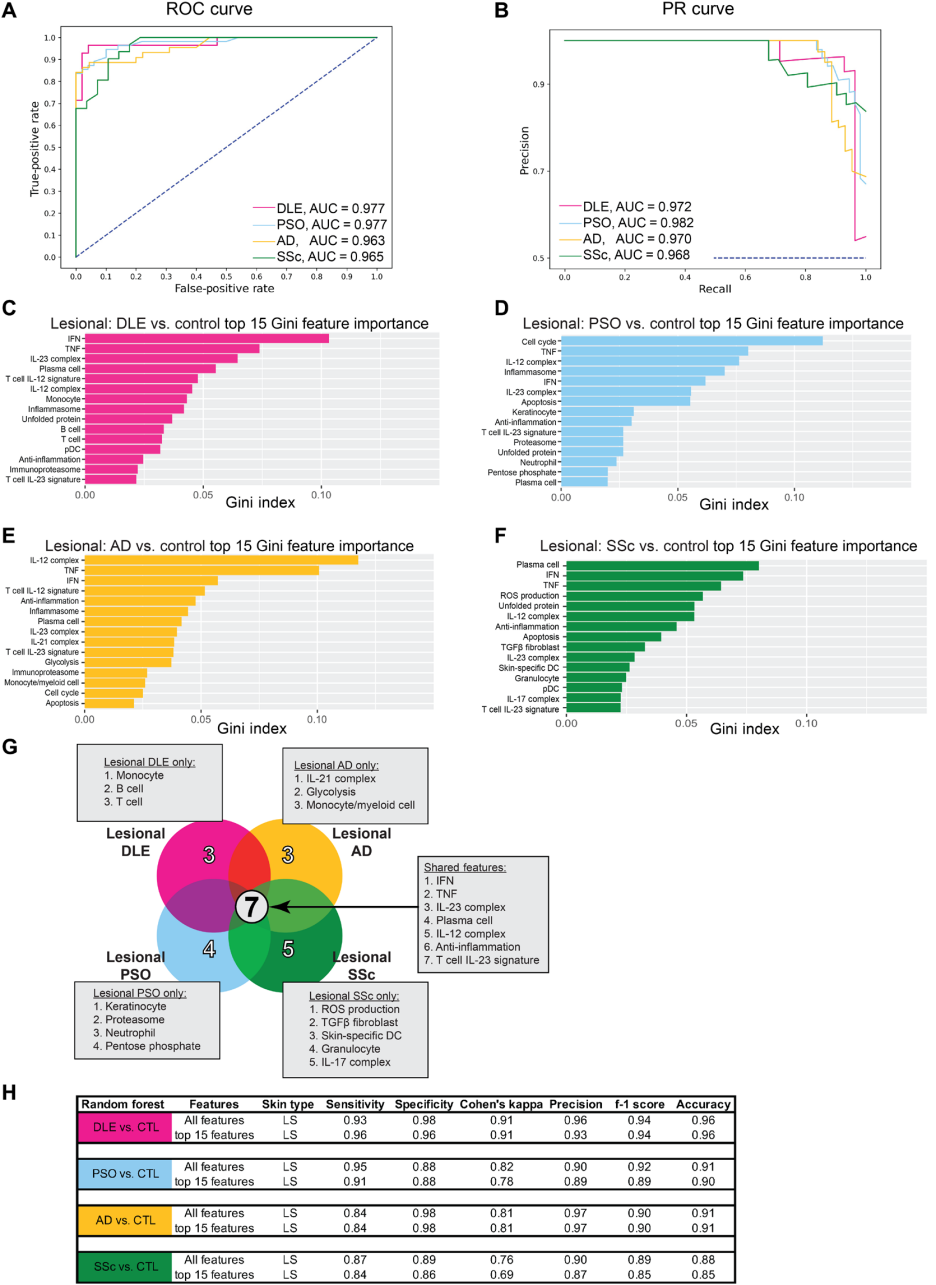
ML effectively classifies lesional skin samples from DLE, PSO, AD, and SSc. (**A**) ROC curve and (**B**) PR curve of lesional DLE, lesional PSO, lesional AD, and lesional SSc samples compared to pooled control samples using all cellular and pathway gene signatures. Top 15 features important in classifying (**C**) lesional DLE, (**D**) lesional PSO, (**E**) lesional AD, and (**F**) lesional SSc from pooled control samples using Gini feature importance. (**G**) Comparison of the top 15 features for classifying each lesional disease compared to control using Gini feature importance. (**H**) Classification metrics to properly separate lesional DLE, lesional PSO, lesional AD, or lesional SSc and control samples using all 48 (top) or the top 15 (bottom) cellular and pathway gene signatures. Refer to table S3 (A and B) for ML details. Collinear features were removed (fig. S6).

To distinguish inflammatory skin diseases more precisely and confirm the major transcriptomic contributors, we used several ML algorithms. First, we examined distinct binary classification of pooled lesional DLE, PSO, AD, and SSc compared to pooled control samples using the ensemble decision tree, random forest (RF), with the 48 cellular and pathway signature GSVA scores as input features. The areas under the receiver operating characteristic (AuROC) curves and precision-recall (AuPR) curves for each binary classification were greater than 0.96 in all cases, indicating excellent performance and appropriate binary classification for each disease compared to control samples (Fig. 3, A and B). For each binary comparison, we determined the top 15 most important features in separating disease from control samples using RF Gini feature importance. To classify DLE from control samples, the IGS, TNF, and IL-23 complex signatures were the most important features (Fig. 3C), whereas to classify PSO from control samples, the cell cycle, TNF, and IL-12 complex signatures were the most important features (Fig. 3D). To classify AD from control samples, the IL-12 complex and TNF signatures as well as the IGS were the most important features (Fig. 3E), and the plasma cell signature, IGS, and TNF signature were the most important features to classify SSc (Fig. 3F). ML analysis elucidated seven features in common among the 15 most important features for classifying each inflammatory skin disease from control, including the IGS and the TNF, IL-23 complex, plasma cell, IL-12 complex, anti-inflammation, and T cell IL-23 signatures (Fig. 3G). It is notable that the 15 most important features performed comparably to the full ensemble of 48 features in binary classification of inflammatory skin disease samples (Fig. 3H). In addition, a number of other ML algorithms were similarly effective at binary classification of these samples (fig. S5).

**Fig. 4.**
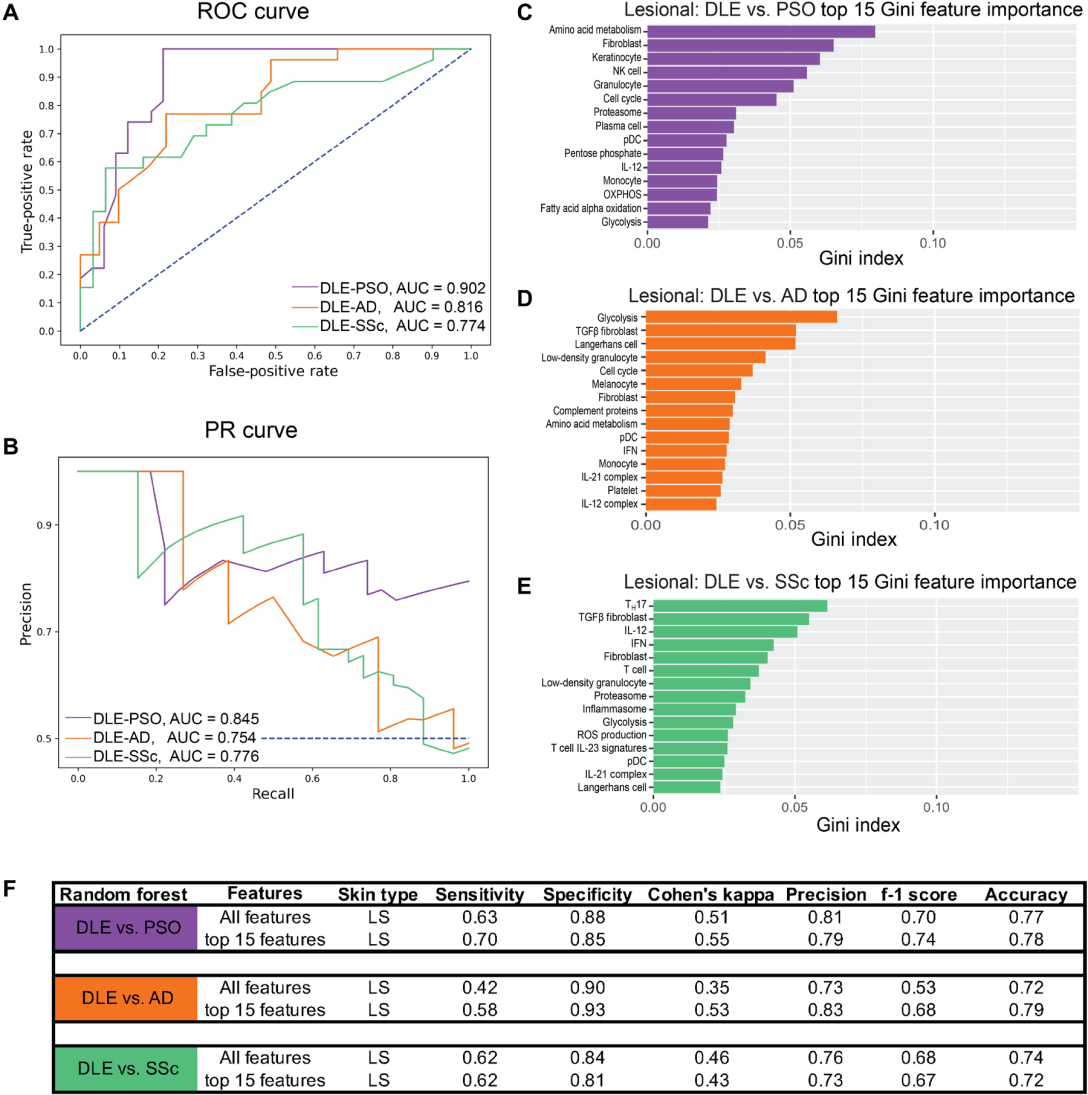
ML classification of DLE versus PSO, AD, and SSc confirms distinct disease-specific gene signatures. (**A**) ROC curve and (**B**) PR curve of lesional DLE samples compared to lesional PSO (purple) samples and lesional DLE samples compared to lesional AD samples (orange) using all cellular and pathway gene signatures. Top 15 features important in classifying (**C**) lesional DLE and lesional PSO, (**D**) lesional DLE and lesional AD, and (**E**) lesional DLE and lesional SSc using Gini feature importance. (**F**) Classification metrics to properly separate lesional DLE samples and lesional PSO or lesional AD samples using all 48 (top) or the top 15 (bottom) cellular and pathway gene signatures. Refer to table S3 (A and B) for ML details. Collinear features were removed (fig. S9).

Next, we directly compared gene expression signatures of DLE samples with those of other inflammatory skin diseases. Distinctions in cellular and pathway signature enrichment among DLE and PSO samples were observed using hierarchical clustering of GSVA scores in two datasets with samples from these inflammatory skin conditions (fig. S7A). Notably, transcriptomic signatures that differed between DLE and PSO included the IGS as well as the Langerhans cell and T cell IL-12 gene signatures (fig. S7B). Since we lacked datasets in which DLE samples were analyzed concurrently with AD and SSc, we used ML algorithms to classify samples from these conditions using the 48 GSVA enrichment scores as features. Performance characteristics and AuROC and AuPR curves demonstrated less effective classification of DLE from other diseases than the classification of each disease versus control (Fig. 4, A and B). In the binary classification of DLE and PSO samples, the most important features included the amino acid metabolism, fibroblast, and keratinocyte gene signatures (Fig. 4C), whereas classification of DLE and AD samples involved the glycolysis, TGFβ fibroblast, and Langerhans cell gene signatures (Fig. 4D). The T_H_17, TGFβ fibroblast, and IL-12 signatures were most important in separating DLE and SSc samples (Fig. 4E). Classification using only the top 15 most important features was as effective as when all 48 features were used (Fig. 4F). Last, other ML classifiers performed similarly to RF (fig. S8).

### Transcriptomic profiles of nonlesional skin samples distinguish inflammatory skin diseases from each other

**Fig. 5.**
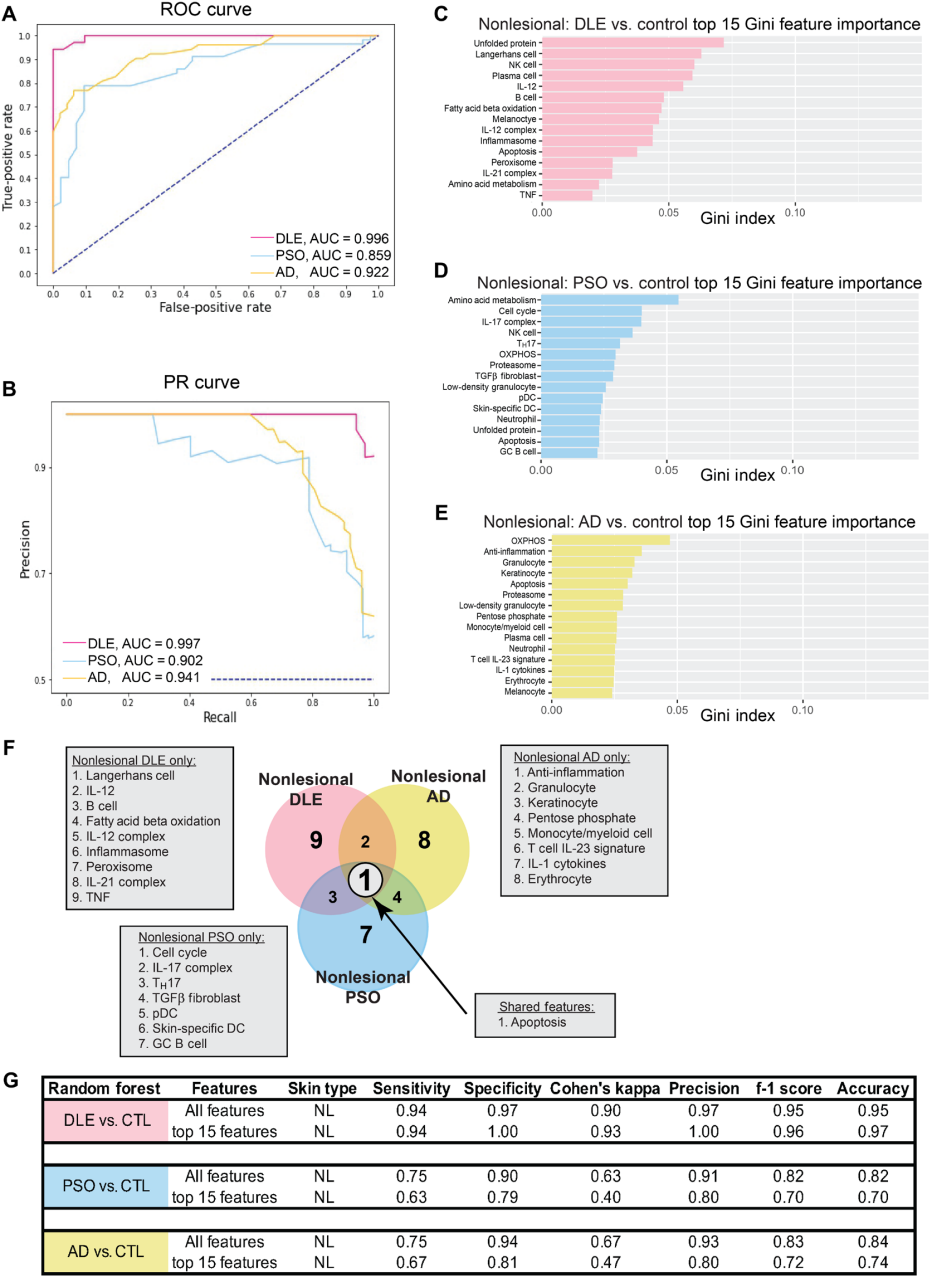
ML classification reveals that nonlesional skin of DLE, PSO, and AD is distinct from control skin. (**A**) ROC curve and (**B**) PR curve of nonlesional DLE, nonlesional PSO, and nonlesional AD samples compared to pooled control samples using all cellular and pathway gene signatures. The top 15 features important in classifying (**C**) nonlesional DLE, (**D**) nonlesional PSO, and (**E**) nonlesional AD and control samples using Gini feature importance. (**F**) Comparison of the top 15 features for classifying each nonlesional disease compared to control using Gini feature importance. (**G**) Classification metrics to properly separate nonlesional DLE and control samples, nonlesional PSO, and control samples, as well as nonlesional AD and control samples using all 48 (top) or the top 15 (bottom) cellular and pathway gene signatures. Refer to table S3 (A and B) for ML details. Collinear features were removed (fig. S12).

Although the molecular characteristics of lesional skin in each independent disease have been well studied, less is known about the transcriptomic profiles of uninvolved skin. To determine whether there are underlying immunological abnormalities contributing to disease, we examined gene expression profiles in nonlesional skin samples from patients with DLE, PSO, and AD to assess the extent to which they differed from either control or lesional skin; nonlesional SSc data were not available for analysis. Analysis of GSVA enrichment scores demonstrated that nonlesional samples were transcriptionally different from lesional samples (fig. S10). The up-regulation of inflammatory pathways including IFN, IL-12, IL-23, and TNF gene signatures in lesional versus nonlesional skin mirrored the up-regulation of these same pathways observed in comparison of lesional versus control skin in DLE, PSO, and AD (Fig. 2A). We next carried out ML classification using GSVA scores to determine whether nonlesional samples were different than control. To overcome class imbalance issues, we used undersampling and synthetic minority oversampling technique (SMOTE) to balance the number of samples in each class to optimize each binary classification. ML was able to classify nonlesional disease and healthy control samples reliably (Fig. 5, A and B). Classification of nonlesional DLE and control showed that the unfolded protein, Langerhans cell, and NK cell signatures were the most important features (Fig. 5C), whereas the amino acid metabolism, cell cycle, and IL-17 complex signatures were the most important features to classify nonlesional PSO and control (Fig. 5D). By contrast, the oxidative phosphorylation, anti-inflammation, and granulocyte signatures were the most important features in classifying nonlesional AD from control (Fig. 5E). Notably, comparison of the top 15 features of each binary classification showed that there was minimal overlap of important features among nonlesional skin diseases, with only one feature, apoptosis, shared among nonlesional DLE, PSO, and AD (Fig. 5F). The binary classifications performed accurately when only the top 15 features of each binary classification were used (Fig. 5G). Moreover, other ML classifiers performed similarly to RF (fig. S11). These data indicate that nonlesional skin in each of the three diseases evaluated is uniquely different from control skin.

**Fig. 6.**
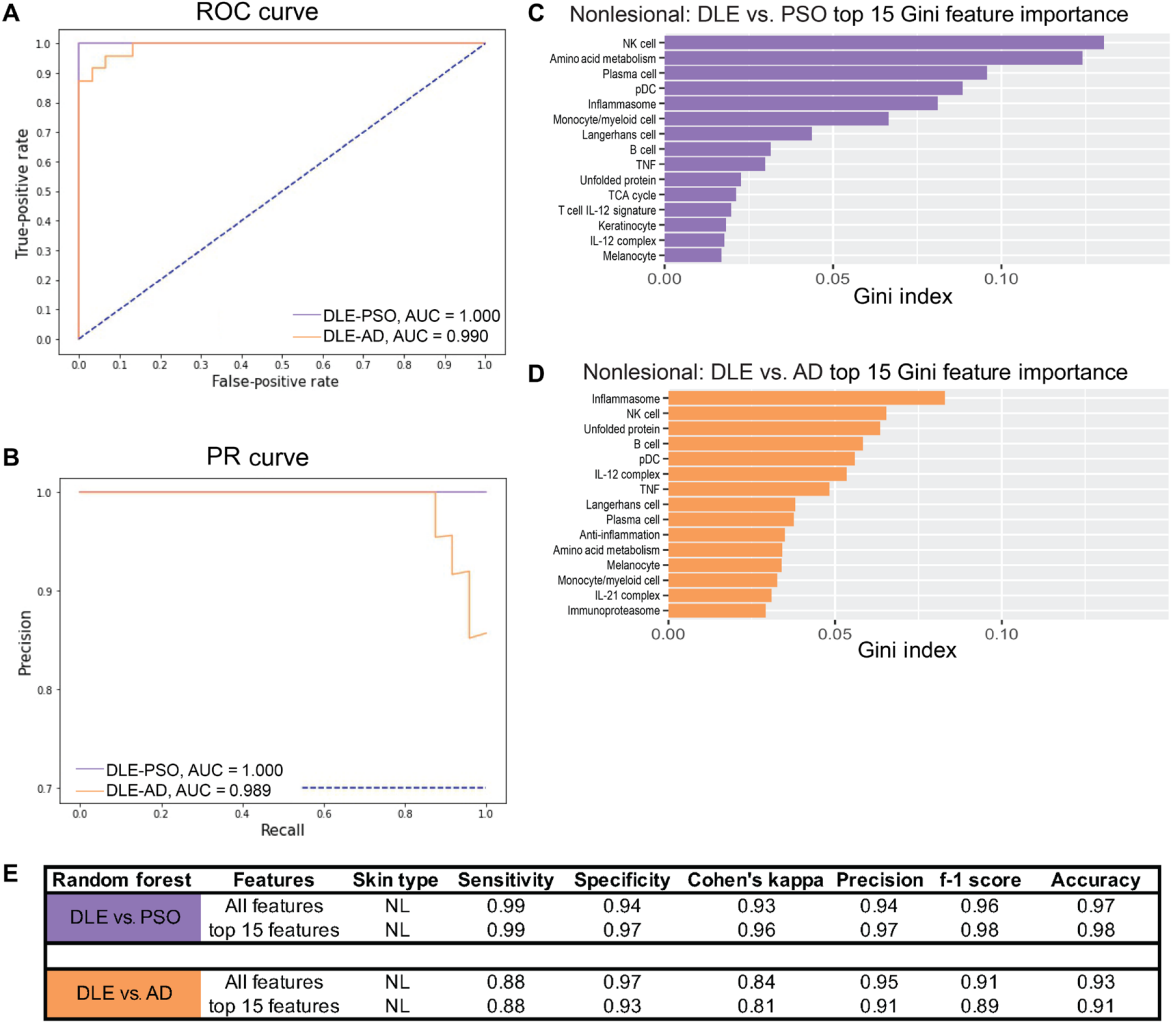
Nonlesional DLE is distinct from PSO and AD. (**A**) ROC curve and (**B**) PR curve of nonlesional DLE samples compared to nonlesional PSO (purple) samples and nonlesional DLE samples compared to nonlesional AD samples (orange) using all cellular and pathway gene signatures. Top 15 features important in classifying (**C**) nonlesional DLE and nonlesional PSO and (**D**) nonlesional DLE and nonlesional AD using Gini feature importance. (**E**) Classification metrics to properly separate nonlesional DLE samples and nonlesional PSO or nonlesional AD samples using all 48 (top) or the top 15 (bottom) cellular and pathway gene signatures. Refer to table S3 (A and B) for ML details. Collinear features were removed (fig. S15).

Given that nonlesional skin of each disease was distinct from control skin and appeared to be distinct from other diseases, we next compared our binary classification of nonlesional DLE compared to nonlesional PSO or nonlesional AD using balance strategies described above (Fig. 6, A and B). We found that nonlesional DLE and nonlesional PSO are easily separable, with the NK cell, amino acid metabolism, and plasma cell signatures being the top features used in classification (Fig. 6C). In the binary classification of nonlesional DLE compared to nonlesional AD, the top features included the inflammasome, NK cell, and unfolded protein signatures (Fig. 6D). Comparable classification was observed with the top 15 features only (Fig. 6E), and other ML classifiers gave similar results (fig. S13). Last, binary classification of nonlesional PSO and nonlesional AD exhibited less effective classifier performance, but classification based on signatures including amino acid metabolism, IL-23 complex, and cell cycle was achieved (fig. S14). These results indicate that transcriptomic profiles of nonlesional skin are sufficiently unique to distinguish different inflammatory skin diseases. Moreover, the differences among nonlesional skin of the three diseases appear to be greater than the differences among lesional skin of the same three diseases.

**Fig. 7.**
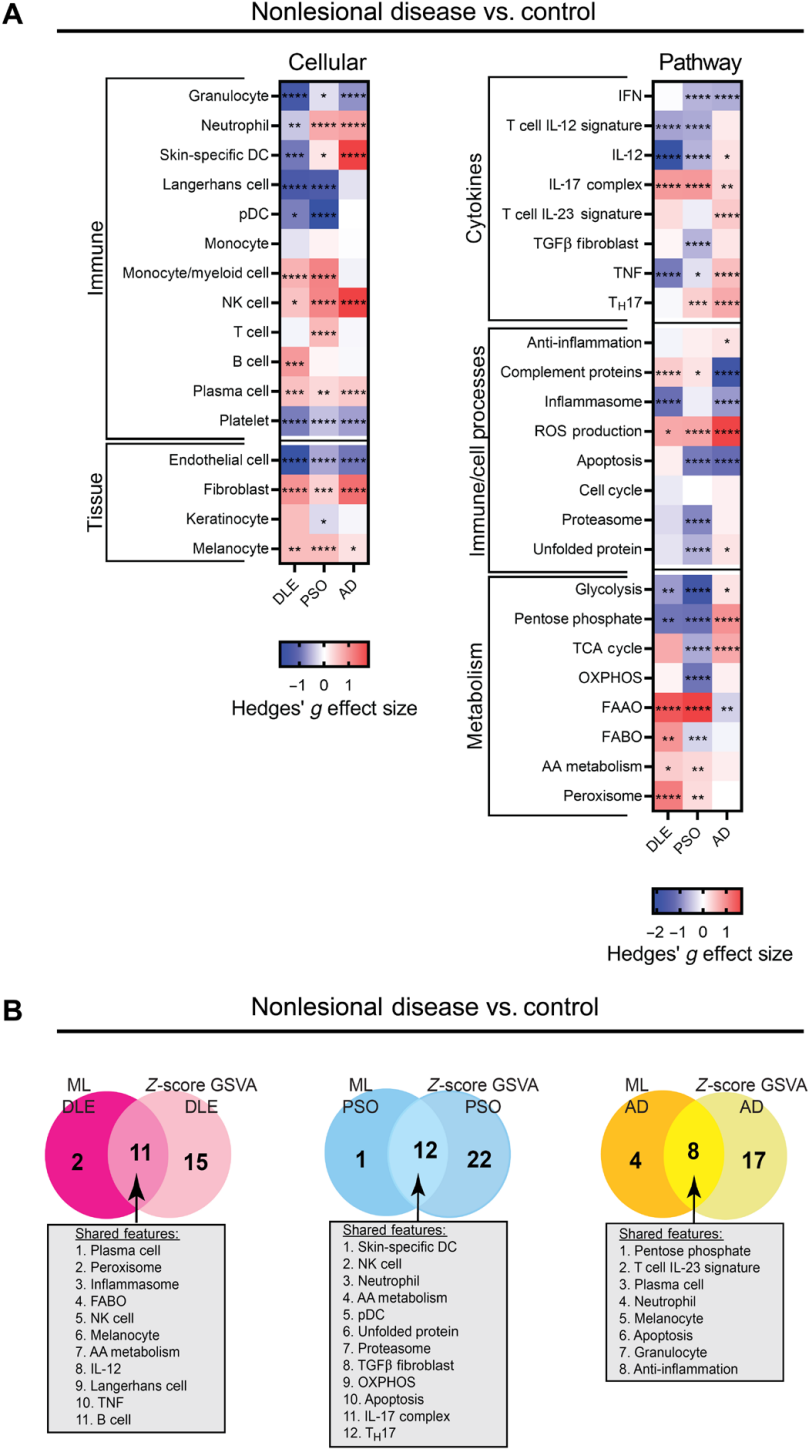
Nonlesional skin is characterized by up-regulation of unique cellular and pathway signatures. (**A**) Hedges’ *g* effect sizes of cellular (left) and pathway (right) gene signatures for pooled nonlesional disease samples compared to pooled control samples DLE, PSO, and AD datasets. Heatmap visualization uses red (enriched signature, >0) and blue (decreased signature, <0). Welch’s *t* test: *P < 0.05; **P < 0.01; ***P < 0.001; ****P < 0.0001. (**B**) Comparison of the most important features determined by ML that are also statistically significant by *z*-score GSVA of nonlesional skin versus controls for nonlesional DLE (left), nonlesional PSO (middle), and nonlesional AD (right). Forty signatures were used in the nonlesional *z*-score GSVA; only these features were used in the comparison to nonlesional ML.

ML used specific gene signatures for the classification of nonlesional skin samples as compared to control. To probe the disease-specific differences in nonlesional skin in greater detail, we carried out an additional analysis using GSVA. For this analysis, we pooled the control samples and nonlesional samples from all datasets and used *z*-score normalization to scale expression data from samples obtained from different datasets. We found that nonlesional DLE samples compared to control samples show up-regulation of B cells, melanocytes, and complement protein gene signatures (Fig. 7A and fig. S16). Nonlesional PSO samples showed up-regulation of T cell and T_H_17 gene signatures, whereas nonlesional AD samples compared to control samples showed up-regulation of skin-specific DC, IL-12, and anti-inflammation gene signatures as compared to control samples (Fig. 7A and figs. S17 and S18).

To confirm these GSVA results, we also used a mean of *z*-score calculation for enrichment of signatures and found similar results (figs. S19 to S21 and table S4). Notably, comparison of significantly enriched signatures determined by the *z*-score GSVA approach and the topmost important features for ML classification of nonlesional skin demonstrated considerable overlap (Fig. 7B). Of the 40 features used in both approaches, the majority of discriminatory features were similar. For example, in nonlesional DLE, there were 11 shared features between ML and *z*-score GSVA methods including the plasma cell, TNF, Langerhans cell, and B cell signatures. In nonlesional PSO, there were 12 shared features between the two methods including the skin-specific DC, IL-17 complex, and T_H_17 signatures. In nonlesional AD, there were eight shared features between the two methods including neutrophil, plasma cell, and anti-inflammation signatures.

### ML successfully classifies different subtypes of CLE

Because the ML approach was able to determine specific signatures that separated related diseases, we sought to determine whether this same approach could distinguish subtypes of CLE based on their gene expression profiles. By hierarchical clustering, we observed that patients among the three CLE subtypes (DLE, SCLE, and ACLE) did not separate cleanly from control samples (fig. S22A). To interrogate molecular differences in CLE subtypes in greater detail, we examined individual gene signature enrichment among DLE and SCLE samples as compared to control, as they comprised sufficient sample numbers for further analysis. We previously observed a robust up-regulation of immune cell gene signatures, including the pDC, monocyte, T cell, and B cell signatures, by GSVA comparison of DLE and control samples (refer to Fig. 1B). The monocyte, T cell, and B cell signatures were up-regulated and the Langerhans cell signature was down-regulated in patients with SCLE as compared to healthy skin in a similar pattern to DLE (fig. S22B). In contrast, comparison of DLE and SCLE revealed minimal signatures with significant differences between the two CLE subtypes (fig. S22C). By hierarchical clustering, samples from patients with SCLE did not form distinct clusters but rather were interspersed with DLE, suggesting that subtypes of lupus are not molecularly separable (fig. S23A). However, when we applied ML to determine whether DLE could be distinguished from SCLE (fig. S23B), classification with good performance characteristics was achieved (fig. S23, C and D). The top 15 features important in distinguishing DLE from SCLE included plasma cell, unfolded protein, and TNF signatures (fig. S23E). It is notable that the pattern of gene enrichment signatures is similar in DLE and SCLE, but the magnitude of enrichment of gene signatures was greater in DLE. These quantitative differences are sufficient for ML to classify the two subtypes of CLE effectively (fig. S23F).

### Cytokine-stimulated keratinocyte signatures and T cell signatures differ among inflammatory skin diseases

Keratinocyte and T cell signatures are often up-regulated in inflammatory skin diseases. Because the ML analyses to this point have focused on gene signatures previously implicated in lupus (*17*, *21*, *22*), with less emphasis on those implicated in other inflammatory skin diseases (*23*, *24*), we also examined enrichment of previously published PSO- or AD-specific gene signatures in the four lesional inflammatory skin diseases as compared to healthy controls. To accomplish this, we first evaluated gene sets derived from keratinocytes stimulated with various cytokines (table S2C). We found, in all diseases, that many of the keratinocyte gene signatures were highly enriched (fig. S24). DLE was highly enriched for the IFN-stimulated keratinocyte gene signatures, whereas PSO was highly enriched in IL-1– and IL-17–stimulated keratinocyte gene signatures. Keratinocyte gene signatures were up-regulated equally in AD, including IFN- and IL-17–stimulated keratinocyte gene signatures. SSc showed more modest increases to the keratinocyte gene signatures, with IL-1–, IL-17–, and TNF-stimulated keratinocyte signatures decreased in some datasets. These data indicate a possible role for cytokine-stimulated keratinocytes in the pathogenesis of each disease, with a less prominent effect in SSc. Collinearity analysis revealed that the keratinocyte signatures were highly correlated and, therefore, not appropriate for use in ML (fig. S25). Second, we examined the role of more nuanced T cell populations. For example, T_H_17 cells and IL-17 are targets of successful therapeutic intervention in PSO (*25*, *26*); in addition, the T_H_2 T cell subset is implicated in AD pathogenesis (*20*). GSVA analysis of the T cell gene signatures demonstrated heterogeneity across the PSO, AD, and SSc datasets. T follicular helper (T_FH_) cell, T_H_1 cell, and T_H_17 cell signatures were enriched in most DLE datasets; PSO had variable enrichment of T_H_1 cell, T_H_2 cell, and T_H_17 cell signatures, as did AD, while SSc exhibited less robust T cell enrichment (fig. S26).

## DISCUSSION

Here, we used a comprehensive analysis of gene expression profiles to characterize the molecular features of four inflammatory skin diseases. Although considerable inter- and intra-dataset heterogeneity was observed, we documented molecular gene signatures that define both lesional and nonlesional skin of the various conditions. Notable among the findings were the shared and unique features of lesional skin among the four diseases and the unique features of nonlesional uninvolved skin. Together, this analysis demonstrates the informative power of transcriptomics to determine pathological characteristics of clinically abnormal (lesional) skin as well as unique features of clinically uninvolved (nonlesional) skin of each inflammatory skin disease.

Our analyses involved multiple bioinformatic and statistical approaches that allowed us to understand the molecular pathways underlying the preclinical and clinical stages of inflammatory skin diseases. First, we assessed numerous datasets for each disease so that we could capture the transcriptional landscape of each condition and overcome the heterogeneity among patients and datasets. Second, each dataset was independently evaluated by GSVA using informative gene signatures we previously used in the analysis of lupus (*17*, *21*, *22*, *27*), gene signatures derived from interrogation of other inflammatory skin diseases (*23*, *24*), and additional signatures we generated because of their relevance to skin pathogenesis. This analysis allowed us to observe unique patterns in the enrichment of inflammatory pathway signatures among and between the diseases and document that the diseases were molecularly separable. We used ML models, including CART and RF, to determine that effective classification between disease and control or between diseases was achievable and to identify the most important features labeling the conditions. The ML models not only permitted the effective classification of samples but also allowed dimensionality reduction, scaling the original 48 input gene signatures down to 15 features most important in each classification.

Despite previously noted heterogeneity (*28*, *29*), our analysis revealed that the molecular landscape of DLE was more homogeneous across datasets comprising patients from different centers and thus was sufficiently similar to permit accurate classification. Similarly, datasets including patients with SSc demonstrated consistent gene expression patterns. In contrast, we and others found datasets comprising patients with PSO and AD to be more molecularly heterogeneous (*30*). Nevertheless, we identified definitional transcriptional elements for each of the various conditions that included both shared and specific molecular perturbations. Comparison of the lesional DLE, PSO, AD, and SSc transcriptomes using GSVA demonstrated that these four inflammatory skin diseases have numerous inflammatory pathways in common. IFN, TNF, IL-12 complex, IL-23 complex, T cell IL-23, anti-inflammation, and unfolded protein gene signatures were commonly up-regulated among lesional biopsies from the four inflammatory diseases. CART analysis, which was used as an initial algorithm to detect important discriminators within the data, demonstrated that the IFN and IL-12 complex gene signatures were the two most important features in distinguishing lesional DLE, PSO, AD, and SSc from control samples. Moreover, ML algorithms documented that, of the 15 features necessary for accurate classification of each disease from control, 7 features are common among all four diseases, including the IGS, IL-12 complex, IL-23 complex, TNF, plasma cell, T cell IL-23, and anti-inflammation gene signatures. Together, there were six shared and up-regulated features between the GSVA and ML methods, suggesting that, despite different genetic predispositions and disease manifestations, lesional DLE, PSO, AD, and SSc have a common inflammatory microenvironment that differentiates them from control skin. This was further supported by the overlapping enrichment of numerous signatures among at least two of the four diseases. For example, GSVA demonstrated that the neutrophil signature was up-regulated in the majority of PSO and SSc patients, whereas the pDC, monocyte, monocyte/myeloid cell, and B cell signatures were increased in the majority of DLE and SSc patients; the T cell, IL-21 complex, inflammasome, and cell cycle signatures were increased in all diseases, except SSc.

Despite the similarities among the diseases, however, we detected unique characteristics of each inflammatory skin disease. We observed clear molecular distinctions between lesional samples from patients with DLE, PSO, AD, and SSc. The GSVA analysis revealed that the NK cell signature was only up-regulated in lesional DLE compared to control samples, whereas the IL-1 cytokine signature was uniquely up-regulated in lesional PSO compared to controls. Notably, however, neither signature proved to be of particular importance in ML classification of either disease from controls. Similarly, the proteasome and Langerhans cell signatures were uniquely enriched in AD compared to control samples, and the endothelial cell and fibroblast signatures were uniquely enriched in lesional SSc compared to controls, although these signatures were not of particular importance in ML classification of either disease. Despite this complexity, ML was able to delineate the most important features for classification of each condition from normal. For example, although increased in some patients from all diseases, the monocyte, T cell, and B cell signatures were more important in the classification of DLE. Moreover, the keratinocyte and neutrophil signatures were most important in classifying PSO, and not the other diseases, a finding that is consistent with the role of keratinocyte proliferation and neutrophil infiltration in PSO (*18*, *31*, *32*). In addition, the IL-21 complex signature was up-regulated in all diseases except SSc, but was unique to ML classification of AD, consistent with the role of IL-21 in allergic skin diseases (*33*, *34*). Last, the TGFβ fibroblast signature was important in classification of SSc, which aligns with the central role of fibrosis in this disease (*23*, *35*). Furthermore, ML demonstrated that the pDC, fibroblast, and glycolysis signatures are important in classifying lesional DLE from the other lesional diseases, illustrating that ML can effectively classify diseases through identification of molecular changes among samples. These findings strongly imply that there are unique molecular features in lesional biopsies of inflammatory skin disease, along with a panoply of shared features.

Although there are numerous reports of gene expression abnormalities in lesional skin, less is known about the architecture of clinically uninvolved nonlesional skin as compared to healthy skin. Examination of nonlesional skin in DLE, PSO, and AD provided previously unknown insights into the molecular processes operating in uninvolved skin and suggested a unique preclinical set of abnormalities in each condition. Application of both ML- and *z*-score–based approaches as orthogonal analytic tools to assess the differences between nonlesional and normal skin revealed unique patterns of abnormalities in each inflammatory skin condition. Notably, only the apoptosis signature was among the top 15 features used by ML to classify nonlesional DLE, PSO, and AD versus pooled controls. This suggests that dysregulated apoptosis may be a key feature in the initiation of each of these three diseases; apoptosis is cited in the pathogenesis of skin diseases including CLE, PSO, and AD, and enhanced apoptosis is a well-recognized feature of SLE (*36*–*39*). In general, unique molecular features characterize each condition, such as the IL-21 pathway in CLE and IL-17 in PSO. Genetic polymorphisms may contribute to the abnormalities noted in nonlesional skin, as, for example, susceptibility to lupus is in part associated with polymorphisms in the IL-21 axis (*40*) and polymorphisms in IL-17 are correlated with PSO treatment response (*41*).

Of note, unlike lesional disease, we did not observe a prominent role for the IGS in nonlesional skin from DLE, PSO, or AD. This contrasts with some previous studies suggesting that nonlesional skin from patients with SLE or DLE is influenced by type 1 IFN (*42*–*44*). However, this contention was based largely on single-cell RNA sequencing (RNA-seq) analysis of nonlesional keratinocytes and their expression of the IGS (*42*, *43*), whereas our studies have evaluated expression of the IGS by deconvolution of bulk tissue gene expression. Our data revealed increased IGS in a few DLE samples, which may align with the increase of IFN action in only select cell clusters from single-cell RNA-seq analysis. Together, this suggests that IFN is not a dominant factor of nonlesional disease in either CLE or PSO and may instead reflect the concurrent exposure to ultraviolet (UV) light or the presence of specific autoantibodies, both of which are associated with up-regulation of the IGS (*45*–*47*).

**Fig. 8.**
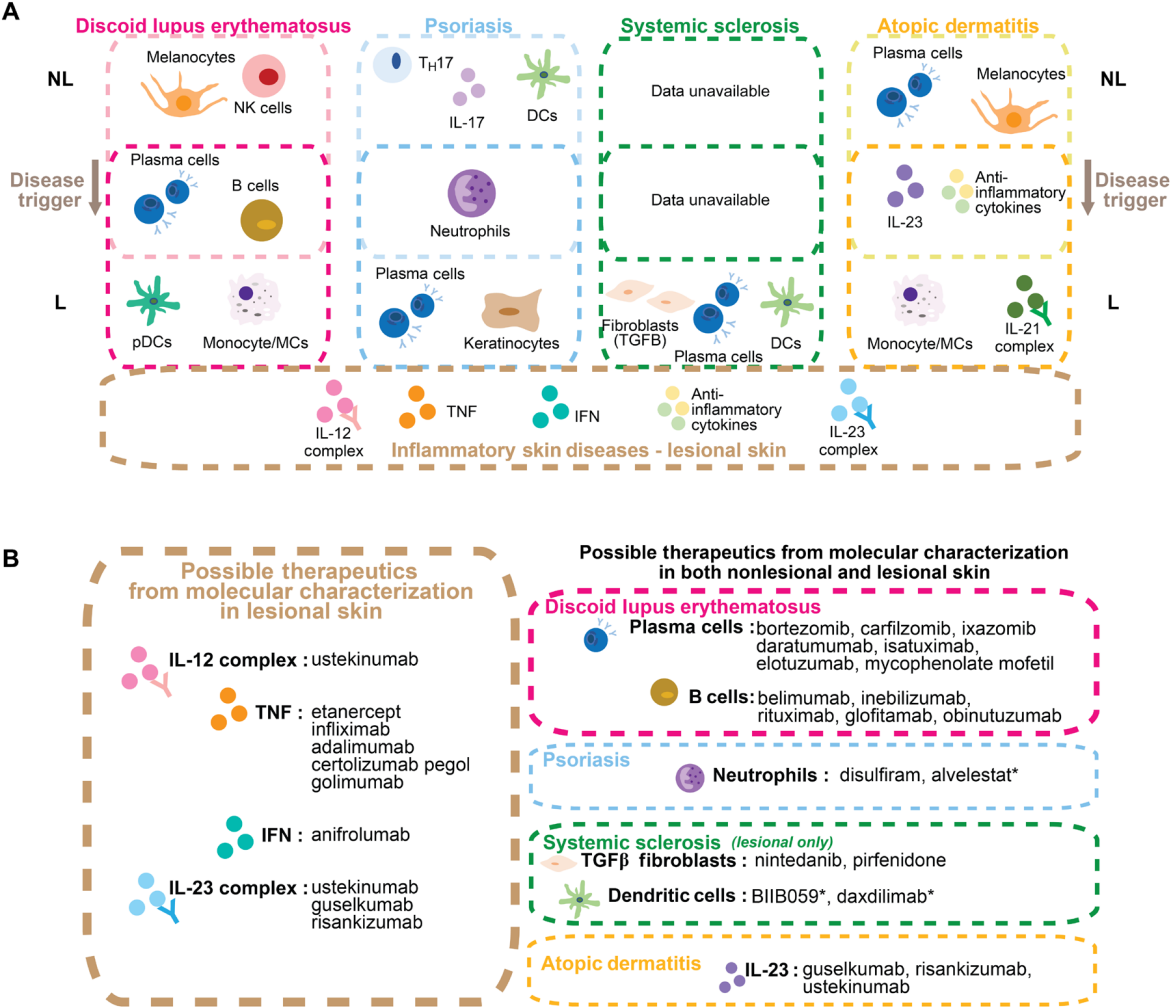
Nonlesional skin from patients with inflammatory skin diseases manifests a specific set of preclinical, molecular abnormalities that predispose the development of both shared and unique clinical features in lesional DLE, PSO, AD, and SSc after encountering an environmental trigger. (**A**) Summary graphic detailing features determined by ML and up-regulated in nonlesional skin or lesional skin of DLE, PSO, AD, and SSc versus control as determined by GSVA. Some features are up-regulated in both nonlesional and lesional skin. The bottom box shows important ML features up-regulated by GSVA in lesional skin and shared among all four inflammatory skin diseases. Refer to table S4 for details about comparison between GSVA and *z*-score methods. (**B**) Summary of possible therapies of lesional skin diseases analyzed (left) and possible therapies for both lesional and nonlesional regions of each disease (right) based on molecular characterization. * delineates drugs in development.

Together, the data suggest a model in which patients with inflammatory skin disease manifest a specific set of preclinical molecular abnormalities that could predispose a patient to the development of typical clinical features, perhaps after encountering an environmental trigger (such as UV light, bacterial products, or allergens). Upon development of cutaneous inflammation, common molecular features are up-regulated, although the lesional disease concurrently maintains a unique gene expression profile (Fig. 8A). This model is consistent with reports that nonlesional skin of patients with inflammatory skin disease is a pre-inflammatory or “primed” state, and that some of the same molecular processes may contribute to maintaining both the pre-inflammatory and inflammatory components of the skin conditions (*30*). Here, we see not only an overlap of gene signatures up-regulated in lesional skin between skin conditions but also some overlap between nonlesional and lesional skin within each inflammatory skin disease.

Previous reports were not able to separate molecular features of DLE from those of SCLE despite the marked differences in clinical phenotype (*48*). Both DLE and SCLE are characterized by interface dermatitis, but the differences in clinical manifestations suggest different molecular underpinnings. By GSVA, gene expression profiles of these two entities were similar to each other. GSVA analysis showed that the same gene signatures were significantly enriched in each subtype compared to control. However, the effect size in significantly enriched gene signatures was greater in DLE compared to SCLE. The quantitative differences were sufficient for ML to classify DLE from SCLE by using predominately the plasma cell, neutrophil, pDC, melanocyte, and germinal center (GC) B cell features as well as the TNF, IL-12, and IL-1 cytokine inflammatory features to classify CLE subtypes. This is supported, in part, by previous studies of skin biopsies of SCLE patients demonstrating the presence of TNF by immunofluorescence staining in lesional but not nonlesional samples (*49*).

The results of this analysis lend insight into future treatment strategies for DLE, PSO, AD, and SSc based on the observed common and distinct molecular characteristics (Fig. 8B). For example, IL-17 is a well-known target for PSO treatment (*50*) and has been explored in therapy for lupus (*51*, *52*) and AD (*53*); however, we did not observe consistent up-regulation of the IL-17 complex signature among the lesional manifestations of DLE, AD, and SSc, suggesting that IL-17–neutralizing therapy may be best suited for lesional PSO alone. However, we observed up-regulation of IL-17 complex and T_H_17 gene signatures in nonlesional PSO and AD samples, as well as up-regulation of the IL-17 complex signature in nonlesional DLE as compared to control samples, suggesting that IL-17 targeting might be appropriate to prevent the emergence of typical skin lesions in all three diseases as well as to treat established plaques in lesional PSO. Of note, two of five lesional DLE datasets demonstrated significant up-regulation of the IL-17 complex and T_H_17 signature, suggesting that a subset of DLE patients might be responsive to IL-17 neutralization using therapies such as secukinumab, ixekizumab, or brodalumab. A study investigating the role of secukinumab, a monoclonal antibody to IL-17a, in DLE was initiated but discontinued because of difficulty recruiting patients (*53*). In addition, the consistent up-regulation of the TNF signature in each lesional inflammatory skin disease supports the possibility that TNF-neutralizing agents may ameliorate inflammation in all four conditions. To date, TNF-neutralizing agents are effective in treating PSO (*26*) [etanercept (*54*), infliximab (*55*), adalimumab (*56*), and certolizumab pegol (*57*)], whereas others report their possible efficacy in SLE (*58*) and AD (*59*, *60*). Notably, a recent phase 2 trial found that intradermal injection of a TNF-neutralizing agent, etanercept, as opposed to traditional systemic injection, induced remission in DLE (*61*, *62*), supporting the conclusion that the local presence of TNF in the skin lesion is pathogenic in DLE. In a subset of patients with SCLE, TNF blockade has also been a useful treatment option. Nevertheless, it is important to note that anti-TNF–induced lupus or lupus-like syndromes have been reported in some autoimmune disease patients receiving systemic TNF blockade (*58*). Although relatively uncommon (*58*), this finding may caution the use of TNF-neutralizing agents in lupus. Moreover, the IL-12 signature was important in classifying all four lesional skin diseases, suggesting potential efficacy of the IL-12/23 inhibitor, ustekinumab, which is approved for treating PSO (*63*). Recent phase 3 trials in lupus were unsuccessful (*64*, *65*), but improvement of skin and mucocutaneous lesions was noted in phase 2 trials (*66*). Last, consistent enrichment of the IGS was observed in lesional skin of all four diseases, suggesting the potential for efficacy of IFN inhibitors such as anifrolumab. Treatment with anifrolumab, which was recently approved for SLE, resulted in a significant reduction in skin involvement in CLE compared to patients receiving placebo (*7*). Together, our data suggest that there are several available therapeutic targets among the common mechanisms up-regulated in these four lesional inflammatory skin diseases, including the IL-12 complex, TNF, IFN, and the IL-23 complex. In addition, there are potential therapeutics noted that are not currently approved in the specific diseases stated but may prove useful based on the shared molecular features of both nonlesional and lesional inflammatory skin conditions (Fig. 8B).

We recognize the limitations of this study. First, some of the individual datasets had small sample numbers; therefore, it was necessary to pool lesional samples from each disease, nonlesional samples from each disease, and controls to achieve sufficient sample numbers for ML. In addition, some datasets had few or no controls, and thereby, nonlesional skin could not be compared to control samples by GSVA without the pooling and normalization of samples. Nevertheless, we found a number of changes in nonlesional DLE similar to those previously reported by other techniques, for example, the decrease in Langerhans cells via immunohistochemistry (*67*). Moreover, because many of the widely used keratinocyte gene signatures were highly correlated with each other, ML analysis with these gene signatures previously reported in PSO and AD was not possible (*30*, *68*, *69*). Despite these caveats and the inter- and intra-dataset heterogeneity, we identified gene signatures both similar and distinct in lesional and nonlesional inflammatory skin diseases.

In summary, this transcriptomic analysis is one of the first comprehensive studies to evaluate four inflammatory skin diseases concurrently and introduce comparative analyses of both lesional and nonlesional samples with control samples. We elucidated similarities and differences among both lesional and nonlesional DLE, PSO, AD, and SSc. Overall, our combined GSVA/ML analysis demonstrated that although there are seven shared features for classifying lesional DLE, PSO, AD, and SSc from pooled controls, molecular features of nonlesional skin samples are more distinct. This indicates that nonlesional skin samples are extremely informative about the underlying disease process and could be used to subset patients for future clinical trials or de-risk clinical trials, as nonlesional skin is reported to be an effective marker of treatment response (*70*). Nonlesional skin may be more useful in identifying the driving features underlying pathogenesis because the inflammatory milieu among diseases becomes more similar during chronic lesional disease. In addition, although enrichment analysis of all cell types and pathways is important in the overall definition of disease pathology and is necessary to understand for treatment decisions, determination of discriminatory features may be more important in molecular diagnostics for inflammatory skin diseases or additional pathologies. ML can be used to identify these features, which could aid in diagnosis and also help inform potential pathogenic drivers or contributors. Together, the application of gene signature analysis and the multiple computational approaches, similar to those used here, is informative for understanding disease pathogenesis and could be useful for investigation of the transcriptomic landscape of many conditions.

## MATERIALS AND METHODS

### Experimental design

Fifteen publicly available gene expression datasets [accessed from the Gene Expression Omnibus (GEO)] were analyzed (table S1), including 11 Affymetrix/Illumina microarray datasets (GSE52471, GSE72535, GSE81071, GSE109248, GSE100093, GSE120809, GSE117239, GSE117468, GSE130588, GSE58095, and GSE95065) and 4 RNA-seq datasets (GSE121212, GSE137430, GSE157194, and GSE130955). GSE81071 was split into two parts based on the submission date on GEO (GSE81071 from 2017 referred to in the text as GSE81071_A and GSE81071 from 2019 referred to in the text as GSE81071_B). All datasets comprise gene expression derived from skin biopsies of lesional or nonlesional skin from patients with an inflammatory skin disease, including PSO, AD, SSc, and CLE subtypes including DLE, SCLE, and ACLE or skin biopsies derived from healthy control subjects. For GSE117239, GSE117468, GSE130588, GSE137430, and GSE157194, only lesional and nonlesional (clinically uninvolved) samples at baseline without drug treatment were included in the analysis.

### Statistical analysis

Statistical differences between cohorts were evaluated using unpaired *t* test with Welch’s correction for GSVA enrichment scores of lesional and nonlesional samples, mean *z* scores of nonlesional samples versus control, and paired *t* test with Welch’s correction for lesional versus nonlesional comparison and were carried out in GraphPad Prism. Calculation of mean and SD for each GSVA score in each tissue was performed in Microsoft Excel. The number of samples for each dataset was detailed in table S3B. Further statistical details can be found in Supplementary Materials and Methods.

## Supplementary Materials

### This PDF file includes:

Supplementary Methods Figs. S1 to S26 Tables S1 to S4 References

## Notes

View/request a protocol for this paper from *Bio-protocol*.

## References

[1] B. Tebbe, C. Orfanos, Epidemiology and socioeconomic impact of skin disease in lupus erythematosus. Lupus 6, 96–104 (1997). 9061657 10.1177/096120339700600204

[2] M. P. Maz, J. Michelle Kahlenberg, Cutaneous and systemic connections in lupus. Curr. Opin. Rheumatol. 32, 583–589 (2020). 32826479 10.1097/BOR.0000000000000739 PMC8000781

[3] L. Uva, D. Miguel, C. Pinheiro, J. P. Freitas, M. Marques Gomes, P. Filipe, Cutaneous manifestations of systemic lupus erythematosus. Autoimmune Dis. 2012, 834291 (2012). 22888407 10.1155/2012/834291 PMC3410306

[4] J. Wenzel, Cutaneous lupus erythematosus: New insights into pathogenesis and therapeutic strategies. Nat. Rev. Rheumatol. 15, 519–532 (2019). 31399711 10.1038/s41584-019-0272-0

[5] S. Ribero, S. Sciascia, L. Borradori, D. Lipsker, The cutaneous spectrum of lupus erythematosus. Clin. Rev. Allergy Immunol. 53, 291–305 (2017). 28752372 10.1007/s12016-017-8627-2

[6] P. Vashisht, K. Borghoff, J. R. O’Dell, M. Hearth-Holmes, Belimumab for the treatment of recalcitrant cutaneous lupus. Lupus 26, 857–864 (2017). 28121495 10.1177/0961203316682097

[7] E. F. Morand, R. Furie, Y. Tanaka, I. N. Bruce, A. D. Askanase, C. Richez, S.-C. Bae, P. Z. Brohawn, L. Pineda, A. Berglind, R. Tummala, Trial of anifrolumab in active systemic lupus erythematosus. N. Engl. J. Med. 382, 211–221 (2020). 31851795 10.1056/NEJMoa1912196

[8] A. Menter, B. E. Strober, D. H. Kaplan, D. Kivelevitch, E. F. Prater, B. Stoff, A. W. Armstrong, C. Connor, K. M. Cordoro, D. M. R. Davis, B. E. Elewski, J. M. Gelfand, K. B. Gordon, A. B. Gottlieb, A. Kavanaugh, M. Kiselica, N. J. Korman, D. Kroshinsky, M. Lebwohl, C. L. Leonardi, J. Lichten, H. W. Lim, N. N. Mehta, A. S. Paller, S. L. Parra, A. L. Pathy, R. N. Rupani, M. Siegel, E. B. Wong, J. J. Wu, V. Hariharan, C. A. Elmets, Joint AAD-NPF guidelines of care for the management and treatment of psoriasis with biologics. J. Am. Acad. Dermatol. 80, 1029–1072 (2019). 30772098 10.1016/j.jaad.2018.11.057

[9] D. Deleanu, I. Nedelea, Biological therapies for atopic dermatitis: An update. Exp. Ther. Med. 17, 1061–1067 (2019). 30679974 10.3892/etm.2018.6989 PMC6327672

[10] A. Jabbari, M. Suárez-Fariñas, J. Fuentes-Duculan, J. Gonzalez, I. Cueto, A. G. Franks, J. G. Krueger, Dominant Th1 and minimal Th17 skewing in discoid lupus revealed by transcriptomic comparison with psoriasis. J. Invest. Dermatol. 134, 87–95 (2014). 23771123 10.1038/jid.2013.269 PMC3858414

[11] B. F. Chong, L. C. Tseng, G. A. Hosler, N. M. Teske, S. Zhang, D. R. Karp, N. J. Olsen, C. Mohan, A subset of CD163^+^ macrophages displays mixed polarizations in discoid lupus skin. Arthritis Res. Ther. 17, 324 (2015). 26568320 10.1186/s13075-015-0839-3 PMC4644297

[12] A. M. S. Barron, J. C. Mantero, J. D. Ho, B. Nazari, K. L. Horback, J. Bhawan, R. Lafyatis, C. Lam, J. L. Browning, Perivascular adventitial fibroblast specialization accompanies T Cell retention in the inflamed human dermis. J. Immunol. 202, 56–68 (2019). 30510068 10.4049/jimmunol.1801209 PMC6305793

[13] P. Mande, B. Zirak, W. C. Ko, K. Taravati, K. L. Bride, T. Y. Brodeur, A. Deng, K. Dresser, Z. Jiang, R. Ettinger, K. A. Fitzgerald, M. D. Rosenblum, J. E. Harris, A. Marshak-Rothstein, Fas ligand promotes an inducible TLR-dependent model of cutaneous lupus–like inflammation. J. Clin. Invest. 128, 2966–2978 (2018). 29889098 10.1172/JCI98219 PMC6025993

[14] J. Liu, C. C. Berthier, J. M. Kahlenberg, Enhanced inflammasome activity in systemic lupus erythematosus is mediated via type I interferon–induced up-regulation of interferon regulatory factor 1. Arthritis Rheumatol. 69, 1840–1849 (2017). 28564495 10.1002/art.40166 PMC5575977

[15] L. C. Tsoi, G. A. Hile, C. C. Berthier, M. K. Sarkar, T. J. Reed, J. Liu, R. Uppala, M. Patrick, K. Raja, X. Xing, E. Xing, K. He, J. E. Gudjonsson, J. M. Kahlenberg, Hypersensitive IFN responses in lupus keratinocytes reveal key mechanistic determinants in cutaneous lupus. J. Immunol. 202, 2121–2130 (2019). 30745462 10.4049/jimmunol.1800650 PMC6424612

[16] V. P. Werth, D. Fiorentino, B. A. Sullivan, M. J. Boedigheimer, K. Chiu, C. Wang, G. E. Arnold, M. A. Damore, J. Bigler, A. A. Welcher, C. B. Russell, D. A. Martin, J. B. Chung, Brief Report: Pharmacodynamics, safety, and clinical efficacy of AMG 811, a human anti–interferon-γ antibody, in patients with discoid lupus erythematosus. Arthritis Rheumatol. 69, 1028–1034 (2017). 28118537 10.1002/art.40052 PMC5434930

[17] M. D. Catalina, P. Bachali, N. S. Geraci, A. C. Grammer, P. E. Lipsky, Gene expression analysis delineates the potential roles of multiple interferons in systemic lupus erythematosus. Commun. Biol. 2, 140 (2019). 31044165 10.1038/s42003-019-0382-x PMC6478921

[18] K. E. Nograles, L. C. Zaba, E. Guttman-Yassky, J. Fuentes-Duculan, M. Suárez-Fariñas, I. Cardinale, A. Khatcherian, J. Gonzalez, K. C. Pierson, T. R. White, C. Pensabene, I. Coats, I. Novitskaya, M. A. Lowes, J. G. Krueger, Th17 cytokines interleukin (IL)-17 and IL-22 modulate distinct inflammatory and keratinocyte-response pathways. Br. J. Dermatol. 159, 1092–1102 (2008). 18684158 10.1111/j.1365-2133.2008.08769.x PMC2724264

[19] Y. Asano, Systemic sclerosis. J. Dermatol. 45, 128–138 (2018). 29226387 10.1111/1346-8138.14153

[20] P. M. Brunner, E. Guttman-Yassky, D. Y. M. Leung, The immunology of atopic dermatitis and its reversibility with broad-spectrum and targeted therapies. J. Allergy Clin. Immunol. 139, S65–S76 (2017). 28390479 10.1016/j.jaci.2017.01.011 PMC5405702

[21] K. M. Kingsmore, P. Bachali, M. D. Catalina, A. R. Daamen, S. E. Heuer, R. D. Robl, A. C. Grammer, P. E. Lipsky, Altered expression of genes controlling metabolism characterizes the tissue response to immune injury in lupus. Sci. Rep. 11, 14789 (2021). 34285256 10.1038/s41598-021-93034-w PMC8292402

[22] S. J. Waddell, S. J. Popper, K. H. Rubins, M. J. Griffiths, P. O. Brown, M. Levin, D. A. Relman, Dissecting interferon-induced transcriptional programs in human peripheral blood cells. PLOS ONE 5, e9753 (2010). 20339534 10.1371/journal.pone.0009753 PMC2842296

[23] J. L. Sargent, A. Milano, S. Bhattacharyya, J. Varga, M. K. Connolly, H. Y. Chang, M. L. Whitfield, A TGFβ-responsive gene signature is associated with a subset of diffuse scleroderma with increased disease severity. J. Invest. Dermatol. 130, 694–705 (2010). 19812599 10.1038/jid.2009.318 PMC3867816

[24] C. L. Langrish, Y. Chen, W. M. Blumenschein, J. Mattson, B. Basham, J. D. Sedgwick, T. McClanahan, R. A. Kastelein, D. J. Cua, IL-23 drives a pathogenic T cell population that induces autoimmune inflammation. J. Exp. Med. 201, 233–240 (2005). 15657292 10.1084/jem.20041257 PMC2212798

[25] A. Blauvelt, A. Chiricozzi, The immunologic role of IL-17 in psoriasis and psoriatic arthritis pathogenesis. Clin. Rev. Allergy Immunol. 55, 379–390 (2018). 30109481 10.1007/s12016-018-8702-3 PMC6244934

[26] J. E. Hawkes, B. Y. Yan, T. C. Chan, J. G. Krueger, Discovery of the IL-23/IL-17 signaling pathway and the treatment of psoriasis. J. Immunol. 201, 1605–1613 (2018). 30181299 10.4049/jimmunol.1800013 PMC6129988

[27] M. D. Catalina, P. Bachali, A. E. Yeo, N. S. Geraci, M. A. Petri, A. C. Grammer, P. E. Lipsky, Patient ancestry significantly contributes to molecular heterogeneity of systemic lupus erythematosus. JCI Insight 5, e140380 (2020). 32759501 10.1172/jci.insight.140380 PMC7455079

[28] T. Vazquez, R. Feng, K. J. Williams, V. P. Werth, Immunological and clinical heterogeneity in cutaneous lupus erythematosus. Br. J. Dermatol. 185, 481–483 (2021). 33966286 10.1111/bjd.20085

[29] J. L. Zhu, L. T. Tran, M. Smith, F. Zheng, L. Cai, J. A. James, J. M. Guthridge, B. F. Chong, Modular gene analysis reveals distinct molecular signatures for subsets of patients with cutaneous lupus erythematosus. Br. J. Dermatol. 185, 563–572 (2021). 33400293 10.1111/bjd.19800 PMC8255330

[30] L. C. Tsoi, E. Rodriguez, F. Degenhardt, H. Baurecht, U. Wehkamp, N. Volks, S. Szymczak, W. R. Swindell, M. K. Sarkar, K. Raja, S. Shao, M. Patrick, Y. Gao, R. Uppala, B. E. Perez White, S. Getsios, P. W. Harms, E. Maverakis, J. T. Elder, A. Franke, J. E. Gudjonsson, S. Weidinger, Atopic dermatitis is an IL-13–Dominant disease with greater molecular heterogeneity compared to psoriasis. J. Invest. Dermatol. 139, 1480–1489 (2019). 30641038 10.1016/j.jid.2018.12.018 PMC6711380

[31] H. Valdimarsson, J. E. Gudjonsson, A. Johnston, H. Sigmundsdottir, H. Valdimarsson, Immunopathogenic mechanisms in psoriasis. Clin. Exp. Immunol. 135, 1–8 (2004). 14678257 10.1111/j.1365-2249.2004.02310.x PMC1808928

[32] L. Pasquali, A. Srivastava, F. Meisgen, K. Das Mahapatra, P. Xia, N. X. Landén, A. Pivarcsi, E. Sonkoly, The keratinocyte transcriptome in psoriasis: Pathways related to immune responses, cell cycle and keratinization. Acta Derm. Venereol. 99, 196–205 (2019). 30320872 10.2340/00015555-3066

[33] H. Jin, M. K. Oyoshi, Y. Le, T. Bianchi, S. Koduru, C. B. Mathias, L. Kumar, S. Le Bras, D. Young, M. Collins, M. J. Grusby, J. Wenzel, T. Bieber, M. Boes, L. E. Silberstein, H. C. Oettgen, R. S. Geha, IL-21R is essential for epicutaneous sensitization and allergic skin inflammation in humans and mice. J. Clin. Invest. 119, 47–60 (2009). 19075398 10.1172/JCI32310 PMC2613448

[34] F. Gong, Q. Su, Y. H. Pan, X. Huang, W. H. Shen, The emerging role of interleukin-21 in allergic diseases (Review). Biomed. Rep. 1, 837–839 (2013). 24649038 10.3892/br.2013.166 PMC3917548

[35] A. P. Sappino, I. Masouye, J. H. Saurat, G. Gabbiani, Smooth muscle differentiation in scleroderma fibroblastic cells. Am. J. Pathol. 137, 585–591 (1990). 1698026 PMC1877526

[36] J. D’Orazio, S. Jarrett, A. Amaro-Ortiz, T. Scott, UV radiation and the skin. Int. J. Mol. Sci. 14, 12222–12248 (2013). 23749111 10.3390/ijms140612222 PMC3709783

[37] M. Laporte, P. Galand, D. Fokan, C. De Graef, M. Heenen, Apoptosis in established and healing psoriasis. Dermatology 200, 314–316 (2000). 10894962 10.1159/000018394

[38] A. Trautmann, M. Akdis, S. Klunker, K. Blaser, C. A. Akdis, Role of apoptosis in atopic dermatitis. Int. Arch. Allergy Immunol. 124, 230–232 (2001). 11306978 10.1159/000053720

[39] B. Franz, B. Fritzsching, A. Riehl, N. Oberle, C. D. Klemke, J. Sykora, S. Quick, C. Stumpf, M. Hartmann, A. Enk, T. Ruzicka, P. H. Krammer, E. Suri-Payer, A. Kuhn, Low number of regulatory T cells in skin lesions of patients with cutaneous lupus erythematosus. Arthritis Rheum. 56, 1910–1920 (2007). 17530636 10.1002/art.22699

[40] R. Webb, J. T. Merrill, J. A. Kelly, A. Sestak, K. M. Kaufman, C. D. Langefeld, J. Ziegler, P. Robert, J. C. Edberg, R. Ramsey-goldman, M. Petri, J. D. Reveille, G. S. Alarcón, L. M. Vilá, M. E. Alarcón-Riquelme, J. A. James, G. S. Gilkeson, C. O. Jacob, K. L. Moser, P. M. Gaffney, T. J. Vyse, S. K. Nath, P. Lipsky, J. B. Harley, A. H. Sawalha, A polymorphism within interleukin-21 receptor (IL21R) confers risk for systemic lupus erythematosus. Arthritis Rheumatol. 60, 2402–2407 (2009). 10.1002/art.24658 PMC2782592 19644854

[41] A. Pușcaș, A. Cătană, C. Pușcaș, I. Roman, C. Vornicescu, M. Șomlea, R. Orăsan, Psoriasis: Association of interleukin-17 gene polymorphisms with severity and response to treatment (Review). Exp. Ther. Med. 875–880 (2019). 31384317 10.3892/etm.2019.7624 PMC6639965

[42] A. C. Allison Billi, F. Ma, O. Plazyo, M. Gharaee-Kermani, R. Wasikowski, G. A. Hile, X. Xing, C. M. Yee, S. M. Rizvi, M. P. Maz, F. Wen, L. C. Tsoi, M. Pellegrini, R. L. Modlin, J. E. Gudjonsson, J. M. Kahlenberg, A. C. Billi, F. Ma, O. Plazyo, M. G.-Kermani, R. Wasikowski, G. A. Hile, X. Xing, C. M. Yee, S. M. Rizvi, M. P. Maz, F. Wen, L. C. Tsoi, M. Pellegrini, R. L. Modlin, J. E. Gudjonsson, J. M. Kahlenberg, Non-lesional and lesional lupus skin share inflammatory phenotypes that drive activation of CD16^+^ dendritic cells. bioRxiv 2021.09.17.460124 [Preprint]. 20 September 2021. 10.1101/2021.09.17.460124.

[43] E. Der, H. Suryawanshi, P. Morozov, M. Kustagi, B. Goilav, S. Ranabathou, P. Izmirly, R. Clancy, H. M. Belmont, M. Koenigsberg, M. Mokrzycki, H. Rominieki, J. A. Graham, J. P. Rocca, N. Bornkamp, N. Jordan, E. Schulte, M. Wu, J. Pullman, K. Slowikowski, S. Raychaudhuri, J. Guthridge, J. James, J. Buyon, T. Tuschl, C. Putterman, J. Anolik, W. Apruzzese, A. Arazi, C. Berthier, M. Brenner, J. Buyon, R. Clancy, S. Connery, M. Cunningham, M. Dall’Era, A. Davidson, E. Der, A. Fava, C. Fonseka, R. Furie, D. Goldman, R. Gupta, J. Guthridge, N. Hacohen, D. Hildeman, P. Hoover, R. Hsu, J. James, R. Kado, K. Kalunian, D. Kamen, M. Kretzler, H. Maecker, E. Massarotti, W. McCune, M. McMahon, M. Park, F. Payan-Schober, W. Pendergraft, M. Petri, M. Pichavant, C. Putterman, D. Rao, S. Raychaudhuri, K. Slowikowski, H. Suryawanshi, T. Tuschl, P. Utz, D. Waguespack, D. Wofsy, F. Zhang, Tubular cell and keratinocyte single-cell transcriptomics applied to lupus nephritis reveal type I IFN and fibrosis relevant pathways. Nat. Immunol. 20, 915–927 (2019). 31110316 10.1038/s41590-019-0386-1 PMC6584054

[44] T. M. Li, K. R. Veiga, N. Schwartz, Y. Chinenov, D. J. Oliver, J. Lora, A. Jabbari, Y. Liu, W. D. Shipman, M. J. Sandoval, I. F. Sollohub, W. G. Ambler, M. Rashighi, J. G. Krueger, N. Anandasabapathy, C. P. Blobel, T. T. Lu, Type I interferon modulates Langerhans cell ADAM17 to promote photosensitivity in lupus. bioRxiv 2021.08.18.456792 [Preprint]. 18 August 2021. 10.1101/2021.08.18.456792.

[45] K. A. Kirou, C. Lee, S. George, K. Louca, M. G. E. Peterson, M. K. Crow, Activation of the interferon-α pathway identifies a subgroup of systemic lupus erythematosus patients with distinct serologic features and active disease. Arthritis Rheum. 52, 1491–1503 (2005). 15880830 10.1002/art.21031

[46] Q. Z. Li, J. Zhou, Y. Lian, B. Zhang, V. K. Branch, F. Carr-Johnson, D. R. Karp, C. Mohan, E. K. Wakeland, N. J. Olsen, Interferon signature gene expression is correlated with autoantibody profiles in patients with incomplete lupus syndromes. Clin. Exp. Immunol. 159, 281–291 (2010). 19968664 10.1111/j.1365-2249.2009.04057.x PMC2819494

[47] E. L. Hubbard, D. S. Pisetsky, P. E. Lipsky, Anti-RNP antibodies are associated with the interferon gene signature but not decreased complement levels in SLE. Ann. Rheum. Dis., (2022). 10.1136/annrheumdis-2021-221662 35115332

[48] C. C. Berthier, L. C. Tsoi, T. J. Reed, J. N. Stannard, E. M. Myers, R. Namas, X. Xing, S. Lazar, L. Lowe, M. Kretzler, J. E. Gudjonsson, J. M. Kahlenberg, Molecular profiling of cutaneous lupus lesions identifies subgroups distinct from clinical phenotypes. J. Clin. Med. 8, 1244 (2019). 31426521 10.3390/jcm8081244 PMC6723404

[49] S. Zampieri, M. Alaibac, L. Iaccarino, R. Rondinone, A. Ghirardello, P. Sarzi-Puttini, A. Peserico, A. Doria, Tumour necrosis factor α is expressed in refractory skin lesions from patients with subacute cutaneous lupus erythematosus. Ann. Rheum. Dis. 65, 545–548 (2006). 16096331 10.1136/ard.2005.039362 PMC1798098

[50] L. E. Tomalin, C. B. Russell, S. Garcet, D. A. Ewald, P. Klekotka, A. Nirula, H. Norsgaard, M. Suàrez-Fariñas, J. G. Krueger, Short-term transcriptional response to IL-17 receptor-A antagonism in the treatment of psoriasis. J. Allergy Clin. Immunol. 145, 922–932 (2020). 31883845 10.1016/j.jaci.2019.10.041

[51] M. Robert, P. Miossec, Interleukin-17 and lupus: Enough to be a target? For which patients? Lupus 29, 6–14 (2020). 31791181 10.1177/0961203319891243

[52] A study to assess the safety and efficacy of secukinumab in alleviating symptoms of discoid lupus erythematosus. U.S. Natl. Libr. Med. Clin. Trials (2021).

[53] B. Ungar, A. B. Pavel, R. Li, G. Kimmel, J. Nia, P. Hashim, H. J. Kim, M. Chima, A. S. Vekaria, Y. Estrada, H. Xu, X. Peng, G. K. Singer, D. Baum, Y. Mansouri, M. Taliercio, E. Guttman-Yassky, Phase 2 randomized, double-blind study of IL-17 targeting with secukinumab in atopic dermatitis. J. Allergy Clin. Immunol. 147, 394–397 (2021). 32428528 10.1016/j.jaci.2020.04.055

[54] S. Tyring, A. Gottlieb, K. Papp, K. Gordon, C. Leonardi, A. Wang, D. Lalla, M. Woolley, A. Jahreis, R. Zitnik, D. Cella, R. Krishnan, Etanercept and clinical outcomes, fatigue, and depression in psoriasis: Double-blind placebo-controlled randomised phase III trial. Lancet 367, 29–35 (2006). 16399150 10.1016/S0140-6736(05)67763-X

[55] K. Reich, F. O. Nestle, K. Papp, J. P. Ortonne, R. Evans, C. Guzzo, S. Li, L. T. Dooley, C. E. M. Griffiths, Infliximab induction and maintenance therapy for moderate-to-severe psoriasis: A phase III, multicentre, double-blind trial. Lancet 366, 1367–1374 (2005). 16226614 10.1016/S0140-6736(05)67566-6

[56] A. Menter, S. K. Tyring, K. Gordon, A. B. Kimball, C. L. Leonardi, R. G. Langley, B. E. Strober, M. Kaul, Y. Gu, M. Okun, K. Papp, Adalimumab therapy for moderate to severe psoriasis: A randomized, controlled phase III trial. J. Am. Acad. Dermatol. 58, 106–115 (2008). 17936411 10.1016/j.jaad.2007.09.010

[57] A. Blauvelt, K. Reich, M. Lebwohl, D. Burge, C. Arendt, L. Peterson, J. Drew, R. Rolleri, A. B. Gottlieb, Certolizumab pegol for the treatment of patients with moderate-to-severe chronic plaque psoriasis: Pooled analysis of week 16 data from three randomized controlled trials. J. Eur. Acad. Dermatol. Venereol. 33, 546–552 (2019). 30242918 10.1111/jdv.15258 PMC6646900

[58] A. Lorenzo-Vizcaya, D. A. Isenberg, The use of anti-TNF-alpha therapies for patients with systemic lupus erythematosus. Where are we now? Expert Opin. Biol. Ther. 21, 639–647 (2021). 33216641 10.1080/14712598.2021.1853096

[59] A. Jacobi, C. Antoni, B. Manger, G. Schuler, M. Hertl, Infliximab in the treatment of moderate to severe atopic dermatitis. J. Am. Acad. Dermatol. 52, 522–526 (2005). 15761436 10.1016/j.jaad.2004.11.022

[60] N. Cassano, F. Loconsole, C. Coviello, Infliximab in recalcitrant severe atopic eczema associated with contact allergy. Int. J. Immunopathol. Pharmacol. 19, 237–240 (2006). 16569363

[61] M. Yuzaiful, M. Yusof, M. Wittmann, C. Fernandez, D. Wilson, S. Edward, G. Abignano, A. Alase, P. Laws, M. Goodfield, P. Emery, E. Vita, Targeted therapy using intradermal injection of etanercept for remission induction in discoid lupus erythematosus (TARGET-DLE): Results from a proof-of-concept phase II trial. Lupus Sci. Med. 6, A1–A227 (2019).

[62] Targeted therapy using intradermal injection of etanercept for remission induction in discoid lupus erythematosus ( TARGET-DLE ). U.S. Natl. Libr. Med. Clin. Trials (2019).

[63] A. B. Gottlieb, A. M. Goldminz, Ustekinumab for psoriasis and psoriatic arthritis. J. Rheumatol. 39, 86–89 (2012). 22751602 10.3899/jrheum.120253

[64] A study of ustekinumab in participants with active systemic lupus erythematosus (2021); https://clinicaltrials.gov/ct2/show/NCT03517722?term=ustekinumab&cond=lupus&draw=2&rank=3.

[65] Janssen Pharmaceuticals, Janssen announces discontinuation of phase 3 LOTUS study evaluating ustekinumab in systemic lupus erythematosus (2020); www.jnj.com/janssen-announces-discontinuation-of-phase-3-lotus-study-evaluating-ustekinumab-in-systemic-lupus-erythematosus.

[66] R. F. van Vollenhoven, B. H. Hahn, G. C. Tsokos, C. L. Wagner, P. Lipsky, Z. Touma, V. P. Werth, R. M. Gordon, B. Zhou, B. Hsu, M. Chevrier, M. Triebel, J. L. Jordan, S. Rose, Efficacy and safety of ustekinumab, an IL-12 and IL-23 inhibitor, in patients with active systemic lupus erythematosus: Results of a multicentre, double-blind, phase 2, randomised, controlled study. Lancet 392, 1330–1339 (2018). 30249507 10.1016/S0140-6736(18)32167-6

[67] W. D. Shipman, S. Chyou, A. Ramanathan, P. M. Izmirly, S. Sharma, T. Pannellini, D. C. Dasoveanu, X. Qing, C. M. Magro, R. D. Granstein, M. A. Lowes, E. G. Pamer, D. H. Kaplan, J. E. Salmon, B. J. Mehrara, J. W. Young, R. M. Clancy, C. P. Blobel, T. T. Lu, A protective Langerhans cell keratinocyte axis that is dysfunctional in photosensitivity. Sci. Transl. Med. 10, eaap9527 (2018). 30111646 10.1126/scitranslmed.aap9527 PMC6365282

[68] S. Tian, J. G. Krueger, K. Li, A. Jabbari, C. Brodmerkel, M. A. Lowes, M. Suárez-Fariñas, Meta-analysis derived (MAD) transcriptome of psoriasis defines the “Core” pathogenesis of disease. PLOS ONE 7, e44274 (2012). 22957057 10.1371/journal.pone.0044274 PMC3434204

[69] L. Möbus, E. Rodriguez, I. Harder, D. Stölzl, N. Boraczynski, S. Gerdes, A. Kleinheinz, S. Abraham, A. Heratizadeh, C. Handrick, E. Haufe, T. Werfel, J. Schmitt, S. Weidinger, Atopic dermatitis displays stable and dynamic skin transcriptome signatures. J. Allergy Clin. Immunol. 147, 213–223 (2021). 32615169 10.1016/j.jaci.2020.06.012

[70] L. C. Tsoi, M. T. Patrick, S. Shuai, M. K. Sarkar, S. Chi, B. Ruffino, A. C. Billi, X. Xing, R. Uppala, C. Zang, J. Fullmer, Z. He, E. Maverakis, N. N. Mehta, B. E. Perez White, S. Getsios, Y. Helfrich, J. J. Voorhees, J. M. Kahlenberg, S. Weidinger, J. E. Gudjonsson, Cytokine responses in nonlesional psoriatic skin as clinical predictor to anti-TNF agents. J. Allergy Clin. Immunol. 75, 15–18 (2021). 10.1016/j.jaci.2021.07.024 PMC9451046 34343561

[71] E. Eisenberg, E. Y. Levanon, Human housekeeping genes, revisited. Trends Genet. 29, 569–574 (2013). 23810203 10.1016/j.tig.2013.05.010

[72] S. Hänzelmann, R. Castelo, J. Guinney, GSVA: Gene set variation analysis for microarray and RNA-Seq data (2013); www.biomedcentral.com/1471-2105/14/7http://www.bioconductor.org.Background. 10.1186/1471-2105-14-7 PMC3618321 23323831

[73] C. Cheadle, M. P. Vawter, W. J. Freed, K. G. Becker, Analysis of microarray data using z score transformation. J. Mol. Diagn. 5, 73–81 12707371 10.1016/S1525-1578(10)60455-2 PMC1907322

[74] J. Menche, E. Guney, A. Sharma, P. J. Branigan, M. J. Loza, F. Baribaud, R. Dobrin, A. L. Barabási, Integrating personalized gene expression profiles into predictive disease-associated gene pools. npj Syst. Biol. Appl. 3, 10 (2017). 28649437 10.1038/s41540-017-0009-0 PMC5445628

[75] M. Uhlén, L. Fagerberg, B. M. Hallström, C. Lindskog, P. Oksvold, A. Mardinoglu, Å. Sivertsson, C. Kampf, E. Sjöstedt, A. Asplund, I. M. Olsson, K. Edlund, E. Lundberg, S. Navani, C. A. K. Szigyarto, J. Odeberg, D. Djureinovic, J. O. Takanen, S. Hober, T. Alm, P. H. Edqvist, H. Berling, H. Tegel, J. Mulder, J. Rockberg, P. Nilsson, J. M. Schwenk, M. Hamsten, K. Von Feilitzen, M. Forsberg, L. Persson, F. Johansson, M. Zwahlen, G. Von Heijne, J. Nielsen, F. Pontén, Tissue-based map of the human proteome. Science 347, 394 (2015). 10.1126/science.1260419 25613900

[76] A. Gazel, P. Ramphal, M. Rosdy, B. De Wever, C. Tornier, N. Hosein, B. Lee, M. Tomic-Canic, M. Blumenberg, Transcriptional profiling of epidermal keratinocytes: Comparison of genes expressed in skin, cultured keratinocytes, and reconstituted epidermis, using large DNA microarrays. J. Invest. Dermatol. 121, 1459–1468 (2003). 14675197 10.1111/j.1523-1747.2003.12611.x

[77] L. Breiman, J. H. Friedman, R. A. Olshen, C. J. Stone, *Classification and Regression Trees* (Chapman & Hall/CRC Taylor & Francis Group, 1984).

[78] L. Breiman, Random forests. Mach. Learn. 45, 5–32 (2001).

[79] R. Blagus, L. Lusa, SMOTE for high-dimensional class-imbalanced data. BMC Bioinformatics 14, 106 (2013). 23522326 10.1186/1471-2105-14-106 PMC3648438

[80] Z. Gu, R. Eils, M. Schlesner, Complex heatmaps reveal patterns and correlations in multidimensional genomic data. Bioinformatics 32, 2847–2849 (2016). 27207943 10.1093/bioinformatics/btw313

