# Supplementary material for: Machine learning reveals distinct gene signature profiles in lesional and nonlesional regions of inflammatory skin diseases
Source: Sci Adv. 2022 Apr 29;8(17):eabn4776. doi: 10.1126/sciadv.abn4776 (PMC9054015; doi:10.1126/sciadv.abn4776)
Supplement: Supplementary file 1 — Supplementary Methods Figs. S1 to S26 Tables S1 to S4 References [file sciadv.abn4776_sm.pdf]

Science Advances
AAAS

Supplementary Materials for

Machine learning reveals distinct gene signature profiles in lesional and nonlesional regions of inflammatory skin diseases

Brittany A. Martínez*, Sneha Shrotri, Kathryn M. Kingsmore, Prathyusha Bachali, Amrie C. Grammer, Peter E. Lipsky*

*Corresponding author. Email: brittany.martinez@ampelbiosolutions.com (B.A.M.); peterlipsky@comcast.net (P.E.L.)

Published 29 April 2022, *Sci. Adv.* **8**, eabn4776 (2022)
DOI: 10.1126/sciadv.abn4776

This PDF file includes:

Supplementary Methods
Figs. S1 to S26
Tables S1 to S4
References

Supplementary Methods

Raw data processing:

Microarray data: Microarray data was normalized using either GeneChip Robust Multiarray Average (GCRMA), Robust Multiarray Average (RMA), or normexp background correction (NEQC) based on the microarray platform. Outliers and batch effects were identified using principal component analysis (PCA) plots. For the dataset with known batch effects, GSE81071, raw gene expression values were normalized using 11 housekeeping genes, which were shown to not vary significantly across datasets *(71)*. These 11 housekeeping genes were: chromosome 1 Open Reading Frame 43 (*C1orf43*), Charged multivesicular body protein 2A (*CHMP2A*), ER membrane protein complex subunit 7 (*EMC7*), glucose-6-phosphate isomerase (*GPI*), proteosome subunit beta type 2 (*PSMB2*), proteosome subunit beta type 4 (*PSMB4*), member RAS oncogene family (*RAB7A*), receptor accessory protein 5 (*REEP5*), small nuclear ribonucleoprotein D3 (*SNRPD3*), valosin containing protein (*VCP*), and vacuolar protein sorting 29 homolog (*VPS29*).

RNAseq data: SRA toolkit (NCBI Sequence Read Archive, Version 2.10) was used to fetch .sra files from GEO and convert them to.fastq files. Quality of the FASTQ files was checked using FASTQC software (Babraham Institute Bioinformatics, Version 0.11.9). Adapters were removed using Trimmomatic software (Version 0.4) and appropriate head crop parameters. Trimmed reads were aligned to the human reference genome (hg38) using STAR aligner (Version 2.7). STAR output .sam files were converted to .bam files using sambamba (Version 0.8). Read summarization was provided using the featureCounts function of the Subread (Version 2.0) package. Count normalization and regularized log transformation were carried out using rlog function in DESeq2 (Version 1.32) R package.

Gene Set Variation Analysis: Gene Set Variation Analysis *(72)* (GSVA) is a non-parametric, unsupervised method for estimating variations in gene set enrichment among the samples of an expression dataset. The GSVA algorithm was implemented using the R Bioconductor open-source package gsva (version 1.40). GSVA was carried out in one of the following ways:

When individual datasets were analyzed, the preprocessed log2 gene expression matrix of each dataset was used as the GSVA input. GSVA was run on each dataset separately. Before running GSVA, input genes were filtered and only those with interquartile range (IQR) of expression > 0 across all the samples were considered for analysis. All analysis in **Figs. 1-6** are the result of this GSVA process. A minimum of 2 genes was required for each signature.

For the analysis of pooled nonlesional and control samples, log2 gene expression values generated from independent preprocessing of all 16 datasets were concatenated to create a matrix whose rows consisted of 8425 genes detected across all datasets and whose columns consisted of the 1065 samples comprised of DLE, nonlesional DLE, ACLE, SCLE, PSO, nonlesional PSO, AD, nonlesional AD, SSc, and CTLs. Log2 values were then transformed to Z-scores using *scale()* function in R. Z-score transformation converts each sample to have expression values with mean and unit variance equal to 0 *(73, 74)*. This transformation permitted comparison of nonlesional disease samples to control directly. GSVA was then run on the following three inputs 1) 21 pooled nonlesional DLE and 168 pooled control samples, 2) 132 pooled nonlesional AD and 168 pooled control samples, and 3) 163 pooled nonlesional PSO and 168 pooled samples. The data presented in **Fig. 7A** is derived from this GSVA process. A minimum of 2 genes was required for each signature.

GSVA Gene Sets: The gene sets used for GSVA can be found in **tables S2A-D**.

Cellular / pathway signatures: Gene sets employed in our GSVA analysis included 48 annotated and novel cellular and pathway signatures that have been implicated in lupus *(4, 5, 6)* or inflammatory skin diseases *(7, 8)*. Immune cell gene sets were previously evaluated *(21, 27)* or amended slightly based upon data from the Human Protein Atlas *(75)*. Non-hematopoietic cell signatures were derived from the Human Protein Atlas *(75)*, previously published gene sets *(76)*, and literature mining as previously described and employed (**table S2A**). Pathway gene signatures were previously evaluated in lupus *(21, 27)*, previously published *(23, 24)*, or newly adopted by literature mining (**table S2B**). The output GSVA scores of each signature were used as features for training and validating ML classifiers. 40 of the 48 cellular and pathway gene signatures were used to implement the GSVA analysis on pooled nonlesional and control samples. The following signatures were excluded from the pooled nonlesional GSVA analysis because of insufficient gene numbers (</= 2) in the 8,425 genes used: LDG, GC B cell, erythrocyte, IL1 cytokines, IL12 complex, IL21 complex, IL23 complex, and the immunoproteasome.

Keratinocyte signatures: 30 gene sets specific to keratinocytes treated with individual cytokines were created from previously published studies. Only those genes that are upregulated in keratinocytes when treated with various cytokines were included in these sets (**table S2C**).

T cell signatures: Gene sets for T cells were created from literature mining and the Human Protein Atlas *(75)* to distinguish seven different T cell subsets that have been implicated in inflammatory skin disease (**table S2D**).

Classification and Regression Tree (CART): The library rpart (Version 4.1) was used to implement the CART algorithm for classification described previously *(77, 78)* and library

rpart.plot (Version 3.1) was used to visualize classification trees. GSVA enrichment scores of cellular and pathway signatures were used as independent variables and specific lesional disease (either DLE, PSO, AD, SSC, or CTL) was used as the dependent variable for analysis. Classification trees were built independently for each disease.

ML Analysis:

Creating input for ML: The input for ML was created by pooling GSVA enrichment scores of cellular and pathway gene signatures from multiple skin datasets based on sample properties (**table S3B**). For every dataset, GSVA enrichment scores, that range from -1 to +1, were concatenated from multiple datasets, providing a sufficiently large cohort to train and validate various ML algorithms. 14 input data frames were created for 14 separate binary ML classifications (**table S3A**). Seven of the 14 binary classifications involved comparing control samples (164 CTL) with either lesional samples (DLE, PSO, AD or SSc) or nonlesional samples (DLE, PSO or AD) of inflammatory skin diseases (**table S3A A-D** and **I-K**), whereas the other six binary classifications involved comparing lesional DLE samples with lesional samples of other diseases (either PSO, AD or SSc) (**table S3A E-H**) and nonlesional DLE samples with nonlesional samples of other diseases (**table S3A L-M**). In addition, another binary classification compared nonlesional PSO and nonlesional AD (**table S3A**). For lesional skin classification, pooled samples resulted in 90 DLE, 132 AD, 97 SSc, and 183 PSO samples. For nonlesional skin classification, pooled samples resulted in 21 DLE, 163 PSO, and 132 AD samples, and for healthy skin pooled samples were 164 CTL (**table S3B**).

Class balance strategies: Four class balance strategies, including: random undersampling (**table S3A C**), random oversampling (**table S3A E, K**) removing samples from an entire dataset (**table S3A F**), and Synthetic Minority Oversampling Technique (SMOTE) *(79)* (**table S3A I, L, M**) were used for classifications with class imbalance. The random undersampling strategy

involves randomly selecting samples from the majority class, whereas the random oversampling strategy involves randomly duplicating examples from the minority class. SMOTE functions by randomly selecting samples from the minority class, finding its k nearest neighbors, randomly selecting a neighbor, and generating a synthetic sample at a randomly selected point between two samples in the feature space. As previously noted, we used random undersampling to trim the number of examples in the majority class then used SMOTE to oversample the minority class to balance class distribution. The purpose of all class balancing strategies was to have balanced representation of both classes for ML. The dataset was split into 70% training and 30% validation and class balancing strategies were applied on the training dataset. ML algorithms were then implemented, and evaluation matrices were noted. Receiver Operating Characteristic (ROC) curves and Precision-Recall (PR) curves were plotted using the matplotlib (Version 3.3.4) library of Python. A ROC curve is graphical way to visualize trade-off between sensitivity and specificity. High area under the curve represents a low false-positive rate and a high true-positive rate. A PR curve is a measure of classification when classes are imbalanced. High area under the PR curve represents both high recall and high precision, where high precision relates to a low false-positive rate, and high recall relates to a low-false negative rate. For our analysis, we were interested in features that contributed the most towards separation of classes, hence RF was chosen as the primary ML classifier because it gives impurity-based feature importance. The top 15 features with decreasing Gini index from each classification were summarized in a bar graph using ggplots2 (Version 3.3.5) library in R. Capability of the top 15 features alone to separate the two respective classes was tested by repeating the 14 binary ML classifications using only the top 15 features. Various overlaps between the top 15 features of multiple classifications were visualized in Venn diagrams.

Binary ML classification: 14 separate binary ML classifications were carried out using scikit-learn (Version 0.24.1) library in Python (Version 3.8.2). For each binary classification,

performance of several ML algorithms, including: logistic regression (LR), k-nearest neighbor (KNN), naïve Bayes (NB), support vector machines (SVM), random forest (RF), and gradient boosting (GB) was evaluated based on sensitivity, specificity, Cohen's kappa score, f-1 score, and accuracy. RF was chosen as the primary ML classifier because it gives impurity-based feature importance. The top 15 features with decreasing Gini index from each classification were summarized in a bar graph using ggplots2 (Version 3.3.5) library in R. Capability of the top 15 features to separate two respective classes was tested by repeating the 14 binary ML classifications using only the top 15 features.

Feature correlation: Before carrying out binary ML classification, feature selection was necessary in order to remove noninformative or redundant features. We assessed feature redundancy by calculating the Pearson correlation between each feature and every other feature. Pearson correlation between features was computed using the cor function in R. corplot library in R was used to plot 22 Pearson correlation plots (**figs. S7,S10,S14,S16,S25**). In 13 of these correlation plots, there was a pair of highly correlated features (correlation coefficient > 0.8), and the feature with the lower correlation was removed using a greedy elimination approach; this allowed us to retain the most informative features for ML (**table S3A**). Pearson correlation plots were also plotted for keratinocytes gene signatures and T cell signatures (**figs. S6,S12**). High correlation between the keratinocyte gene signatures made them unsuitable for ML analysis (**fig. S6**).

Statistical Analysis: Statistical differences between cohorts were evaluated using Welch's t-test for lesional disease versus control GSVA scores from a single dataset, nonlesional samples versus control GSVA scores from combined datasets, mean Z-scores of nonlesional samples versus mean Z-scores of control samples of a single gene signature and Paired t-test for

lesional versus nonlesional comparison. The magnitude of this difference (the effect size) was estimated using Hedge's g calculated as below.

$$g = \frac{\overline{x_1} - \overline{x_2}}{\sqrt{\frac{(n_1 - 1) * s_1^2 + (n_2 - 1) * s_2^2}{n_1 + n_2 - 2}}}$$

where,

$\overline{x_1}$ *and* $\overline{x_2}$ = *cohort* 1 *mean and cohort* 2 *mean respectively*

n_1 *and* n_2 = *cohort* 1 *size and cohort* 2 *size respectively*

s_1^2 *and* s_2^2 = *cohort* 1 *variance and cohort* 2 *varaince respectively*

cohort 1 and cohort 2 could be either disease and their respective control samples of a single dataset or nonlesional samples and control samples from combined dataset or mean z scores of nonlesional samples and mean Z-scores of control samples of a single gene signature or lesional and their paired nonlesional samples of a single dataset. All the statistical analysis was carried out in using effectSize (version 0.8.1) and stats (version 3.6.2) libraries in R.

Data Visualization: Heatmaps of GSVA Hedges' g effect size and violin plots of GSVA enrichment scores were visualized using GraphPad PRISM (Version 9.2.0). GSVA enrichment scores of gene signatures were visualized using violin plots in Prism or ComplexHeatmap *(80)* for hierarchical clustering (Version 2.8) package in R. Figures were made using Adobe Illustrator Creative Cloud (Version 25.3.1).

Supplemental Figures:

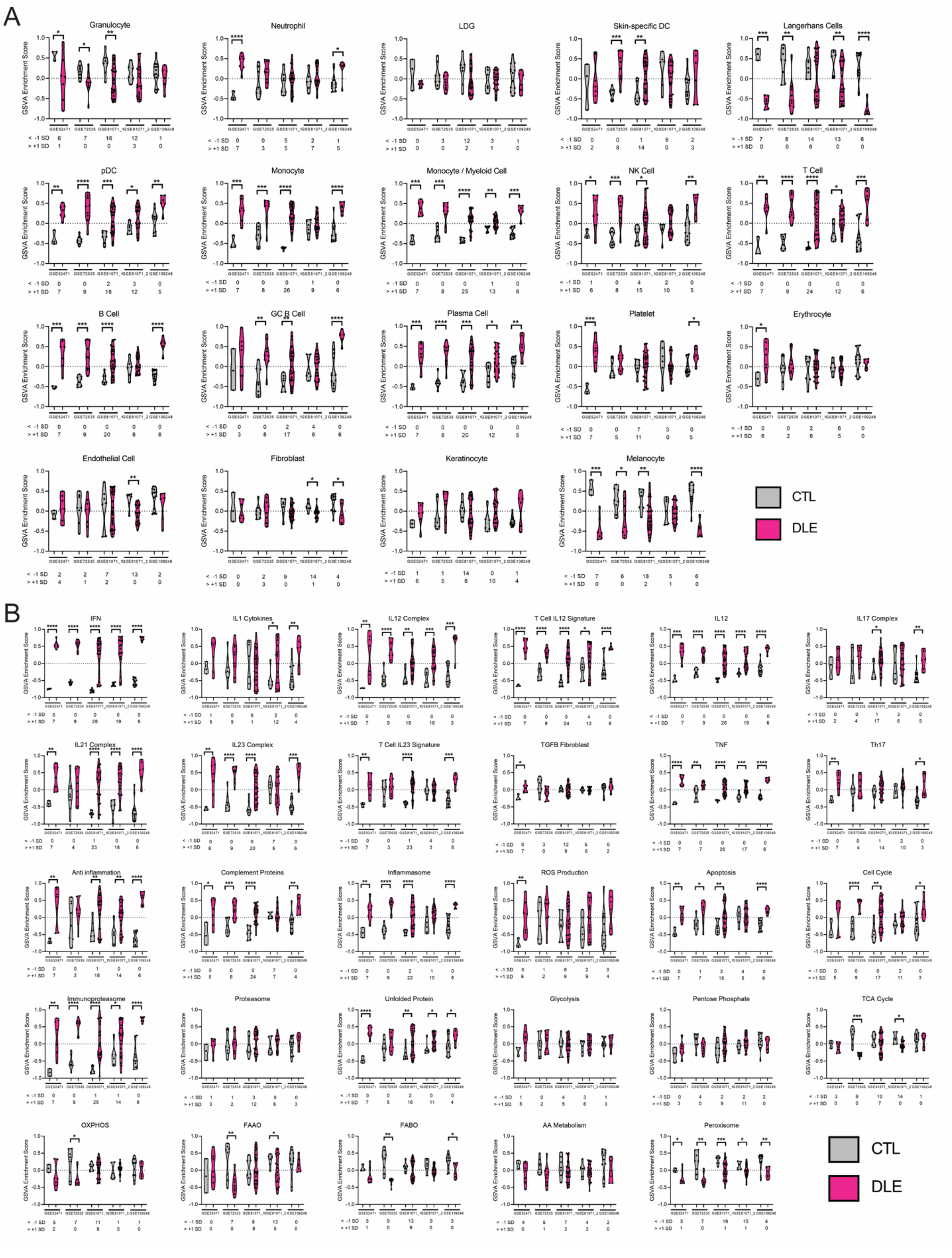

Supplemental Figure 1: Analysis of cellular and molecular pathway signatures in lesional DLE shows increased expression of inflammatory pathways regulated by monocyte, B cell, T cell and plasmacytoid dendritic cell signatures. GSVA enrichment scores of (**A**) cellular gene signatures and (**B**) pathway gene signatures in five datasets including DLE samples (pink) and control samples (grey). The number of DLE samples per dataset that lie -1 standard deviation of the mean of the control samples is denoted on the first subtext line. The number of DLE samples per dataset that lie +1 standard deviation of the mean of the control samples is denoted on the second subtext line. Welch's t-test: * $p < 0.05$; ** $p < 0.01$; *** $p < 0.001$; **** $p < 0.0001$.

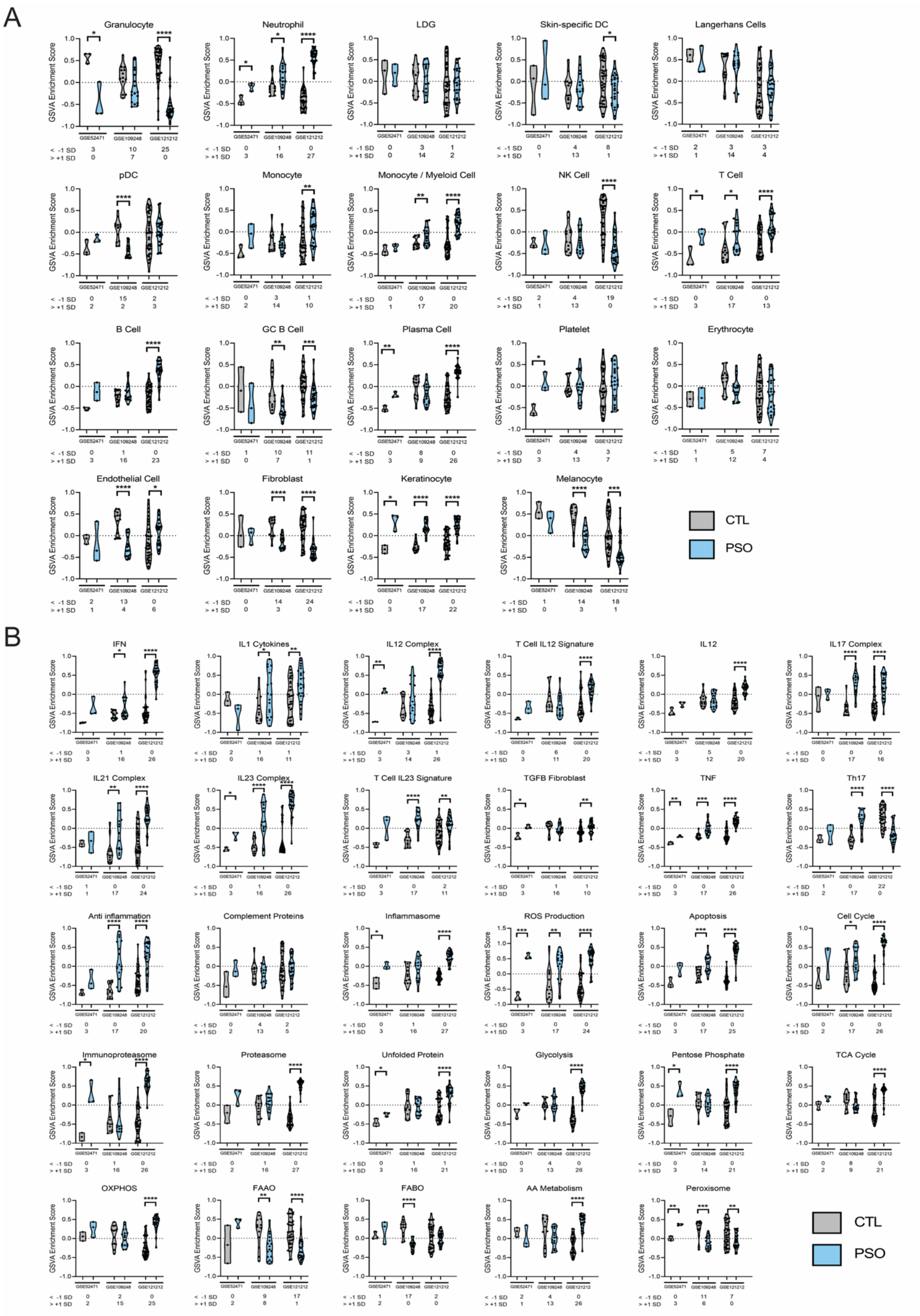
A
Granulocyte
Neutrophil
LDG
Skin-specific DC
Langerhans Cells
pDC
Monocyte
Monocyte / Myeloid Cell
NK Cell
T Cell
B Cell
GC B Cell
Plasma Cell
Platelet
Erythrocyte
Endothelial Cell
Fibroblast
Keratinocyte
Melanocyte
GSVA Enrichment Score
GSE52471
GSE109248
GSE121212
< -1 SD
> +1 SD
CTL
PSO
B
IFN
IL1 Cytokines
IL12 Complex
T Cell IL12 Signature
IL12
IL17 Complex
IL21 Complex
IL23 Complex
T Cell IL23 Signature
TGFB Fibroblast
TNF
Th17
Anti inflammation
Complement Proteins
Inflammasome
ROS Production
Apoptosis
Cell Cycle
Immunoproteasome
Proteasome
Unfolded Protein
Glycolysis
Pentose Phosphate
TCA Cycle
OXPHOS
FAAO
FABO
AA Metabolism
Peroxisome
CTL
PSO

Supplemental Figure 2: Analysis of cellular and molecular pathway signatures in lesional PSO shows increased expression of keratinocyte cell signatures as well as TNF and Th17 pathway gene signatures. GSVA enrichment scores of (**A**) cellular gene signatures and (**B**) pathway gene signatures in three datasets including PSO samples (blue) and control samples (grey). The number of PSO samples per dataset that lie -1 standard deviation of the mean of the control samples is denoted on the first subtext line. The number of PSO samples per dataset that lie +1 standard deviation of the mean of the control samples is denoted on the second subtext line. Welch's t-test: * $p < 0.05$; ** $p < 0.01$; *** $p < 0.001$; **** $p < 0.0001$.

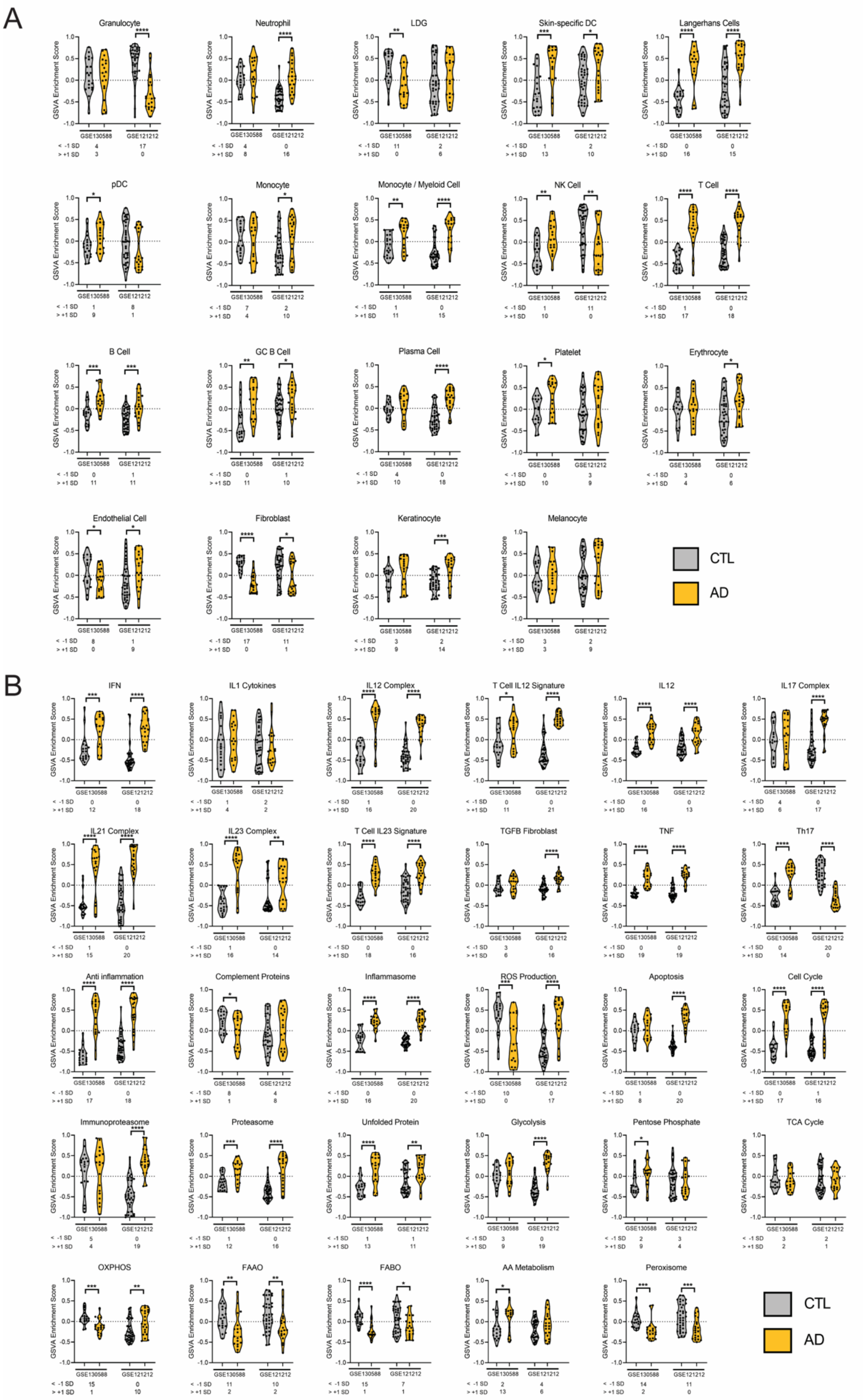
A
Granulocyte
Neutrophil
LDG
Skin-specific DC
Langerhans Cells
pDC
Monocyte
Monocyte / Myeloid Cell
NK Cell
T Cell
B Cell
GC B Cell
Plasma Cell
Platelet
Erythrocyte
Endothelial Cell
Fibroblast
Keratinocyte
Melanocyte
GSVA Enrichment Score
GSE130588
GSE121212
< -1 SD
> +1 SD
CTL
AD
B
IFN
IL1 Cytokines
IL12 Complex
T Cell IL12 Signature
IL12
IL17 Complex
IL21 Complex
IL23 Complex
T Cell IL23 Signature
TGFB Fibroblast
TNF
Th17
Anti inflammation
Complement Proteins
Inflammasome
ROS Production
Apoptosis
Cell Cycle
Immunoproteasome
Proteasome
Unfolded Protein
Glycolysis
Pentose Phosphate
TCA Cycle
OXPHOS
FAAO
FABO
AA Metabolism
Peroxisome
CTL
AD

Supplemental Figure 3: Analysis of cellular and molecular pathway signatures in lesional AD shows increased expression of skin-specific dendritic cell, B cell and IL12 inflammatory pathway gene signatures. GSVA enrichment scores of (**A**) cellular gene signatures and (**B**) pathway gene signatures in two datasets including AD samples (yellow) and control samples (grey). The number of AD samples per dataset that lie -1 standard deviation of the mean of the control samples is denoted on the first subtext line. The number of AD samples per dataset that lie +1 standard deviation of the mean of the control samples is denoted on the second subtext line. Welch's t-test: * $p < 0.05$; ** $p < 0.01$; *** $p < 0.001$; **** $p < 0.0001$.

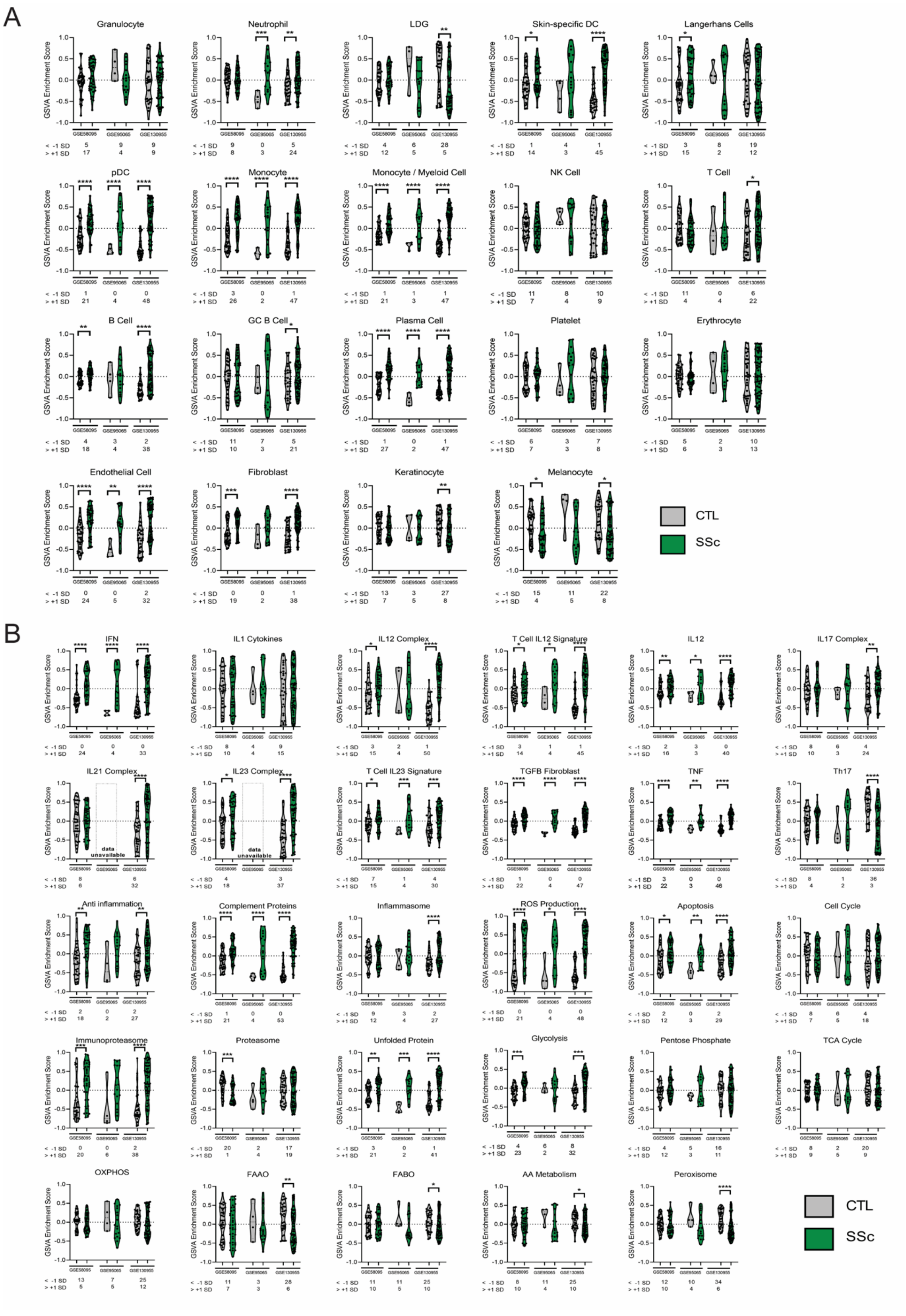
A
Granulocyte
Neutrophil
LDG
Skin-specific DC
Langerhans Cells
pDC
Monocyte
Monocyte / Myeloid Cell
NK Cell
T Cell
B Cell
GC B Cell
Plasma Cell
Platelet
Erythrocyte
Endothelial Cell
Fibroblast
Keratinocyte
Melanocyte
GSVA Enrichment Score
GSE58095
GSE95065
GSE130955
< -1 SD
> +1 SD
CTL
SSc
B
IFN
IL1 Cytokines
IL12 Complex
T Cell IL12 Signature
IL12
IL17 Complex
IL21 Complex
IL23 Complex
T Cell IL23 Signature
TGFB Fibroblast
TNF
Th17
data unavailable
Anti inflammation
Complement Proteins
Inflammasome
ROS Production
Apoptosis
Cell Cycle
Immunoproteasome
Proteasome
Unfolded Protein
Glycolysis
Pentose Phosphate
TCA Cycle
OXPHOS
FAAO
FABO
AA Metabolism
Peroxisome
CTL
SSc

Supplemental Figure 4: Analysis of cellular and molecular pathway signatures in lesional SSc samples shows increased expression of myeloid-specific cell and TGFβ fibroblast gene signatures. GSVA enrichment scores of (**A**) cellular gene signatures and (**B**) pathway gene signatures in three datasets including SSc samples (green) and control samples (grey). The number of SSc samples per dataset that lie -1 standard deviation of the mean of the control samples is denoted on the first subtext line. The number of SSc samples per dataset that lie +1 standard deviation of the mean of the control samples is denoted on the second subtext line. Welch's t-test: * $p < 0.05$; ** $p < 0.01$; *** $p < 0.001$; **** $p < 0.0001$.

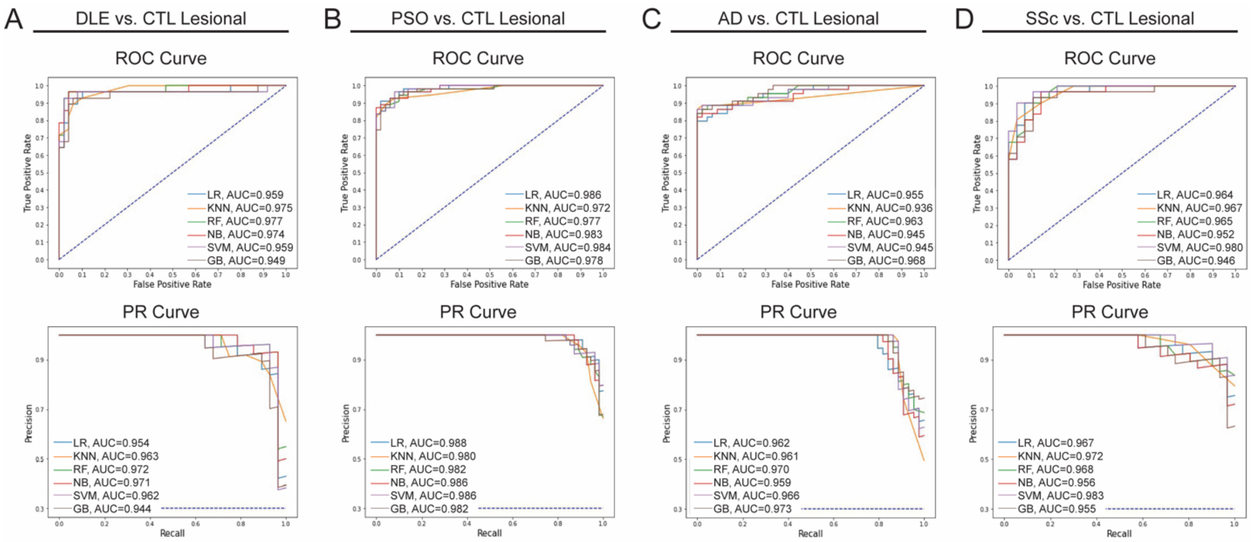

E

Disease vs. Control Lesional Results

Comparison		Classifier	Sensitivity	Specificity	Cohen's kappa	Precision	f-1 score	Accuracy
CTL	DLE	Logistic R	0.89	0.94	0.83	0.89	0.89	0.92
	PSO		0.96	0.90	0.87	0.91	0.93	0.93
	AD		0.84	0.89	0.73	0.88	0.85	0.87
	SSc		0.90	0.89	0.80	0.90	0.90	0.90
	DLE	Random F	0.93	0.98	0.92	0.96	0.94	0.96
	PSO		0.95	0.88	0.82	0.90	0.92	0.91
	AD		0.84	0.98	0.82	0.97	0.90	0.91
	SSc		0.87	0.89	0.76	0.90	0.88	0.88
	DLE	SVM	0.89	0.98	0.88	0.96	0.92	0.95
	PSO		0.93	0.92	0.84	0.93	0.93	0.92
	AD		0.86	0.99	0.86	0.99	0.92	0.93
	SSc		0.90	0.93	0.83	0.93	0.91	0.92
	DLE	Naïve Bayes	0.96	0.94	0.88	0.90	0.92	0.95
	PSO		0.89	0.94	0.82	0.94	0.91	0.91
	AD		0.86	0.91	0.77	0.90	0.88	0.89
	SSc		0.84	0.86	0.69	0.87	0.85	0.85
	DLE	KNN	0.82	0.96	0.79	0.92	0.86	0.91
	PSO		0.89	0.96	0.84	0.96	0.92	0.92
	AD		0.86	0.99	0.86	0.99	0.92	0.93
	SSc		0.81	0.96	0.76	0.96	0.87	0.88
	DLE	GB	0.86	0.96	0.83	0.92	0.89	0.92
	PSO		0.95	0.88	0.83	0.90	0.92	0.91
	AD		0.86	0.98	0.84	0.97	0.91	0.92
	SSc		0.84	0.89	0.73	0.90	0.87	0.86

Supplemental Figure 5: ML accurately classifies lesional skin and control skin samples. ROC curve and PR curve of all ML algorithms to separate lesional samples from healthy control samples using all cellular and pathway gene signatures/ features. ML classifiers include: logistic regression (LR, blue), k-nearest neighbors (KNN, orange), random forest (RF, green), naïve Bayes (NB, red), support vector machine (SVM, purple) and gradient boosting (GB, brown). (**A**) DLE versus control; (**B**) PSO versus control; (**C**) AD versus control; and (**D**) SSc versus control. (**E**) Classification metrics including sensitivity, specificity, Cohen's kappa score, precision, f-1 score and accuracy to properly separate lesional disease samples (DLE, PSO, AD or SSc) from healthy control samples with each ML classifier. Refer to **tables S3A-B** for details about ML. Collinear features were removed (**fig. S6**)

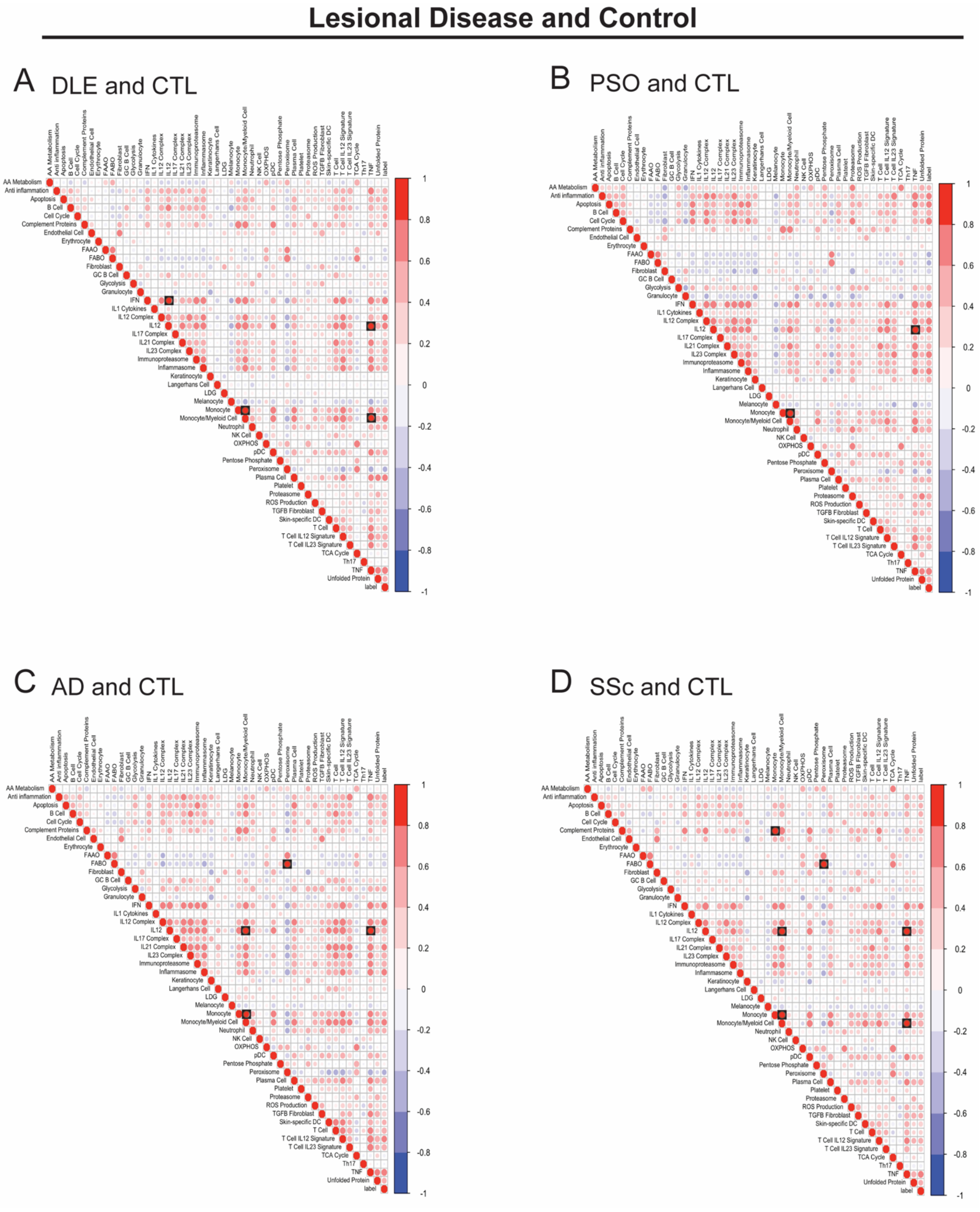
Lesional Disease and Control
A DLE and CTL
B PSO and CTL
C AD and CTL
D SSc and CTL
1
0.8
0.6
0.4
0.2
0
-0.2
-0.4
-0.6
-0.8
-1

Supplemental Figure 6: Analysis of correlated features from cellular and pathway signatures was used to extract collinear features for lesional ML binary classifications. Correlation plots of GSVA enrichment scores of pooled control samples and pooled lesional (**A**) DLE, (**B**) PSO, (**C**) AD and (**D**) SSc samples. Black boxes indicate collinear samples with Pearson correlation coefficient greater than 0.8, then the feature with the lower correlation was removed using a greedy elimination approach.

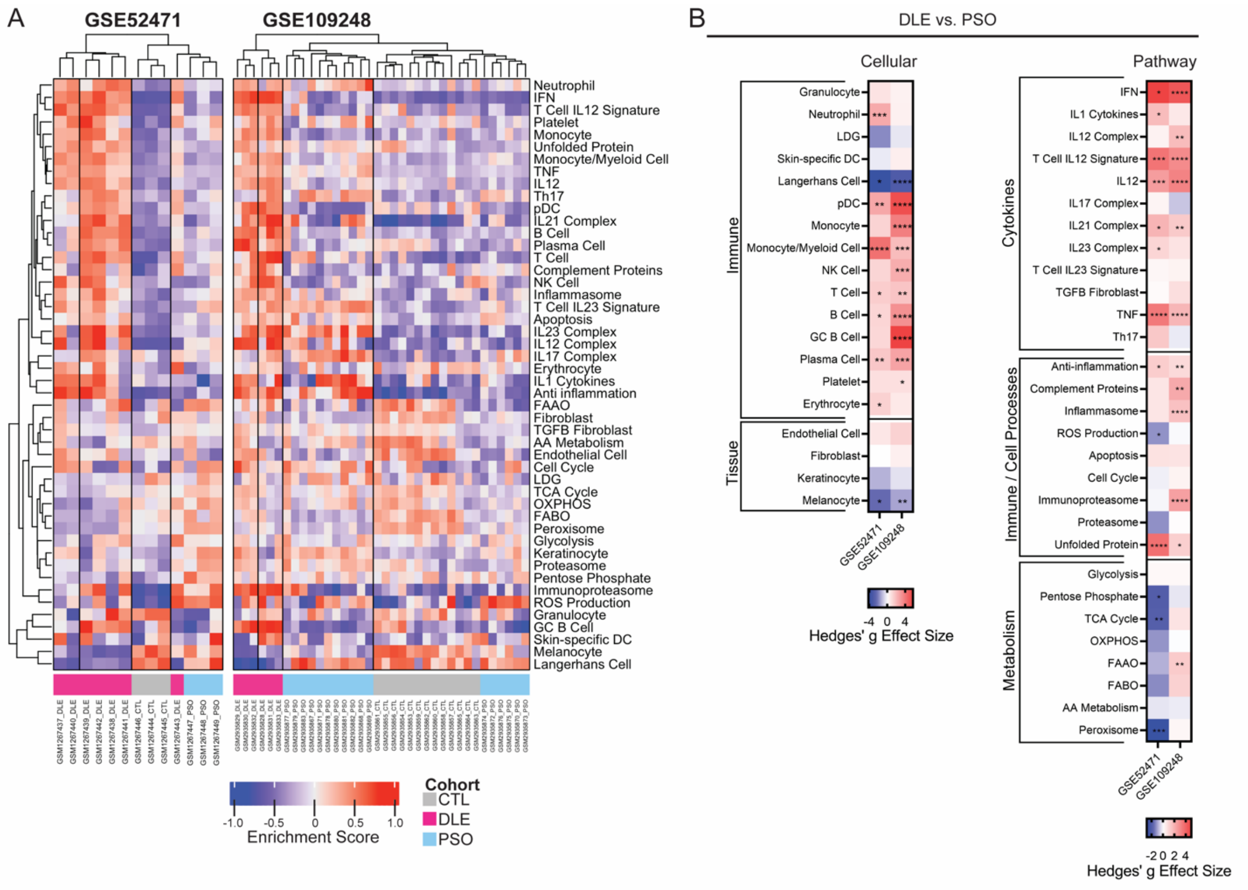

A
GSE52471
GSE109248
Neutrophil
IFN
T Cell IL12 Signature
Platelet
Monocyte
Unfolded Protein
Monocyte/Myeloid Cell
TNF
IL12
Th17
pDC
IL21 Complex
B Cell
Plasma Cell
T Cell
Complement Proteins
NK Cell
Inflammasome
T Cell IL23 Signature
Apoptosis
IL23 Complex
IL12 Complex
IL17 Complex
Erythrocyte
IL1 Cytokines
Anti inflammation
FAAO
Fibroblast
TGFB Fibroblast
AA Metabolism
Endothelial Cell
Cell Cycle
LDG
TCA Cycle
OXPHOS
FABO
Peroxisome
Glycolysis
Keratinocyte
Proteasome
Pentose Phosphate
Immunoproteasome
ROS Production
Granulocyte
GC B Cell
Skin-specific DC
Melanocyte
Langerhans Cell
-1.0 -0.5 0 0.5 1.0
Enrichment Score
Cohort
CTL
DLE
PSO
B
DLE vs. PSO
Cellular
Immune
Tissue
Granulocyte
Neutrophil
LDG
Skin-specific DC
Langerhans Cell
pDC
Monocyte
Monocyte/Myeloid Cell
NK Cell
T Cell
B Cell
GC B Cell
Plasma Cell
Platelet
Erythrocyte
Endothelial Cell
Fibroblast
Keratinocyte
Melanocyte
GSE52471
GSE109248
-4 0 4
Hedges' g Effect Size
Pathway
Cytokines
IFN
IL1 Cytokines
IL12 Complex
T Cell IL12 Signature
IL12
IL17 Complex
IL21 Complex
IL23 Complex
T Cell IL23 Signature
TGFB Fibroblast
TNF
Th17
Immune / Cell Processes
Anti-inflammation
Complement Proteins
Inflammasome
ROS Production
Apoptosis
Cell Cycle
Immunoproteasome
Proteasome
Unfolded Protein
Metabolism
Glycolysis
Pentose Phosphate
TCA Cycle
OXPHOS
FAAO
FABO
AA Metabolism
Peroxisome
GSE52471
GSE109248
-2 0 2 4
Hedges' g Effect Size

Supplemental Figure 7: **Direct comparison of DLE and PSO samples using GSVA shows key differences in enrichment of inflammatory cell and pathway signatures.** (**A**) Hierarchical clustering (k=4 clusters) of GSVA enrichments scores of cellular and pathway gene signatures in two datasets that included DLE, PSO and healthy control samples. (**B**) Heatmap of GSVA enrichment scores of DLE compared to PSO samples in two datasets of cellular (left) and pathway (right) gene signatures. Heatmap visualization uses red (enriched signature, >0) and blue (decreased signature, <0). Welch's t-test: * $p < 0.05$; ** $p < 0.01$; *** $p < 0.001$; **** $p < 0.0001$.

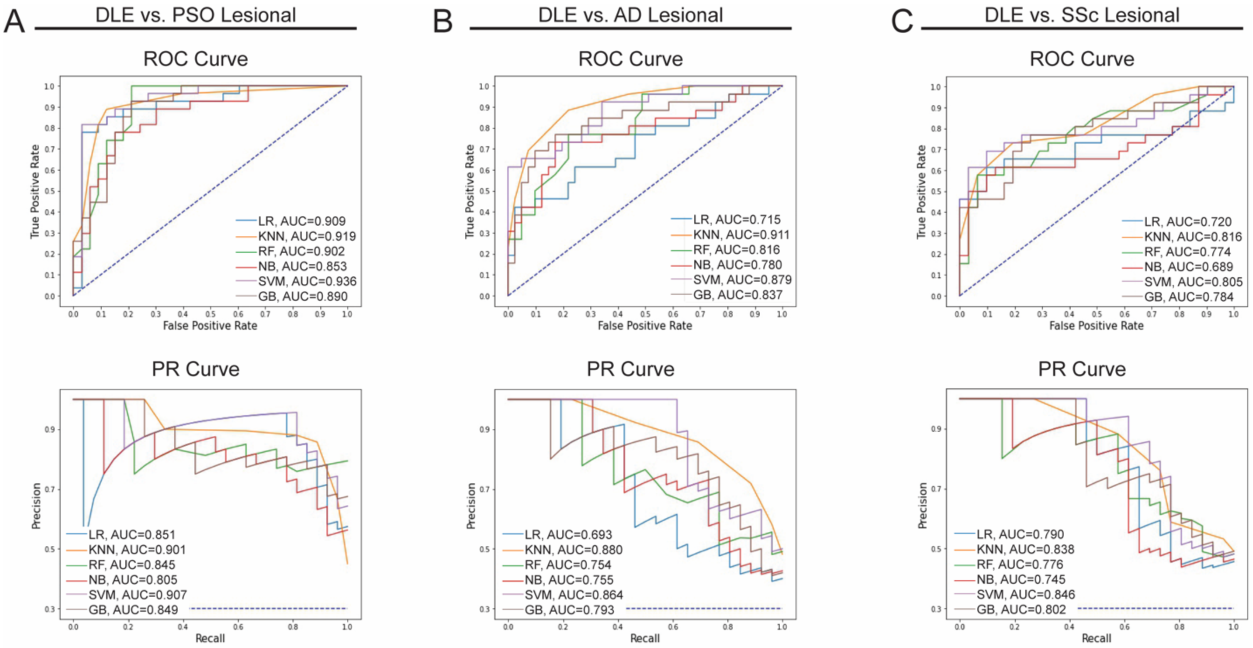

D

DLE vs. Disease Lesional Results

Comparison		Classifier	Sensitivity	Specificity	Cohen's kappa	Precision	f-1 score	Accuracy
DLE	PSO	Logistic R	0.78	0.91	0.69	0.88	0.83	0.85
	AD		0.50	0.78	0.29	0.59	0.54	0.67
	SSc		0.65	0.77	0.43	0.71	0.68	0.72
	PSO	Random F	0.63	0.88	0.52	0.81	0.71	0.77
	AD		0.42	0.90	0.35	0.73	0.53	0.72
	SSc		0.62	0.84	0.46	0.76	0.68	0.74
	PSO	SVM	0.81	0.97	0.80	0.96	0.88	0.90
	AD		0.65	0.93	0.61	0.85	0.74	0.82
	SSc		0.69	0.90	0.60	0.86	0.77	0.81
	PSO	Naïve Bayes	0.67	0.85	0.52	0.78	0.72	0.77
	AD		0.65	0.83	0.49	0.71	0.68	0.76
	SSc		0.62	0.81	0.43	0.73	0.67	0.72
	PSO	KNN	0.63	0.94	0.58	0.89	0.74	0.80
	AD		0.69	0.93	0.64	0.86	0.77	0.84
	SSc		0.73	0.81	0.54	0.76	0.74	0.77
	PSO	GB	0.70	0.85	0.56	0.79	0.74	0.78
	AD		0.69	0.85	0.55	0.75	0.72	0.79
	SSc		0.69	0.81	0.50	0.75	0.72	0.75

Supplemental Figure 8: ML accurately classifies lesional DLE from lesional PSO, AD and SSc. ROC curve and PR curve of all ML algorithms to separate lesional DLE from other inflammatory skin diseases using all cellular and pathway gene signatures/ features. ML classifiers include: logistic regression (LR, blue), k-nearest neighbors (KNN, orange), random forest (RF, green), naïve Bayes (NB, red), support vector machine (SVM, purple) and gradient boosting (GB, brown). (**A**) DLE versus PSO; (**B**) DLE versus AD; and (**C**) DLE versus SSc. (**D**) Classification metrics including sensitivity, specificity, Cohen's kappa score, precision, f-1 score and accuracy to properly separate lesional DLE samples from lesional PSO, AD, and SSc samples with each ML classifier. Refer to **tables S3A-B** for details about ML. Collinear features were removed (**fig. S9**).

Lesional DLE and Lesional Disease

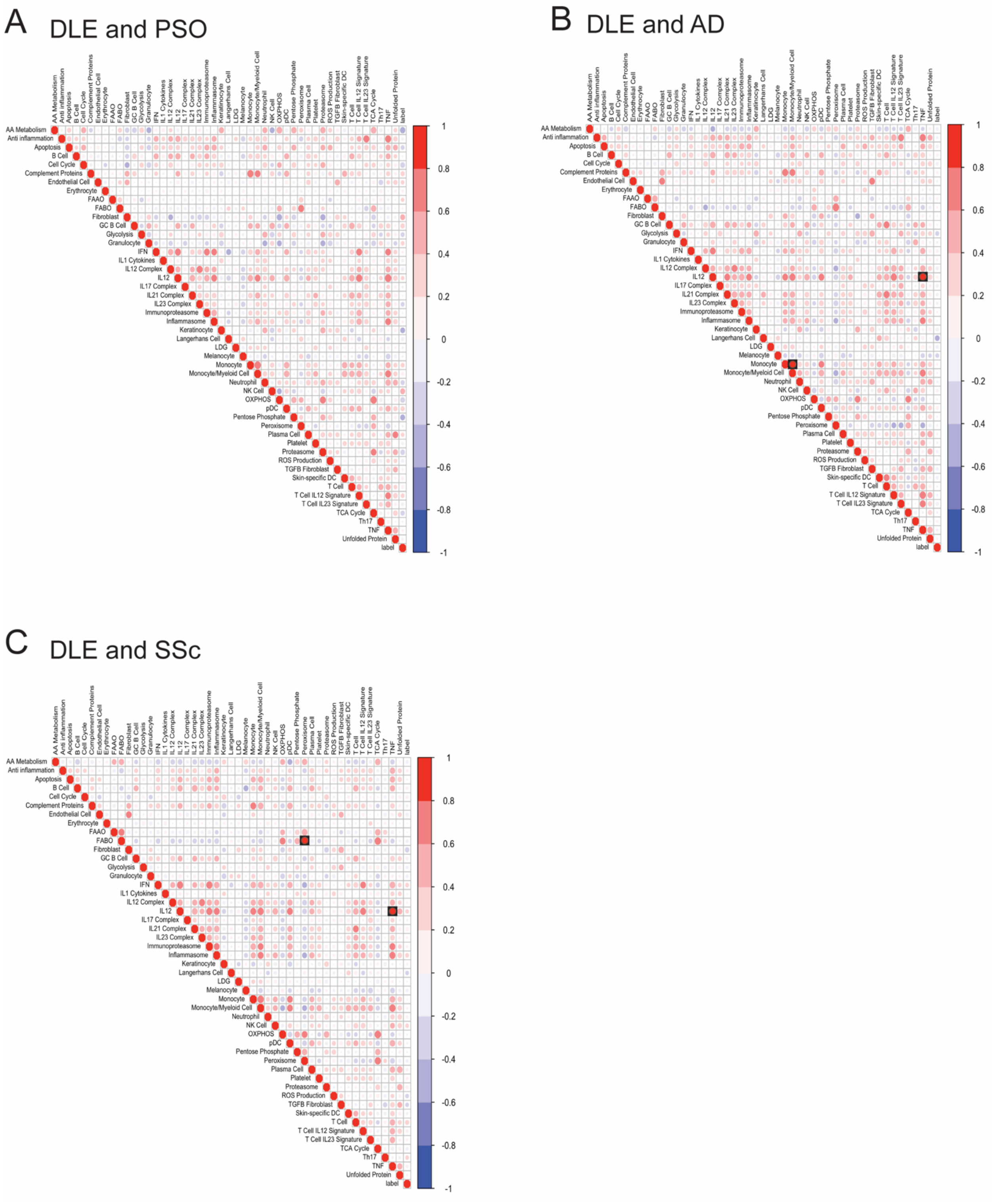

Supplemental Figure 9: Analysis of correlated features from cellular and pathway signatures was used to extract collinear features for lesional ML binary classifications compared to DLE. Correlation plots of GSVA enrichment scores of lesional DLE and lesional (**A**) PSO, (**B**) AD and (**C**) SSc samples. Correlations outlined in black were reduced to only include one feature. Black boxes indicate collinear samples with Pearson correlation coefficient greater than 0.8, then the feature with the lower correlation was removed using a greedy elimination approach.

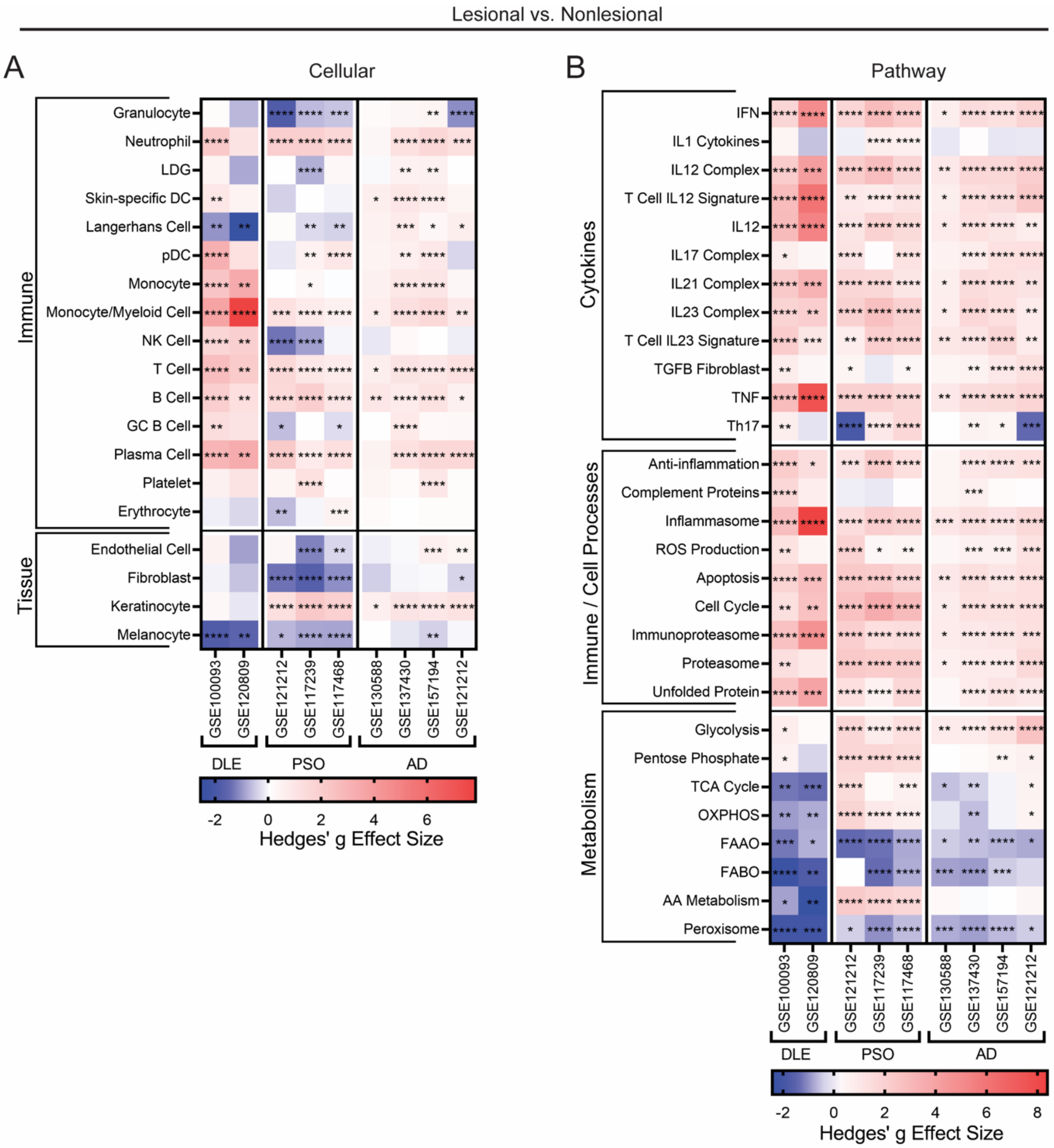

Lesional vs. Nonlesional
A
Cellular
Immune
Granulocyte
Neutrophil
LDG
Skin-specific DC
Langerhans Cell
pDC
Monocyte
Monocyte/Myeloid Cell
NK Cell
T Cell
B Cell
GC B Cell
Plasma Cell
Platelet
Erythrocyte
Tissue
Endothelial Cell
Fibroblast
Keratinocyte
Melanocyte
GSE100093
GSE120809
GSE121212
GSE117239
GSE117468
GSE130588
GSE137430
GSE157194
GSE121212
DLE
PSO
AD
-2
0
2
4
6
Hedges' g Effect Size
B
Pathway
Cytokines
IFN
IL1 Cytokines
IL12 Complex
T Cell IL12 Signature
IL12
IL17 Complex
IL21 Complex
IL23 Complex
T Cell IL23 Signature
TGFB Fibroblast
TNF
Th17
Immune / Cell Processes
Anti-inflammation
Complement Proteins
Inflammasome
ROS Production
Apoptosis
Cell Cycle
Immunoproteasome
Proteasome
Unfolded Protein
Metabolism
Glycolysis
Pentose Phosphate
TCA Cycle
OXPHOS
FAAO
FABO
AA Metabolism
Peroxisome
GSE100093
GSE120809
GSE121212
GSE117239
GSE117468
GSE130588
GSE137430
GSE157194
GSE121212
DLE
PSO
AD
-2
0
2
4
6
8
Hedges' g Effect Size

Supplemental Figure 10: GSVA enrichment of lesional skin compared to nonlesional skin. Hedges' g effect sizes of GSVA enrichment scores for paired lesional and nonlesional samples, including two DLE, four AD and three PSO datasets using (**A**) cellular gene signatures and (**B**) pathway gene signatures. Lesional samples were compared to their respective nonlesional paired samples in DLE, AD and PSO. Heatmap visualization uses red (enriched signature, >0) and blue (decreased signature, <0). Paired t-test: * $p < 0.05$; ** $p < 0.01$; *** $p < 0.001$; **** $p < 0.0001$.

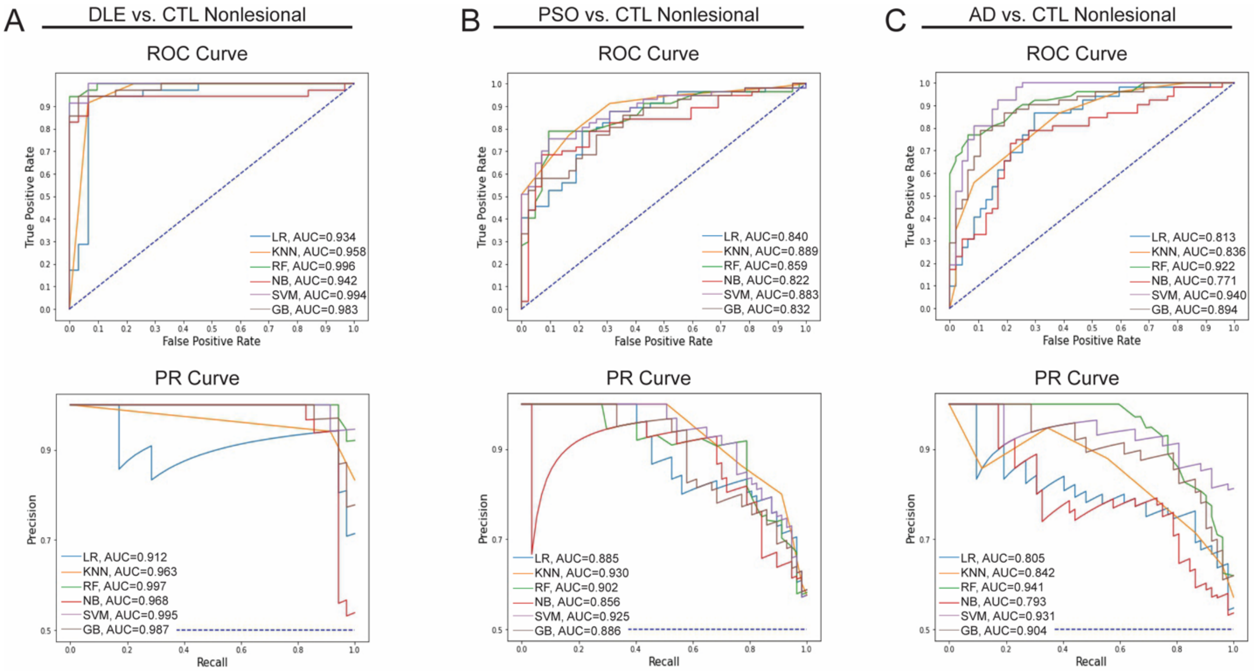

D

Disease vs. Control Nonlesional Results									
**Comparison**		**Classifier**	**Sensitivity**	**Specificity**	**Cohen's kappa**	**Precision**	**f-1 score**	**Accuracy**
CTL	DLE	Logistic R	0.94	0.94	0.88	0.94	0.94	0.94
	PSO		0.72	0.79	0.49	0.82	0.77	0.75
	AD		0.63	0.81	0.44	0.79	0.70	0.72
	DLE	Random F	0.94	0.97	0.91	0.97	0.95	0.95
	PSO		0.75	0.90	0.64	0.91	0.82	0.82
	AD		0.75	0.94	0.68	0.93	0.83	0.84
	DLE	SVM	0.94	0.94	0.88	0.94	0.94	0.94
	PSO		0.75	0.90	0.64	0.91	0.82	0.82
	AD		0.83	0.85	0.68	0.86	0.84	0.84
	DLE	Naïve Bayes	0.91	0.94	0.85	0.94	0.92	0.92
	PSO		0.77	0.76	0.53	0.81	0.79	0.77
	AD		0.58	0.83	0.40	0.79	0.67	0.70
	DLE	KNN	0.99	0.61	0.63	0.74	0.85	0.82
	PSO		0.77	0.83	0.59	0.86	0.81	0.80
	AD		0.56	0.91	0.46	0.88	0.68	0.73
	DLE	GB	0.94	0.9	0.85	0.92	0.93	0.92
	PSO		0.68	0.79	0.46	0.81	0.74	0.73
	AD		0.79	0.85	0.64	0.85	0.82	0.82

Supplemental Figure 11: ML accurately classifies nonlesional skin and control skin samples. ROC curve and PR curve of all machine learning classification algorithms to separate nonlesional samples from healthy control samples using all cellular and pathway gene signatures/ features. ML classifiers include: logistic regression (LR, blue), k-nearest neighbors (KNN, orange), random forest (RF, green), naïve Bayes (NB, red), support vector machine (SVM, purple) and gradient boosting (GB, brown). (**A**) DLE versus control; (**B**) PSO versus control; and (**C**) AD versus control. (**D**) Classification metrics including sensitivity, specificity, Cohen's kappa score, precision, f-1 score and accuracy to properly separate nonlesional disease samples (DLE, PSO or AD) from healthy control samples with each ML classifier. Refer to **tables S3A-B** for details about ML. Collinear features were removed (**fig. S12**).

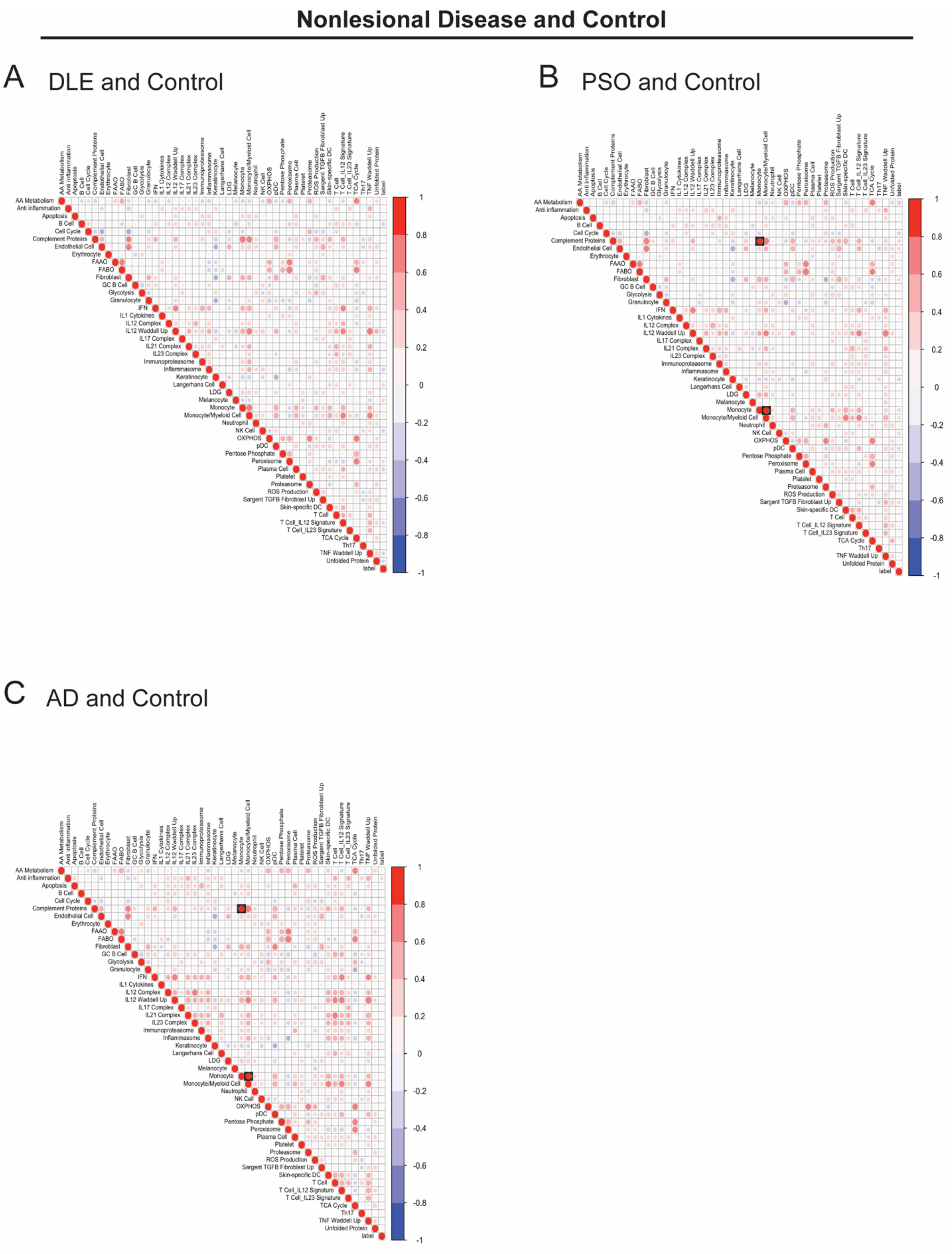

Nonlesional Disease and Control
A
DLE and Control
B
PSO and Control
C
AD and Control
1
0.8
0.6
0.4
0.2
0
-0.2
-0.4
-0.6
-0.8
-1

Supplemental Figure 12: Analysis of correlated features from cellular and pathway signatures was used to extract collinear features for nonlesional ML binary classification. Correlation plots of GSVA enrichment scores of control samples and nonlesional (**A**) DLE, (**B**) PSO and (**C**) AD samples. Correlations outlined in black were reduced to only include one feature. Black boxes indicate collinear samples with Pearson correlation coefficient greater than 0.8, then the feature with the lower correlation was removed using a greedy elimination approach.

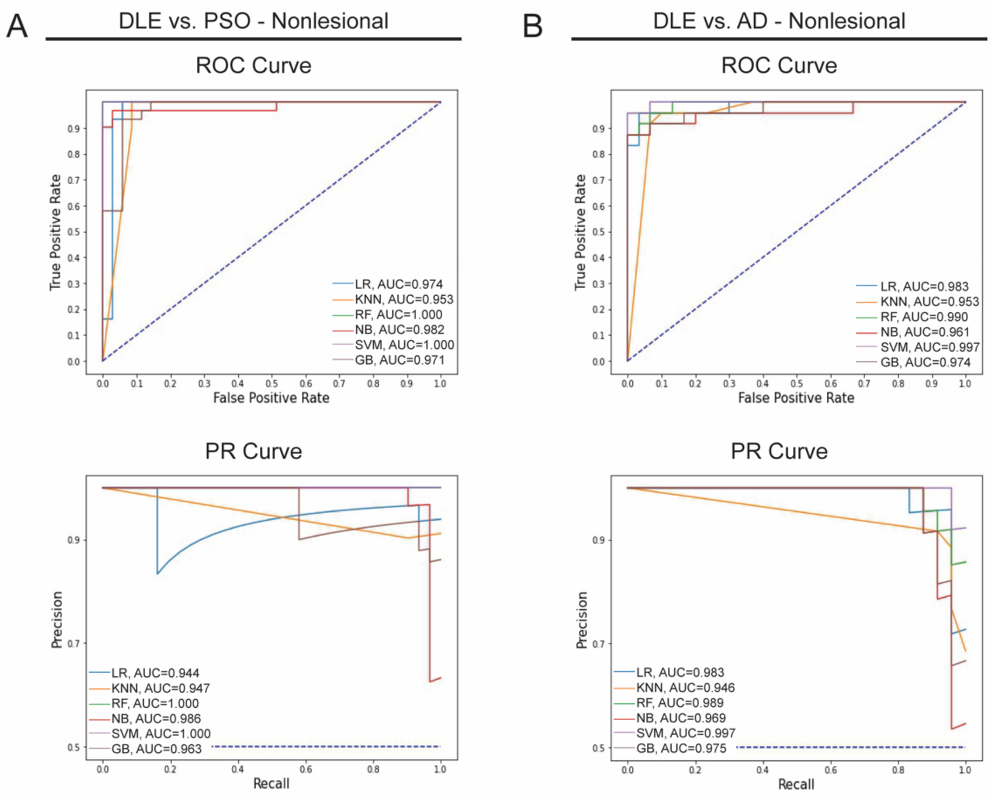

C

DLE vs. Disease Nonlesional Results									
Comparison		Classifier	Sensitivity	Specificity	Cohen's kappa	Precision	f-1 score	Accuracy
DLE	PSO	Logistitc R	0.97	0.94	0.91	0.94	0.95	0.95
	AD		0.92	0.97	0.89	0.96	0.94	0.94
	PSO	Random F	0.99	0.94	0.94	0.94	0.97	0.97
	AD		0.88	0.97	0.85	0.95	0.91	0.93
	PSO	SVM	0.99	0.97	0.97	0.97	0.98	0.98
	AD		0.96	0.99	0.96	0.99	0.98	0.98
	PSO	Naïve Bayes	0.9	0.97	0.88	0.97	0.93	0.94
	AD		0.92	0.93	0.85	0.92	0.92	0.93
	PSO	KNN	0.99	0.83	0.82	0.84	0.91	0.91
	AD		0.96	0.8	0.74	0.79	0.87	0.87
	PSO	GB	0.94	0.91	0.85	0.91	0.92	0.92
	AD		0.88	0.93	0.81	0.91	0.89	0.91

Supplemental Figure 13: **ML distinguishes nonlesional DLE from nonlesional PSO and nonlesional AD.** ROC curve and PR curve of all machine learning classification algorithms to separate nonlesional DLE from other inflammatory skin diseases using all cellular and pathway gene signatures/ features. ML classifiers include: logistic regression (LR, blue), k-nearest neighbors (KNN, orange), random forest (RF, green), naïve Bayes (NB, red), support vector machine (SVM, purple) and gradient boosting (GB, brown). (**A**) DLE versus PSO and (**B**) DLE versus AD. (**C**) Classification metrics including sensitivity, specificity, Cohen's kappa score, precision, f-1 score and accuracy to properly separate nonlesional DLE samples from nonlesional PSO and nonlesional AD samples with each ML classifier. Refer to **tables S3A-B** for details about ML. Collinear features were removed (**fig. S15**).

A

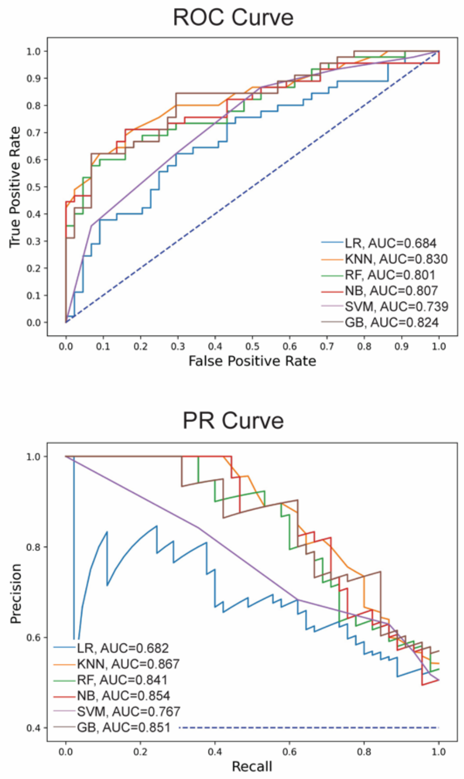

B

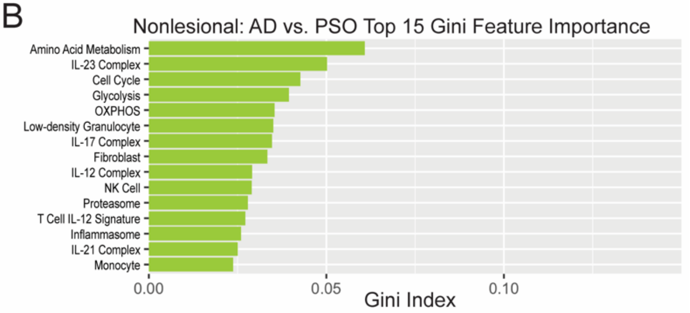

C

NL PSO vs. NL AD						
Classifier	Sensitivity	Specificity	Cohen's kappa	Precision	f-1 score	Accuracy
Logistic R	0.78	0.48	0.26	0.6	0.68	0.63
Random F	0.80	0.64	0.44	0.69	0.74	0.72
SVM	0.78	0.57	0.35	0.65	0.71	0.67
Naïve Bayes	0.78	0.57	0.35	0.65	0.71	0.67
KNN	0.87	0.48	0.35	0.63	0.73	0.67
GB	0.84	0.61	0.46	0.69	0.76	0.73

D

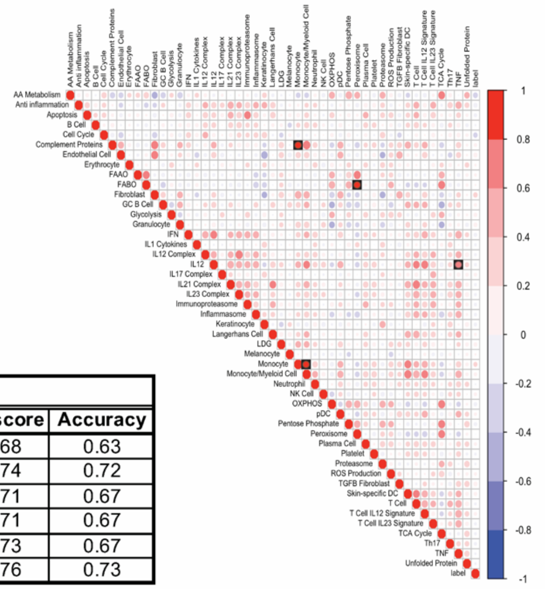

Supplemental Figure 14: **ML less effectively classifies nonlesional PSO from nonlesional AD.** (**A**) ROC curve and PR curve of all ML classification algorithms to separate nonlesional PSO from nonlesional AD samples using all cellular and pathway gene signatures/ features. ML classifiers include: logistic regression (LR, blue), k-nearest neighbors (KNN, orange), random forest (RF, green), naïve Bayes (NB, red), support vector machine (SVM, purple) and gradient boosting (GB, brown). (**B**) Top 15 features important in classifying nonlesional PSO from nonlesional AD using Gini feature importance. (**C**) Classification metrics including sensitivity, specificity, Cohen's kappa score, precision, f-1 score and accuracy to properly separate nonlesional PSO samples from nonlesional AD samples with each ML classifier. (**D**) Correlation plots of GSVA enrichment scores of nonlesional PSO and nonlesional AD samples. Black boxes indicate collinear samples with Pearson correlation coefficient greater than 0.8, then the feature with the lower correlation was removed using a greedy elimination approach.

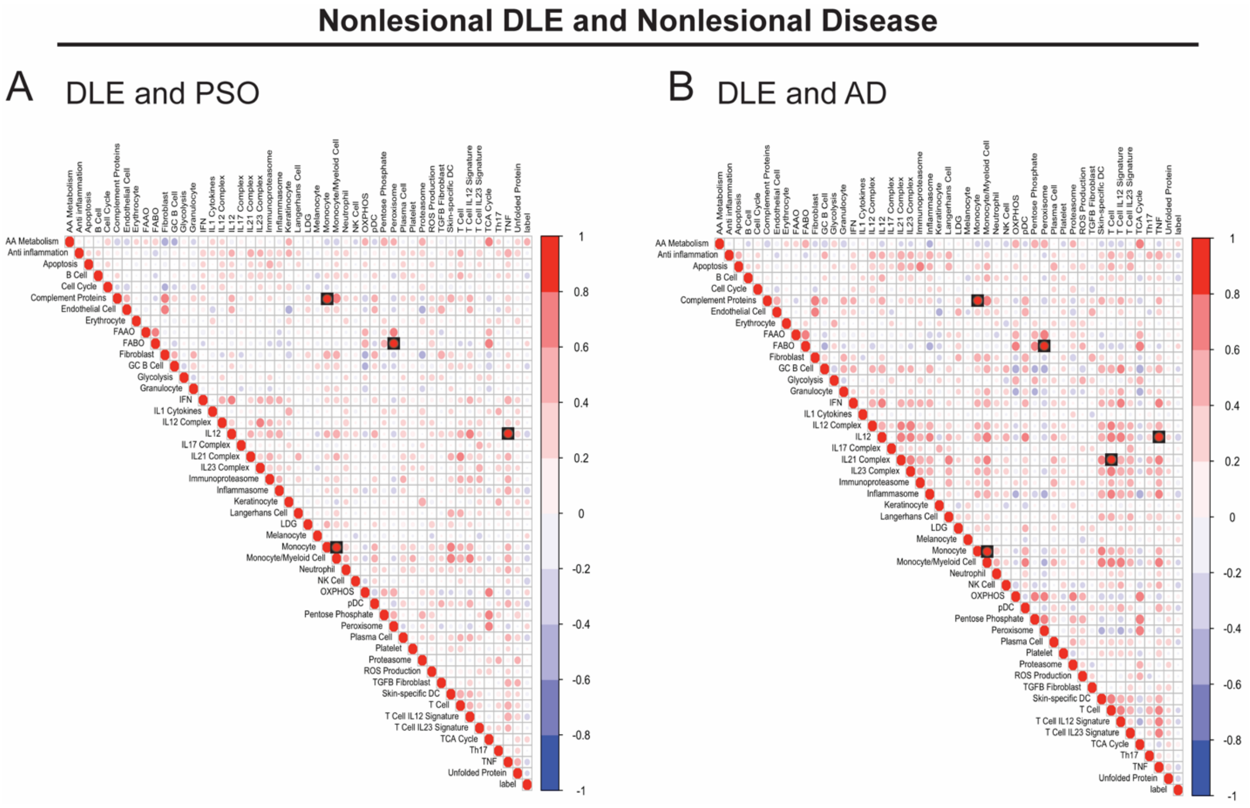

Nonlesional DLE and Nonlesional Disease
A
DLE and PSO
B
DLE and AD
AA Metabolism
Anti inflammation
Apoptosis
B Cell
Cell Cycle
Complement Proteins
Endothelial Cell
Erythrocyte
FAAO
FABO
Fibroblast
GC B Cell
Glycolysis
Granulocyte
IFN
IL1 Cytokines
IL12 Complex
IL12
IL17 Complex
IL21 Complex
IL23 Complex
Immunoproteasome
Inflammasome
Keratinocyte
Langerhans Cell
LDG
Melanocyte
Monocyte
Monocyte/Myeloid Cell
Neutrophil
NK Cell
OXPHOS
pDC
Pentose Phosphate
Peroxisome
Plasma Cell
Platelet
Proteasome
ROS Production
TGFB Fibroblast
Skin-specific DC
T Cell
T Cell IL12 Signature
T Cell IL23 Signature
TCA Cycle
Th17
TNF
Unfolded Protein
label
1
0.8
0.6
0.4
0.2
0
-0.2
-0.4
-0.6
-0.8
-1

Supplemental Figure 15: **Analysis of correlated features from cellular and pathway signatures was used to extract collinear features for nonlesional ML binary classification compared to DLE.** Correlation plots of GSVA enrichment scores of nonlesional DLE and (**A**) nonlesional PSO and (**B**) nonlesional AD samples. Black boxes indicate collinear samples with Pearson correlation coefficient greater than 0.8, then the feature with the lower correlation was removed using a greedy elimination approach.

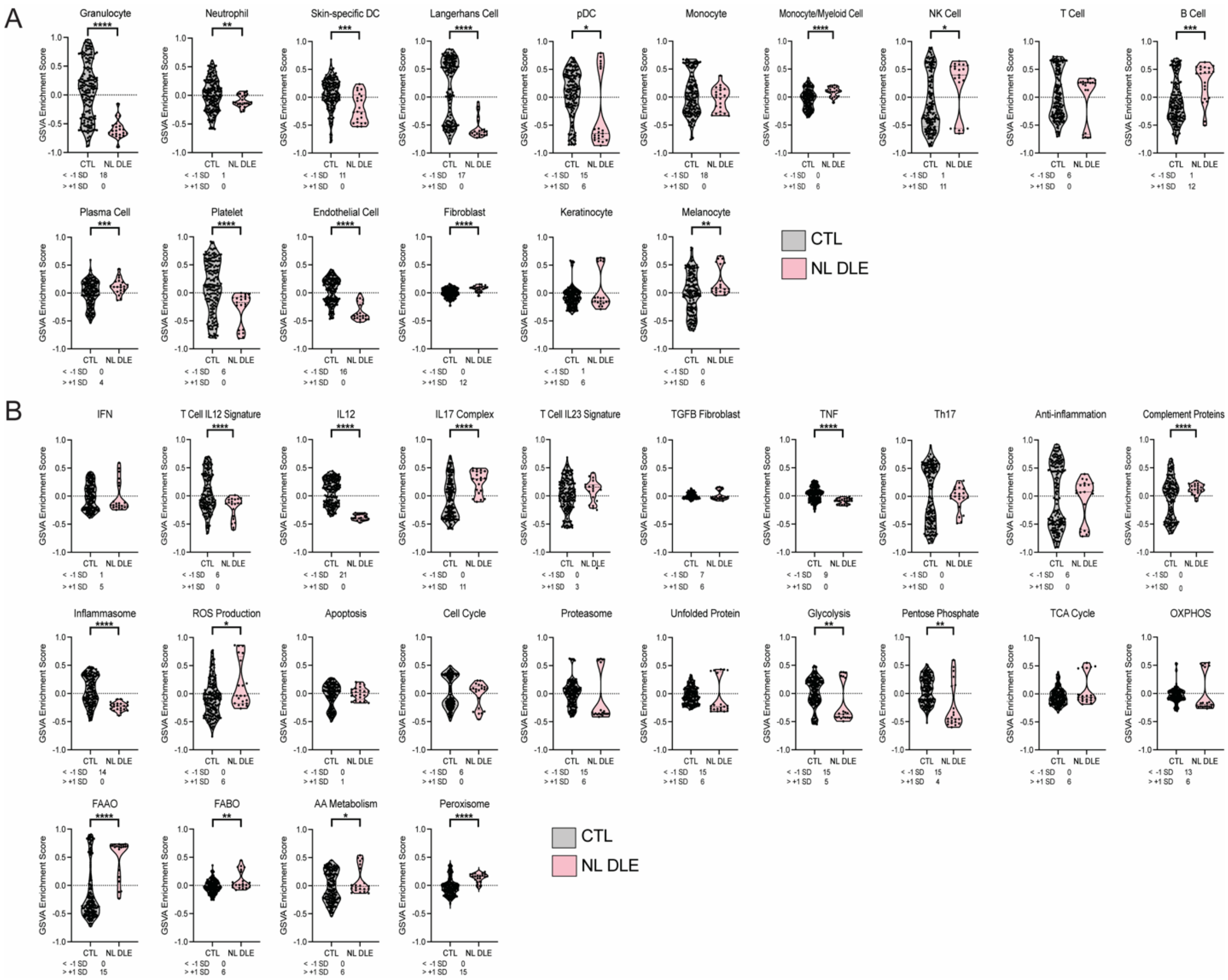
A
Granulocyte
Neutrophil
Skin-specific DC
Langerhans Cell
pDC
Monocyte
Monocyte/Myeloid Cell
NK Cell
T Cell
B Cell
Plasma Cell
Platelet
Endothelial Cell
Fibroblast
Keratinocyte
Melanocyte
GSVA Enrichment Score
CTL
NL DLE
< -1 SD
> +1 SD
B
IFN
T Cell IL12 Signature
IL12
IL17 Complex
T Cell IL23 Signature
TGFB Fibroblast
TNF
Th17
Anti-inflammation
Complement Proteins
Inflammasome
ROS Production
Apoptosis
Cell Cycle
Proteasome
Unfolded Protein
Glycolysis
Pentose Phosphate
TCA Cycle
OXPHOS
FAAO
FABO
AA Metabolism
Peroxisome

Supplemental Figure 16: Analysis of cellular and molecular pathway signatures in nonlesional DLE shows upregulation of B cell, plasma cell and fatty acid metabolism gene signatures. GSVA enrichment scores using Z-score transformation of (**A**) cellular gene signatures and (**B**) pathway gene signatures in nonlesional DLE (light pink) and control samples (grey). The number of nonlesional DLE samples per dataset that lie -1 standard deviation of the mean of the control samples is denoted on the first subtext line. The number of DLE samples per dataset that lie +1 standard deviation of the mean of the control samples is denoted on the second subtext line. Welch's t-test: * $p < 0.05$; ** $p < 0.01$; *** $p < 0.001$; **** $p < 0.0001$.

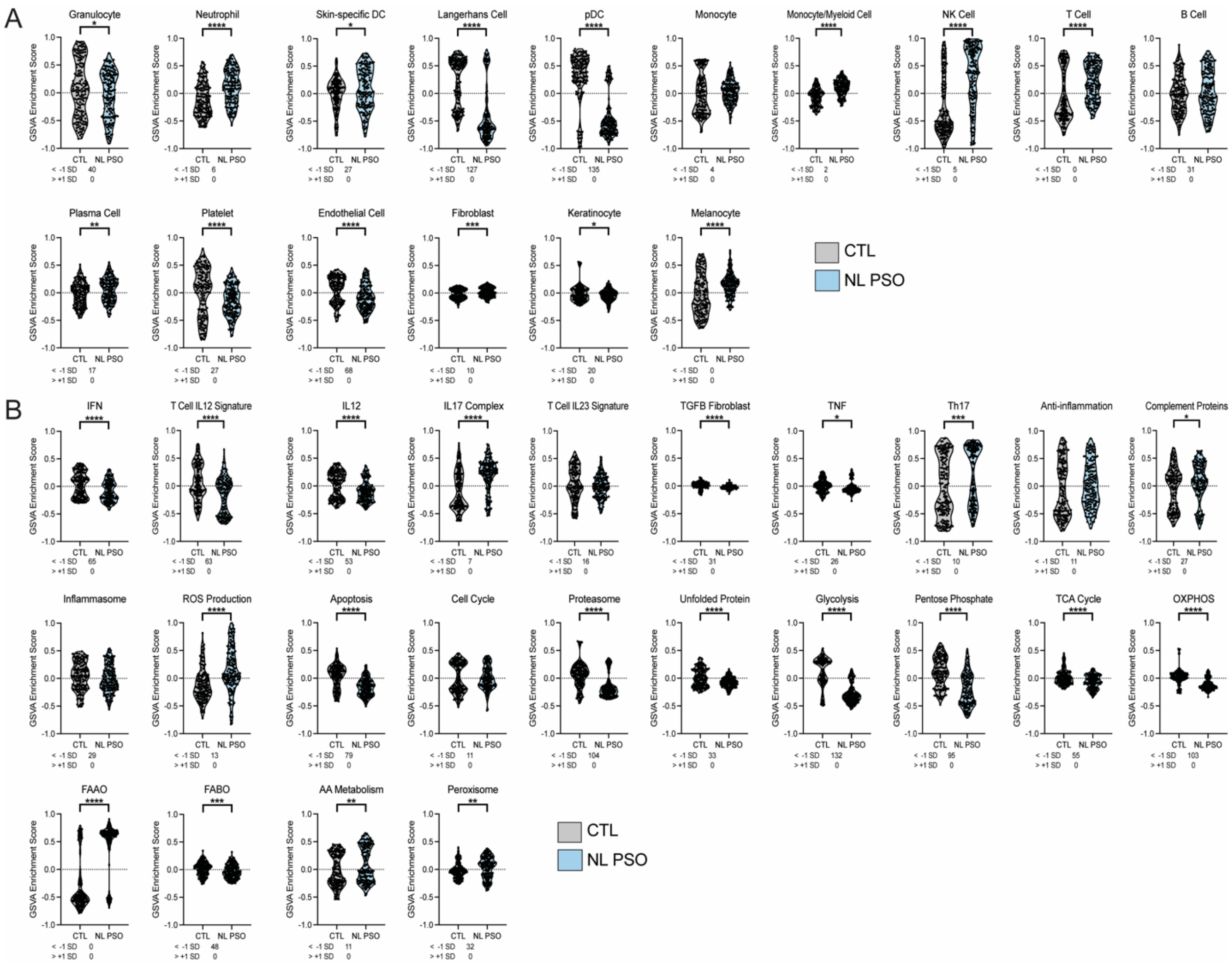
A
Granulocyte *
< -1 SD 40
> +1 SD 0
Neutrophil ****
< -1 SD 6
> +1 SD 0
Skin-specific DC *
< -1 SD 27
> +1 SD 0
Langerhans Cell ****
< -1 SD 127
> +1 SD 0
pDC ****
< -1 SD 135
> +1 SD 0
Monocyte
< -1 SD 4
> +1 SD 0
Monocyte/Myeloid Cell ****
< -1 SD 2
> +1 SD 0
NK Cell ****
< -1 SD 5
> +1 SD 0
T Cell ****
< -1 SD 0
> +1 SD 0
B Cell
< -1 SD 31
> +1 SD 0
Plasma Cell **
< -1 SD 17
> +1 SD 0
Platelet ****
< -1 SD 27
> +1 SD 0
Endothelial Cell ****
< -1 SD 68
> +1 SD 0
Fibroblast ***
< -1 SD 10
> +1 SD 0
Keratinocyte *
< -1 SD 20
> +1 SD 0
Melanocyte ****
< -1 SD 0
> +1 SD 0
CTL
NL PSO
B
IFN ****
< -1 SD 65
> +1 SD 0
T Cell IL12 Signature ****
< -1 SD 63
> +1 SD 0
IL12 ****
< -1 SD 53
> +1 SD 0
IL17 Complex ****
< -1 SD 7
> +1 SD 0
T Cell IL23 Signature
< -1 SD 16
> +1 SD 0
TGFB Fibroblast ****
< -1 SD 31
> +1 SD 0
TNF *
< -1 SD 26
> +1 SD 0
Th17 ***
< -1 SD 10
> +1 SD 0
Anti-inflammation
< -1 SD 11
> +1 SD 0
Complement Proteins *
< -1 SD 27
> +1 SD 0
Inflammasome
< -1 SD 29
> +1 SD 0
ROS Production ****
< -1 SD 13
> +1 SD 0
Apoptosis ****
< -1 SD 79
> +1 SD 0
Cell Cycle
< -1 SD 11
> +1 SD 0
Proteasome ****
< -1 SD 104
> +1 SD 0
Unfolded Protein ****
< -1 SD 33
> +1 SD 0
Glycolysis ****
< -1 SD 132
> +1 SD 0
Pentose Phosphate ****
< -1 SD 95
> +1 SD 0
TCA Cycle ****
< -1 SD 55
> +1 SD 0
OXPHOS ****
< -1 SD 103
> +1 SD 0
FAAO ****
< -1 SD 0
> +1 SD 0
FABO ***
< -1 SD 48
> +1 SD 0
AA Metabolism **
< -1 SD 11
> +1 SD 0
Peroxisome **
< -1 SD 32
> +1 SD 0
GSVA Enrichment Score
CTL
NL PSO

Supplemental Figure 17: Analysis of cellular and molecular pathway signatures in nonlesional PSO shows upregulation of innate immune cell and IL-17 gene signatures. GSVA enrichment scores using Z-score transformation of (**A**) cellular gene signatures and (**B**) pathway gene signatures in nonlesional PSO (light blue) and control samples (grey). The number of nonlesional PSO samples per dataset that lie -1 standard deviation of the mean of the control samples is denoted on the first subtext line. The number of PSO samples per dataset that lie +1 standard deviation of the mean of the control samples is denoted on the second subtext line. Welch's t-test: * $p < 0.05$; ** $p < 0.01$; *** $p < 0.001$; **** $p < 0.0001$.

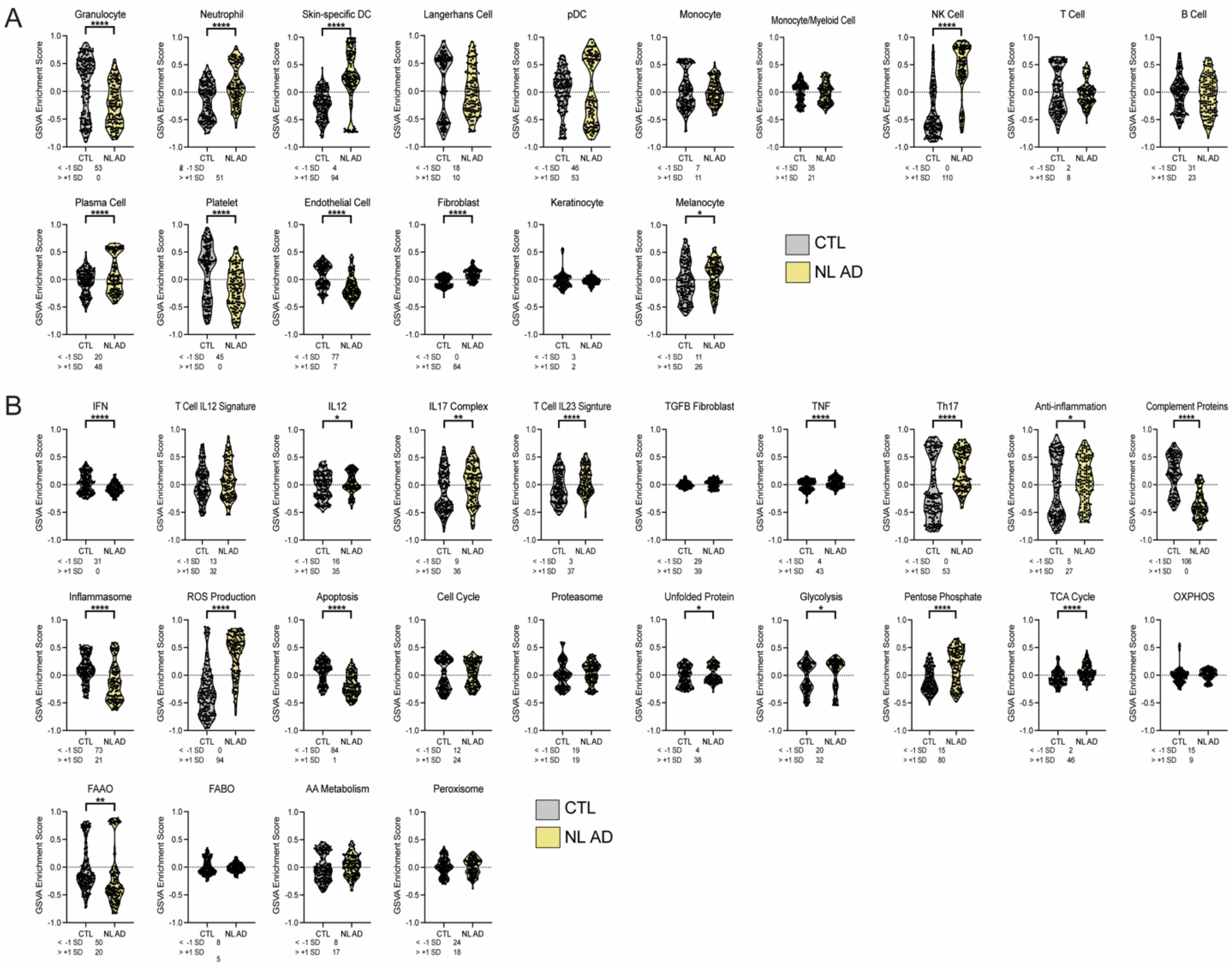
A
Granulocyte
Neutrophil
Skin-specific DC
Langerhans Cell
pDC
Monocyte
Monocyte/Myeloid Cell
NK Cell
T Cell
B Cell
Plasma Cell
Platelet
Endothelial Cell
Fibroblast
Keratinocyte
Melanocyte
GSVA Enrichment Score
CTL
NL AD
B
IFN
T Cell IL12 Signature
IL12
IL17 Complex
T Cell IL23 Signture
TGFB Fibroblast
TNF
Th17
Anti-inflammation
Complement Proteins
Inflammasome
ROS Production
Apoptosis
Cell Cycle
Proteasome
Unfolded Protein
Glycolysis
Pentose Phosphate
TCA Cycle
OXPHOS
FAAO
FABO
AA Metabolism
Peroxisome

Supplemental Figure 18: Analysis of cellular and molecular pathway signatures in nonlesional AD shows upregulation of anti-inflammation, neutrophil, NK cell and Th17 gene signatures. GSVA enrichment scores using Z-score transformation of (**A**) cellular gene signatures and (**B**) pathway gene signatures in nonlesional AD (light yellow) and control samples (grey). The number of nonlesional AD samples per dataset that lie -1 standard deviation of the mean of the control samples is denoted on the first subtext line. The number of AD samples per dataset that lie +1 standard deviation of the mean of the control samples is denoted on the second subtext line. Welch's t-test: * $p < 0.05$; ** $p < 0.01$; *** $p < 0.001$; **** $p < 0.0001$.

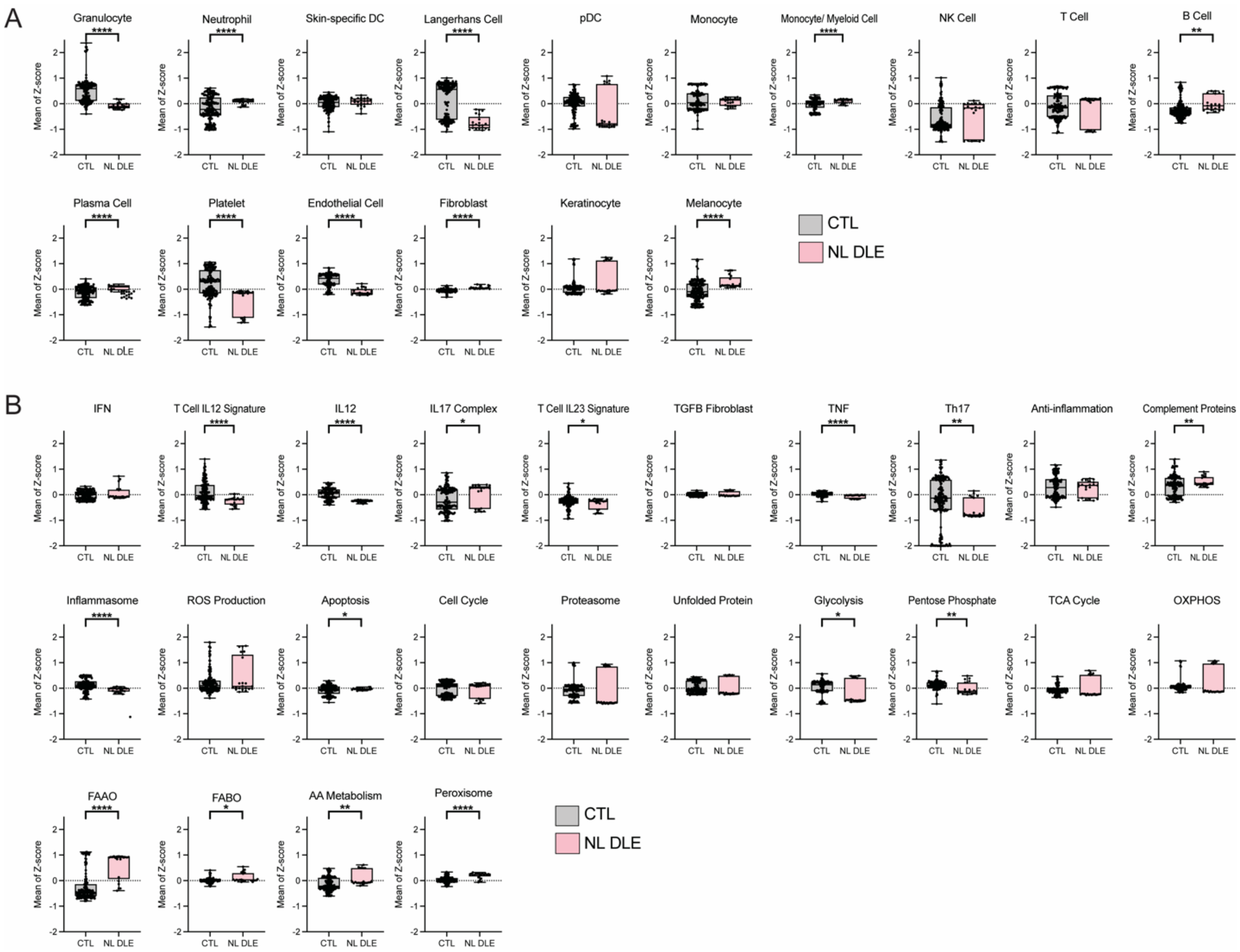
A
Granulocyte
Neutrophil
Skin-specific DC
Langerhans Cell
pDC
Monocyte
Monocyte/ Myeloid Cell
NK Cell
T Cell
B Cell
Plasma Cell
Platelet
Endothelial Cell
Fibroblast
Keratinocyte
Melanocyte
Mean of Z-score
CTL
NL DLE
B
IFN
T Cell IL12 Signature
IL12
IL17 Complex
T Cell IL23 Signature
TGFB Fibroblast
TNF
Th17
Anti-inflammation
Complement Proteins
Inflammasome
ROS Production
Apoptosis
Cell Cycle
Proteasome
Unfolded Protein
Glycolysis
Pentose Phosphate
TCA Cycle
OXPHOS
FAAO
FABO
AA Metabolism
Peroxisome
CTL
NL DLE

Supplemental Figure 19: Analysis of cellular and molecular pathway signatures in nonlesional DLE using mean of Z-score. Box plots of the mean of Z-scores of genes for each sample and gene category for (**A**) cellular gene signatures and (**B**) pathway gene signatures in nonlesional DLE (light pink) and control samples (grey). Welch's t-test: * $p < 0.05$; ** $p < 0.01$; *** $p < 0.001$; **** $p < 0.0001$.

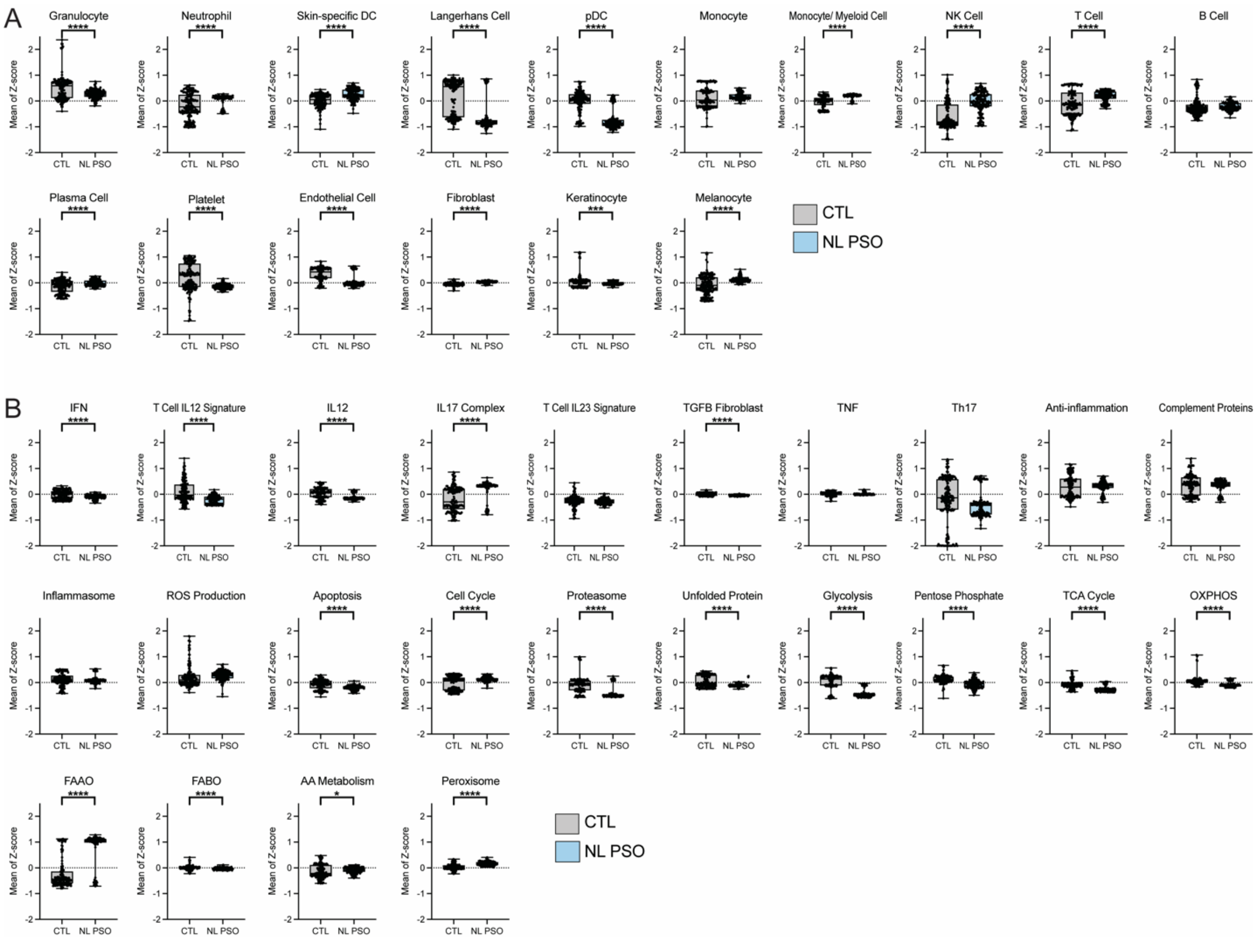
A
Granulocyte
Neutrophil
Skin-specific DC
Langerhans Cell
pDC
Monocyte
Monocyte/ Myeloid Cell
NK Cell
T Cell
B Cell
Plasma Cell
Platelet
Endothelial Cell
Fibroblast
Keratinocyte
Melanocyte
Mean of Z-score
CTL
NL PSO
B
IFN
T Cell IL12 Signature
IL12
IL17 Complex
T Cell IL23 Signature
TGFB Fibroblast
TNF
Th17
Anti-inflammation
Complement Proteins
Inflammasome
ROS Production
Apoptosis
Cell Cycle
Proteasome
Unfolded Protein
Glycolysis
Pentose Phosphate
TCA Cycle
OXPHOS
FAAO
FABO
AA Metabolism
Peroxisome

Supplemental Figure 20: Analysis of cellular and molecular pathway signatures in nonlesional PSO using mean of Z-score. Box plots of the mean of Z-scores of genes for each sample and gene category for (**A**) cellular gene signatures and (**B**) pathway gene signatures in nonlesional PSO (light blue) and control samples (grey). Welch's t-test: * $p < 0.05$; ** $p < 0.01$; *** $p < 0.001$; **** $p < 0.0001$.

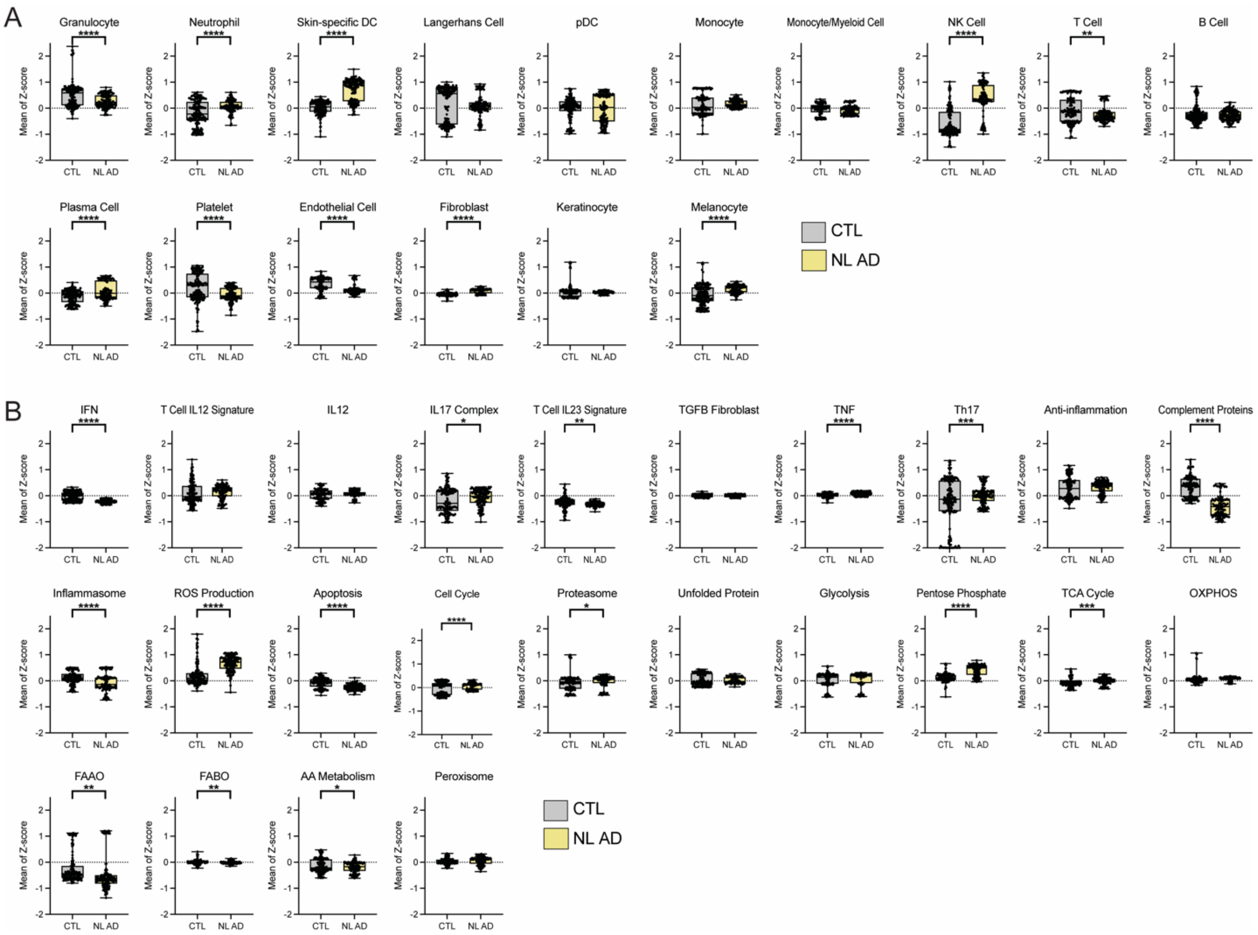

A
Granulocyte
Neutrophil
Skin-specific DC
Langerhans Cell
pDC
Monocyte
Monocyte/Myeloid Cell
NK Cell
T Cell
B Cell
Plasma Cell
Platelet
Endothelial Cell
Fibroblast
Keratinocyte
Melanocyte
CTL
NL AD
Mean of Z-score
B
IFN
T Cell IL12 Signature
IL12
IL17 Complex
T Cell IL23 Signature
TGFB Fibroblast
TNF
Th17
Anti-inflammation
Complement Proteins
Inflammasome
ROS Production
Apoptosis
Cell Cycle
Proteasome
Unfolded Protein
Glycolysis
Pentose Phosphate
TCA Cycle
OXPHOS
FAAO
FABO
AA Metabolism
Peroxisome
CTL
NL AD

Supplemental Figure 21: Analysis of cellular and molecular pathway signatures in nonlesional AD using mean of Z-score. Box plots of the mean of Z-scores of genes for each sample and gene category for (**A**) cellular gene signatures and (**B**) pathway gene signatures in nonlesional AD (light yellow) and control samples (grey). Welch's t-test: * $p < 0.05$; ** $p < 0.01$; *** $p < 0.001$; **** $p < 0.0001$.

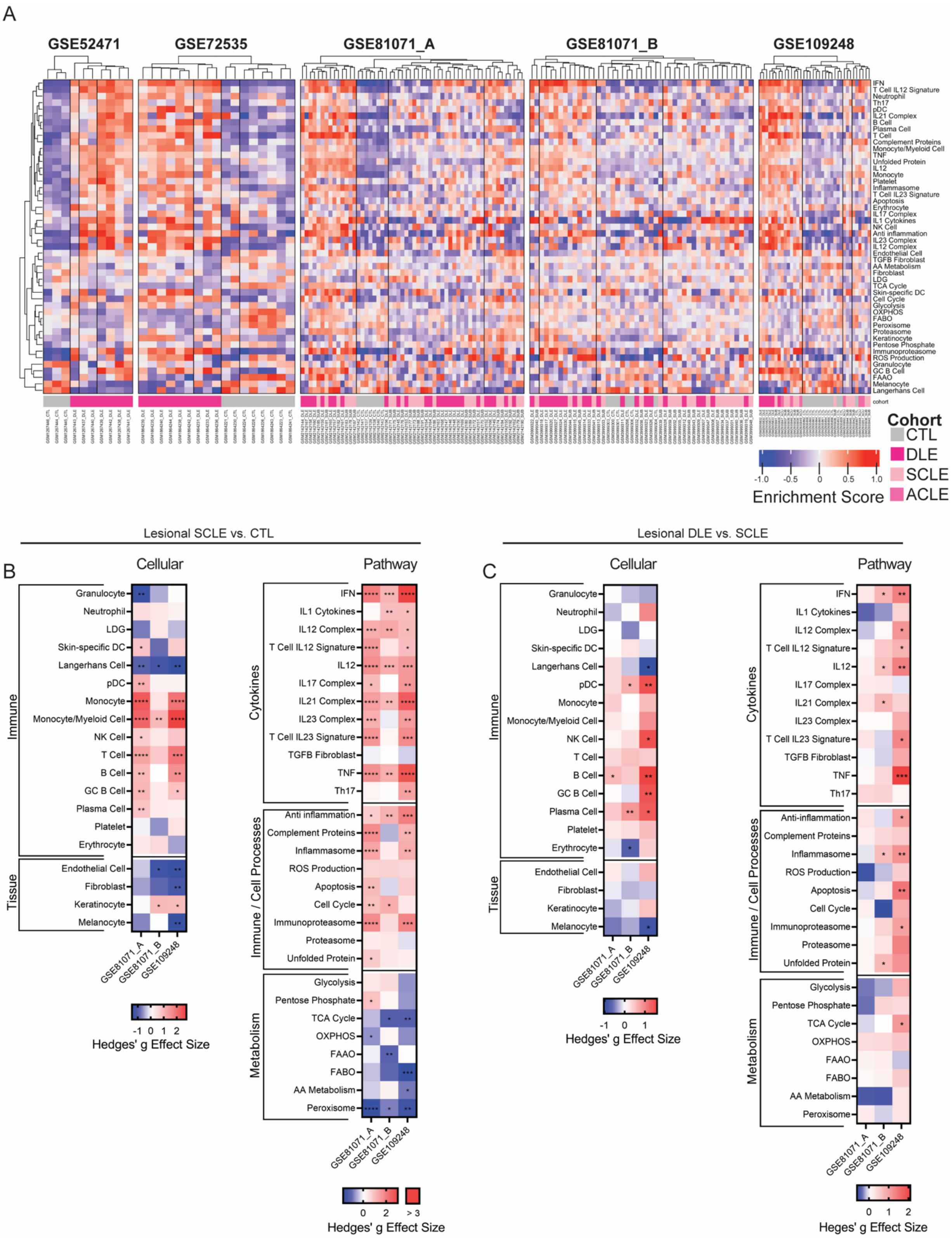

A
GSE52471
GSE72535
GSE81071_A
GSE81071_B
GSE109248
IFN
T Cell IL12 Signature
Neutrophil
Th17
pDC
IL21 Complex
B Cell
Plasma Cell
T Cell
Complement Proteins
Monocyte/Myeloid Cell
TNF
Unfolded Protein
IL12
Monocyte
Platelet
Inflammasome
T Cell IL23 Signature
Apoptosis
Erythrocyte
IL17 Complex
IL1 Cytokines
NK Cell
Anti inflammation
IL23 Complex
IL12 Complex
Endothelial Cell
TGFB Fibroblast
AA Metabolism
Fibroblast
LDG
TCA Cycle
Skin-specific DC
Cell Cycle
Glycolysis
OXPHOS
FABO
Peroxisome
Proteasome
Keratinocyte
Pentose Phosphate
Immunoproteasome
ROS Production
Granulocyte
GC B Cell
FAAO
Melanocyte
Langerhans Cell
cohort
Cohort
CTL
DLE
SCLE
ACLE
-1.0
-0.5
0
0.5
1.0
Enrichment Score
Lesional SCLE vs. CTL
Lesional DLE vs. SCLE
B
C
Cellular
Pathway
Immune
Tissue
Cytokines
Immune / Cell Processes
Metabolism
Endothelial Cell
Fibroblast
Keratinocyte
Melanocyte
Anti-inflammation
GSE81071_A
GSE81071_B
GSE109248
-1 0 1 2
Hedges' g Effect Size
0 2 > 3
Hedges' g Effect Size
-1 0 1
Hedges' g Effect Size
0 1 2
Heges' g Effect Size

Supplemental Figure 22: Cellular and pathway enrichment in SCLE is quantitatively similar to enrichment observed in DLE. (**A**) Hierarchical clustering (k=4 clusters) of DLE, SCLE, ACLE and healthy control samples from five lupus datasets using GSVA enrichment scores of cellular and pathway gene signatures. Hedges' g effect sizes of GSVA enrichment scores for (**B**) cellular gene signatures (left) and pathway gene signatures (right) in lesional SCLE and control samples in three datasets. Hedges' g effect sizes of GSVA enrichment scores for (**C**) cellular gene signatures (left) and pathway gene signatures (right) in lesional DLE and SCLE samples in three datasets. Heatmap visualization uses red (enriched signature, >0) and blue (decreased signature, <0). Welch's t-test: * $p < 0.05$; ** $p < 0.01$; *** $p < 0.001$; **** $p < 0.0001$.

A

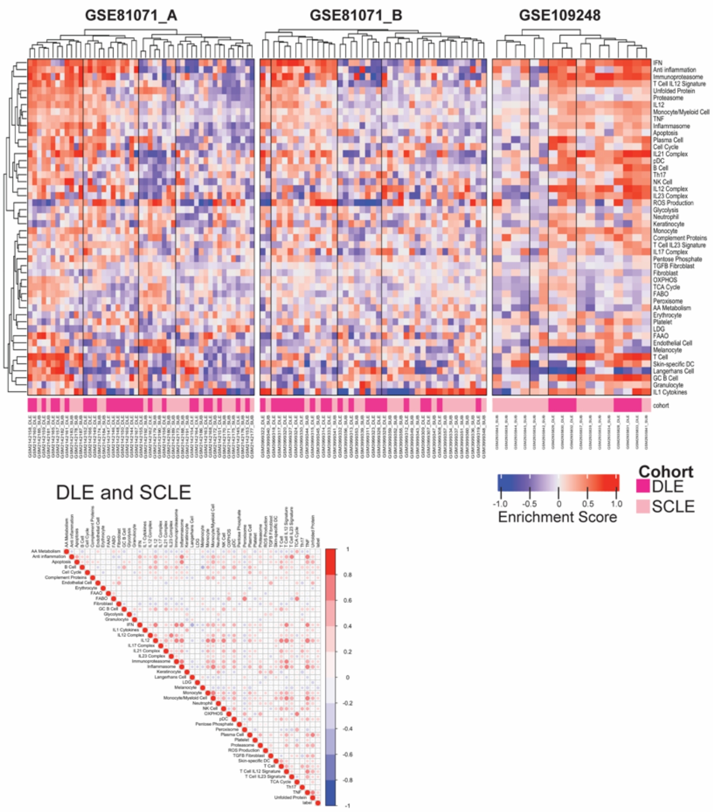

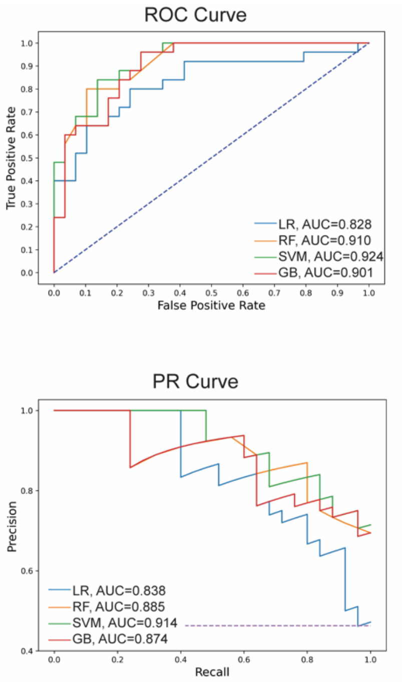

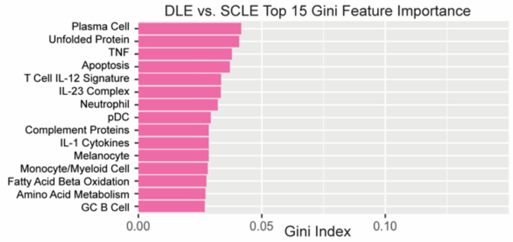

F

DLE vs. SCLE Results

Classifier	Sensitivity	Specificity	Cohen's kappa	Precision	f-1 score	Accuracy
Logistic R	0.72	0.79	0.51	0.75	0.73	0.76
Random F	0.72	0.90	0.62	0.86	0.78	0.81
SVM	0.64	0.93	0.58	0.89	0.74	0.80
GB	0.68	0.83	0.51	0.77	0.72	0.76

Supplemental Figure 23: DLE and SCLE can be transcriptionally classified using ML. (**A**) Hierarchical clustering (k=4) of DLE, SCLE and control samples from three lupus datasets based on GSVA scores of cellular and pathway gene signatures. (**B**) Correlation plot of GSVA enrichment scores of lesional DLE and lesional SCLE samples. (**C**) ROC curve and (**D**) PR curve separating DLE and SCLE using ML classifiers, including: logistic regression (LR, blue), random forest (RF, orange), support vector machine (SVM, green) and gradient boosting (GB, red). Random oversampling was used to adjust for class imbalance errors. (**E**) Top 15 features important in classifying DLE from SCLE using Gini feature importance. (**F**) Classification metrics including sensitivity, specificity, Cohen's kappa score, precision, f-1 score and accuracy to properly separate DLE and SCLE. Refer to **tables S3A-B** for details about ML.

A

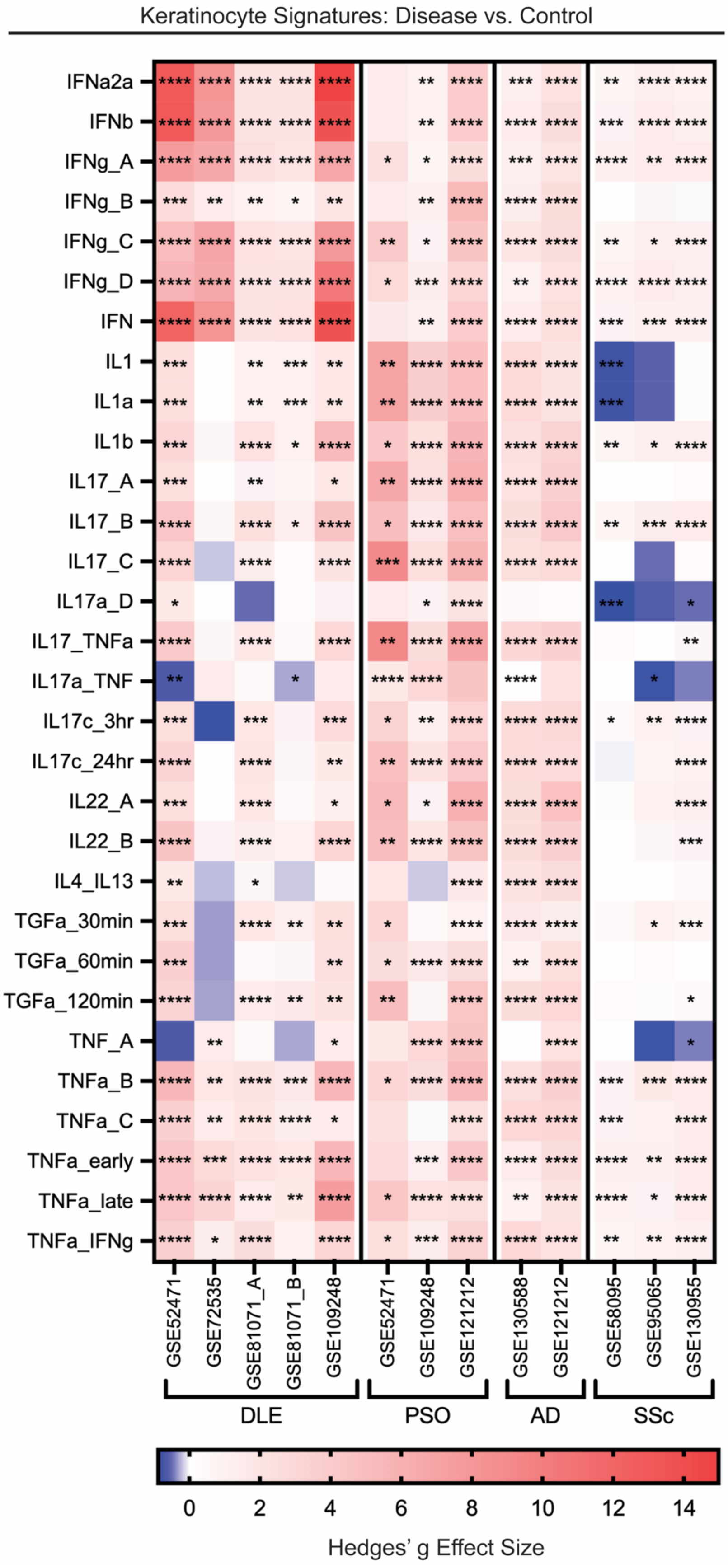

Keratinocyte Signatures: Disease vs. Control
IFNa2a
IFNb
IFNg_A
IFNg_B
IFNg_C
IFNg_D
IFN
IL1
IL1a
IL1b
IL17_A
IL17_B
IL17_C
IL17a_D
IL17_TNFa
IL17a_TNF
IL17c_3hr
IL17c_24hr
IL22_A
IL22_B
IL4_IL13
TGFa_30min
TGFa_60min
TGFa_120min
TNF_A
TNFa_B
TNFa_C
TNFa_early
TNFa_late
TNFa_IFNg
GSE52471
GSE72535
GSE81071_A
GSE81071_B
GSE109248
GSE52471
GSE109248
GSE121212
GSE130588
GSE121212
GSE58095
GSE95065
GSE130955
DLE
PSO
AD
SSc
0
2
4
6
8
10
12
14
Hedges' g Effect Size

Supplemental Figure 24: **Stimulated keratinocyte signatures are highly enriched in skin inflammatory diseases.** Hedges' g effect sizes of GSVA enrichment scores for lesional disease samples compared to their respective healthy control samples in five DLE, three PSO, two AD and three SSc datasets using curated keratinocyte-curated cellular signatures treated with various types of cytokines and immune molecules. Heatmap visualization uses red (enriched signature, >0) and blue (decreased signature, <0). Welch's t-test: * $p < 0.05$; ** $p < 0.01$; *** $p < 0.001$; **** $p < 0.0001$.

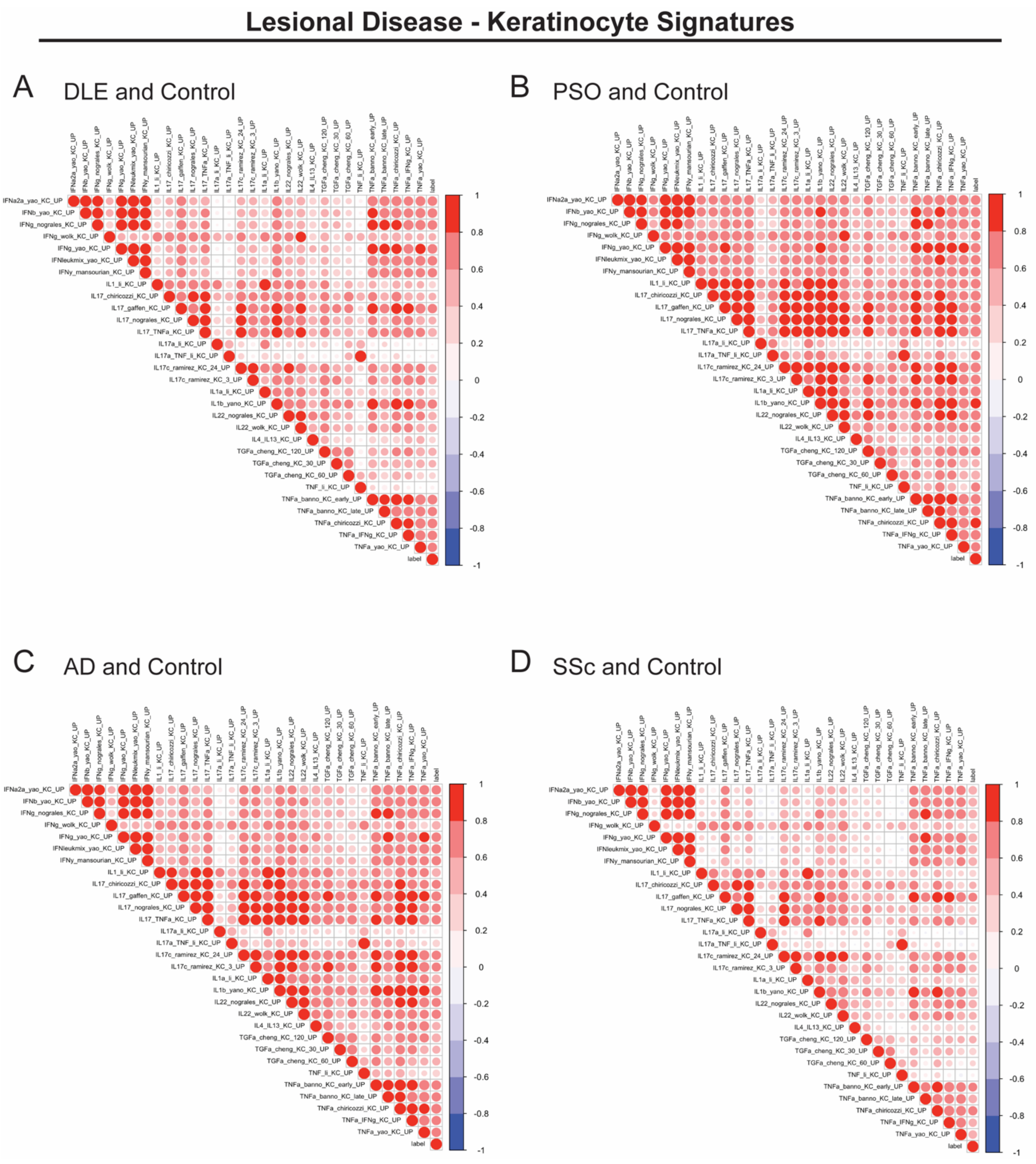
Lesional Disease - Keratinocyte Signatures
A
DLE and Control
B
PSO and Control
C
AD and Control
D
SSc and Control

Supplemental Figure 25: **Overabundance of correlated features from keratinocyte cell gene signatures.** Correlation plot of GSVA enrichment scores to find keratinocyte gene signatures that are correlated to each other in (**A**) lesional DLE and control samples; (**B**) lesional PSO and control samples; (**C**) lesional AD and control samples; and (**D**) lesional SSc and control samples.

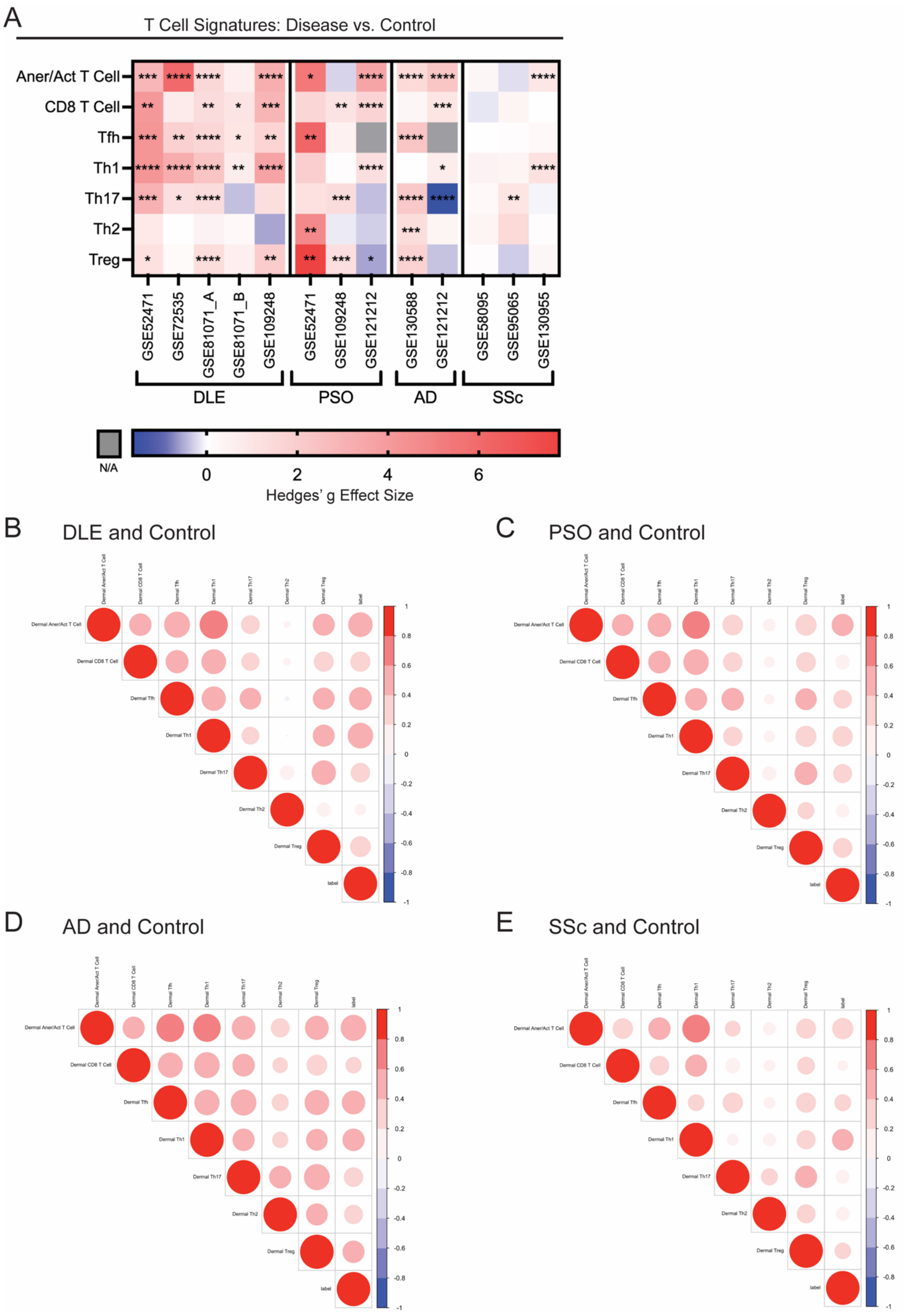
A
T Cell Signatures: Disease vs. Control
Aner/Act T Cell
CD8 T Cell
Tfh
Th1
Th17
Th2
Treg
GSE52471
GSE72535
GSE81071_A
GSE81071_B
GSE109248
GSE52471
GSE109248
GSE121212
GSE130588
GSE121212
GSE58095
GSE95065
GSE130955
DLE
PSO
AD
SSc
N/A
0
2
4
6
Hedges' g Effect Size
B
DLE and Control
C
PSO and Control
D
AD and Control
E
SSc and Control
Dermal Aner/Act T Cell
Dermal CD8 T Cell
Dermal Tfh
Dermal Th1
Dermal Th17
Dermal Th2
Dermal Treg
label
1
0.8
0.6
0.4
0.2
0
-0.2
-0.4
-0.6
-0.8
-1

Supplemental Figure 26: **T cell subtype signatures are highly enriched in skin inflammatory diseases.** GSVA enrichment scores for (**A**) T cell cellular signatures in lesional disease samples compared to their respective healthy control samples in five DLE, three PSO, two AD and three SSc datasets. Heatmap visualization uses red (enriched signature, >0) and blue (decreased signature, <0). Welch's t-test: * $p < 0.05$; ** $p < 0.01$; *** $p < 0.001$; **** $p < 0.0001$. Correlation plots of GSVA enrichment scores to find T cell gene signatures that are correlated to each other in (**B**) DLE and control samples; (**C**) PSO and control samples; (**D**) AD and control samples; and (**E**) SSc and control samples.

Supplemetal Table 1: Publicly available datasets that were used in this study.

GEO number	Reference	Platform	Samples used in this study	Paired/unpaired	Description	Probes	After IQR
**GSE52471**	(*1*)	[HG-U133A_2] Affymetrix Human Genome U133A 2.0 Array	3 PSO, 7 DLE, 3 CTL	Unpaired	3 PSO, 7 DLE and 3 CTL samples that belong to the same batch were included in this analysis.	22215	10045
**GSE72535**	(*2*)	Illumina HumanHT-12 V4.0 expression beadchip	9 DLE, 8 CTL	Unpaired	This is the only Lupus dataset which has clinical information such as SLEDAI, CLASI.A, CLASI.D, dsDNA, C3, C4 available.	47322	20762
**GSE810171**	(*3-6*)	[HuGene-2_1-st] Affymetrix Human Gene 2.1 ST Array [HuGene21st_Hs_ENTREZG_19.0.0]	26+21 DLE, 23+20 SCLE, 7+6 CTL	Unpaired	The original data set from GEO, as confirmed by the authors of the paper contains 6 batches H1-H6. We observed that batches H1-H4 were uploaded on GEO in the year 2017 and batches H5-H6 were uploaded on GEO in the year 2019. For the purpose of our analysis we have divided this dataset into two parts 2017 (H1-H4) and 2019(H5-H6). Before splitting into two, we normlised original eset with 11 HK genes. Method used for normalisation is as follows: Take mean expression of HK genes per sample and divide every gene expression value of that sample with mean value.	53617	26536
**GSE109248**	(*7*)	Illumina HumanHT-12 V4.0 expression beadchip	6DLE, 11SCLE, 17PSO ,13CTL	Unpaired	From the original data on GEO 1 ACU, 1 SUB,1 CTL was removed as outliers in the PCA plot. The only clinical information available is fas ligand levels.	47302	31415
**GSE100093**	(*8*)	[HT_HG-U133_Plus_PM] Affymetrix HT HG-U133+ PM Array Plate	15L,15NL (DLE)	Paired	Only lupus dataset with time points skin biopsies available. The clinical trial was carried out in two sequences. Sequence 1 (9 patients) were given Amgen 811, Sequence 2 (7 pateints) were given placebo. The skin biopsies were taken at day 0, day 15 and day 57. For the purpose of our analysis, Lesional and Non-lesional biopsies from both the sequences patients at day 0 without any drug treatment used. 1 outlier patient's L and NL biopsies were removed.	54613	20200
**GSE120809**	(*9*)	[HG-U133A_2] Affymetrix Human Genome U133A Array (HGU133A2 Hs ENTREZG 21.0.0)	6L, 6NL (DLE)	Paired	There were total 7 patients. 3 patients had NL biopsies as technical replicates. Technical replicates were removed. Plus one outlier patient's L and NL biopsies were removed.	22215	10072
**GSE117239**	(*10*)	[HG-U133_Plus_2] Affymetrix Human Genome U133 Plus 2.0 Array	63L, 63NL (PSO)	Paired	These are all psoriasis patients that were treated with three drugs, 15 patients with ustekinumab 45 mg, 23 patients with ustekinumab 90 mg and 25 patients with ethanercept. Skin biopsies were taken at Baseline, Week 1 and Week 12. Clinical information availble is pasi score, respose and batch. This dataset does have some batch effects but no analysis issues. For our analysis patients at baseline were used.	54613	14627
**GSE117468**	(*11*)	[HG-U133_Plus_2] Affymetrix Human Genome U133 Plus 2.0 Array	73L, 73NL (PSO)	Paired	These are all psoriasis patients that were treated with 4 drugs, 31 patients with broadalumab 140 mg, 21 patients with broadalumab 210 mg, 9 patients with placebo and 12 patients with Ustekinumab. Skin biopsies were taken at Baseline, Week 4 and Week 12. Clinical information availble is pasi score, age, race, weight and bmi. This dataset may have some batch effects and overlap woth GSE117239 but we have not done nothig specific to deal with this problem. For our analysis patients at baseline were used.	54613	20200
**GSE130588**	(*12*)	[HG-U133_Plus_2] Affymetrix Human Genome U133 Plus 2.0 Array	19L,19NL (AD), 20CTL	Paired and Unpaired both	These are all atopic dermatitis patients and healthy individuals. Atopic dermatitis patients were treated with 2 drugs. 13 patients with Dupilumab and 6 patients with placebo. Lesional skin biopsies were taken at Baseline, Week 4 and Week 16.NL were taken only at baseline. Clinical information availble is easi, race, sex, and scorad. For our analysis patients at baseline were used.	54613	15738
**GSE137430**	(*13*)	Illumina HiSeq 4000 (Homo sapiens)	38L, 38NL (AD)	Paired	These are all atopic dermatitis patients. Atopic dermatitis patients were treated with 2 drugs. 14 patients with Secukinumab and 8 patients with placebo. Lesional skin biopsies were taken at Baseline, Week 4 and Week 16. No clinical information is availble.For our analysis patients at baseline were used.	18195	16594
**GSE157194**	(*14*)	Illumina HiSeq 3000 (Homo sapiens)	54L, 54NL (AD)	Paired	These are all atopic dermatitis patients. Atopic dermatitis patients were treated with 2 drugs. 7 patients with cyclosporin and 17 patients with dupilumab. Lesional skin biopsies were taken at month 0 and month 3. No clinical information is availble. For our analysis patients at baseline were used.	16873	15713
**GSE121212**	(*15*)	Illumina HiSeq 2500 (Homo sapiens)	27L & 27NL (PSO), 21L & 21NL (AD), 38CTL	Paired and Unpaired both	The original data on GEO also had chronic AD patients. I removed them from analysis because we not interested in subtype of atopic dermatitis disease. It has lesional and non-lesional biopsies of ATD patients and PSO patients. As well as biopsiy from control individuals.	15660	14696
**GSE95065**	**not published**	[HG-U133A_2] Affymetrix Human Genome U133A 2.0 Array (HGU133A2 Hs ENTREZG 19.0.0)	18 SSc,4CTL	Unpaired	The original data on GEO had 4 batches, only used patients from batch 2.	22215	9765
**GSE58095**	(*16*)	Illumina HumanHT-12 V4.0 expression beadchip	39 SSc, 36 CTL	Unpaired	The original data on GEO had limited and diffuse scleroderma patients. Only diffuse scleroderma patients and healthy patients skin biopsies were used.	47279	31399
**GSE130955**	(*17*)	Illumina HiSeq 2500 (Homo sapiens)	58 SSc, 33 CTL	Unpaired	This dataset may have some batch effect. And patients could have been treated earlier with Mycophenolate, Methotrexate / Cyclophosphamid.	18972	16800

References:

1. A. Jabbari, M. Suárez-Fariñas, J. Fuentes-Duculan, J. Gonzalez, I. Cueto, A. G. Franks, J. G. Krueger, Dominant Th1 and minimal Th17 skewing in discoid lupus revealed by transcriptomic comparison with psoriasis, *J. Invest. Dermatol.* **134**, 87–95 (2014).

2. B. F. Chong, L. chiang Tseng, G. A. Hosler, N. M. Teske, S. Zhang, D. R. Karp, N. J. Olsen, C. Mohan, A subset of CD163+ macrophages displays mixed polarizations in discoid lupus skin, *Arthritis Res. Ther.* **17**, 324 (2015).

3. J. Liu, C. C. Berthier, J. M. Kahlenberg, Enhanced Inflammasome Activity in Systemic Lupus Erythematosus Is Mediated via Type I Interferon–Induced Up-Regulation of Interferon Regulatory Factor 1, *Arthritis Rheumatol.* **69**, 1840–1849 (2017).

4. M. K. Sarkar, G. A. Hile, L. C. Tsoi, X. Xing, J. Liu, Y. Liang, C. C. Berthier, W. R. Swindell, M. T. Patrick, S. Shao, P. S. Tsou, R. Uppala, M. A. Beamer, A. Srivastava, S. L. Bielas, P. W. Harms, S. Getsios, J. T. Elder, J. J. Voorhees, J. E. Gudjonsson, J. M. Kahlenberg, Photosensitivity and type i IFN responses in cutaneous lupus are driven by epidermal-derived interferon kappa, *Ann. Rheum. Dis.* **77**, 1653–1664 (2018).

5. L. C. Tsoi, G. A. Hile, C. C. Berthier, M. K. Sarkar, T. J. Reed, J. Liu, R. Uppala, M. Patrick, K. Raja, X. Xing, E. Xing, K. He, J. E. Gudjonsson, J. M. Kahlenberg, Hypersensitive IFN Responses in Lupus Keratinocytes Reveal Key Mechanistic Determinants in Cutaneous Lupus, *J. Immunol.* **202**, 2121–2130 (2019).

6. A. C. Billi, M. Gharaee-Kermani, J. Fullmer, L. C. Tsoi, B. D. Hill, D. Gruszka, J. Ludwig, X. Xing, S. Estadt, S. J. Wolf, S. M. Rizvi, C. C. Berthier, J. B. Hodgin, M. A. Beamer, M. K. Sarkar, Y. Liang, R. Uppala, S. Shao, C. Zeng, P. W. Harms, M. E. Verhaegen, J. J. Voorhees, F. Wen, N. L. Ward, A. A. Dlugosz, J. M. Kahlenberg, J. E. Gudjonsson, The female-biased factor VGLL3 drives cutaneous and systemic autoimmunity, *JCI Insight* **4** (2019), doi:10.1172/jci.insight.127291.

7. P. Mande, B. Zirak, W. C. Ko, K. Taravati, K. L. Bride, T. Y. Brodeur, A. Deng, K. Dresser, Z. Jiang, R. Ettinger, K. A. Fitzgerald, M. D. Rosenblum, J. E. Harris, A. Marshak-Rothstein, Fas ligand promotes an inducible TLR-dependent model of cutaneous lupus–like inflammation, *J. Clin. Invest.* **128**, 2966–2978 (2018).

8. V. P. Werth, D. Fiorentino, B. A. Sullivan, M. J. Boedigheimer, K. Chiu, C. Wang, G. E. Arnold, M. A. Damore, J. Bigler, A. A. Welcher, C. B. Russell, D. A. Martin, J. B. Chung, Brief Report: Pharmacodynamics, Safety, and Clinical Efficacy of AMG 811, a Human Anti–Interferon-γ Antibody, in Patients With Discoid Lupus Erythematosus, *Arthritis Rheumatol.* **69**, 1028–1034 (2017).

9. A. M. S. Barron, J. C. Mantero, J. D. Ho, B. Nazari, K. L. Horback, J. Bhawan, R. Lafyatis, C. Lam, J. L. Browning, Perivascular Adventitial Fibroblast Specialization Accompanies T Cell Retention in the Inflamed Human Dermis, *J. Immunol.* **202**, 56–68 (2019).

10. C. Brodmerkel, K. Li, S. Garcet, K. Hayden, A. Chiricozzi, I. Novitskaya, J. Fuentes-Duculan, M. Suarez-Farinas, K. Campbell, J. G. Krueger, Modulation of inflammatory gene transcripts in psoriasis vulgaris: Differences between ustekinumab and etanercept, *J. Allergy Clin. Immunol.* **143**, 1962–1965 (2019).

11. L. E. Tomalin, C. B. Russell, S. Garcet, D. A. Ewald, P. Klekotka, A. Nirula, H. Norsgaard, M. Suàrez-Fariñas, J. G. Krueger, Short-term transcriptional response to IL-17 receptor-A antagonism in the treatment of psoriasis, *J. Allergy Clin. Immunol.* **145**, 922–932 (2020).

12. E. Guttman-Yassky, R. Bissonnette, B. Ungar, M. Suárez-Fariñas, M. Ardeleanu, H. Esaki, M. Suprun, Y. Estrada, H. Xu, X. Peng, J. I. Silverberg, A. Menter, J. G. Krueger, R. Zhang, U. Chaudhry, B. Swanson, N. M. H. Graham, G. Pirozzi, G. D. Yancopoulos, J. D. Jennifer, Dupilumab progressively improves systemic and cutaneous abnormalities in patients with atopic dermatitis, *J. Allergy Clin. Immunol.* **143**, 155–172 (2019).

13. B. Ungar, A. B. Pavel, R. Li, G. Kimmel, J. Nia, P. Hashim, H. J. Kim, M. Chima, A. S. Vekaria, Y. Estrada, H. Xu, X. Peng, G. K. Singer, D. Baum, Y. Mansouri, M. Taliercio, E. Guttman-Yassky, Phase 2 randomized, double-blind study of IL-17 targeting with secukinumab in atopic dermatitis, *J. Allergy Clin. Immunol.* **147**, 394–397 (2021).

14. L. Möbus, E. Rodriguez, I. Harder, D. Stölzl, N. Boraczynski, S. Gerdes, A. Kleinheinz, S. Abraham, A. Heratizadeh, C. Handrick, E. Haufe, T. Werfel, J. Schmitt, S. Weidinger, Atopic dermatitis displays stable and dynamic skin transcriptome signatures, *J. Allergy Clin. Immunol.* **147**, 213–223 (2021).

15. L. C. Tsoi, E. Rodriguez, F. Degenhardt, H. Baurecht, U. Wehkamp, N. Volks, S. Szymczak, W. R. Swindell, M. K. Sarkar, K. Raja, S. Shao, M. Patrick, Y. Gao, R. Uppala, B. E. Perez White, S. Getsios, P. W. Harms, E. Maverakis, J. T. Elder, A. Franke, J. E. Gudjonsson, S. Weidinger, Atopic Dermatitis Is an IL-13–Dominant Disease with Greater Molecular Heterogeneity Compared to Psoriasis, *J. Invest. Dermatol.* **139**, 1480–1489 (2019).

16. S. Assassi, W. R. Swindell, M. Wu, F. D. Tan, D. Khanna, D. E. Furst, D. P. Tashkin, R. R. Jahan-Tigh, M. D. Mayes, J. E. Gudjonsson, J. T. Chang, Dissecting the heterogeneity of skin gene expression patterns in systemic sclerosis, *Arthritis Rheumatol.* **67**, 3016–3026 (2015).

17. B. Skaug, D. Khanna, W. R. Swindell, M. E. Hinchcliff, T. M. Frech, V. D. Steen, F. N. Hant, J. K. Gordon, A. A. Shah, L. Zhu, J. Zheng, J. L. Browning, A. M. S. S. Barron, M. Wu, S. Visvanathan, P. Baum, J. M. Franks, M. L. Whitfield, V. K. Shanmugam, R. T. Domsic, F. V. Castelino, E. J. Bernstein, N. Wareing, M. A. Lyons, J. Ying, J. Charles, M. D. Mayes, S. Assassi, Global skin gene expression analysis of early diffuse cutaneous systemic sclerosis shows a prominent innate and adaptive inflammatory profile, *Ann Rheum Dis* **79**, 379–386 (2020).

Table S1: Publicly available datasets that were used in this study.

Supplemental Table 2A: Genes within cell signatures.

Cell Signature	Genes in the signature
**B Cell**	AICDA, BANK1, BLK, BLNK, CD19, CD22, CD79A, CD79B, CLEC17A, CR2, DAPP1, DTX1, FCRL1, FCRL2, FCRL3, FCRL4, FCRL5, HLA-DOB, IGHD, IGHM, LY6D, MS4A1, PAX5, POU2AF1, SH2B2, TNFRSF13B, TNFRSF13C, VPREB1, VPREB3, ZBTB32, ZNF318
**Endothelial Cell**	DLC1, ECSCR, EMCN, FLT1, KDR, LDB2, LRRC32, MEIS2, PLAT, PTPRB, SELE, TM4SF1, TM4SF18, VWF
**Erythrocyte**	BSG, GFI1B, GYPA, GYPB, GYPE, ICAM4, KEL, NFE2, RHD, SLC4A1, TRIM10, TSPO2
**Fibroblast**	43894, ADAM33, ADAMTS6, AGTR1, ALPK2, ANGPTL2, ANKRD45, ANO2, ANPEP, ARMC9, ASPN, BDKRB2, BDNF, BMPER, C11orf87, C1R, C1S, CATSPER3, CCDC102B, CCDC80, CCDC81, CEMIP, CHAC1, CHRM2, CLMP, CNN1, COL14A1, COL3A1, COL5A1, COLEC10, CPXM2, CPZ, CRABP2, CXCL12, DCN, DDR2, DKK1, DMRTA1, EGFL6, ELOVL2, EMILIN1, FAM180A, FBLN7, FBN1, FGF5, FMN2, FOXF2, FST, FSTL1, GFRA1, GLIS1, GLT8D2, GPR176, GREM1, GREM2, GRIK2, GUCY1A2, HSD17B2, HSPA2, HSPB3, IL19, IQCD, KCNMB2, KIRREL3, KRT34, KRTAP1-5, KRTAP3-1, L1CAM, LAYN, LMOD1, LOXL4, LUM, LY6K, MFAP4, MFAP5, MGARP, MGP, MKX, MMP2, MXRA5, MXRA8, MYPN, NEXN, NFASC, NID2, NTF3, OLFML3, P3H3, P4HA3, PAMR1, PAX3, PCDHGA2, PCDHGA3, PCDHGA7, PDE8B, PDGFRA, PDGFRL, PDZRN3, PLA2R1, PLEKHA4, PLPP4, PLPPR4, PRKG1, PSG5, PTPRQ, PTRF, SEMA3A, SEMA5A, SEMA6D, SHOX, SLC16A2, SMIM2, SPARCL1, SPHKAP, SSTR1, STC1, STXBP6, SUSD5, SVEP1, TBX15, THBS2, TIMP2, TIMP3, TMEM119, TMEM130, TMEM47, TRHDE, TRPC4, UACA, UBL4B, VAT1L, VEGFC, WNT5A, WNT5B, ZFPM2
**GC B Cell**	FCRLA, GCSAM, KLHL6, LRMP, NUGGC, RGS13
**Granulocyte**	CLC, HSH2D, MS4A2, PGLYRP1, PRG2, SYNE1
**Keratinocyte**	ABLIM, AKR1C1, ALDH8, ALOX12B, ANXA8, AQP3, ATDC, BPAG1, CA12, CCND2, CD24, CDH3, CDKN1A, CDSN, COL17A1, CST6, CSTA, DD96, DSC1, DSG1, DSP, EGFR, EVPL, FLG, G0S2, GJA1, GLUL, GNA15, HBP17, IFI27, IFITM1, IGFBP7, ITGA3, ITGB4, IVL, JUNB, JUP, KLK11, KLK7, KLK8, KRT1, KRT14, KRT15, KRT16, KRT2A, KRT5, KRT6A, LAMA3, LGALS7, LOR, NOTCH3, PPL, PRDX2, PRSS11, PRSS2, PRSS4, S100A2, SERPINB2, SERPINB3, SERPINB4, SERPINB5, SERPINB7, SERPINE1, SFN, SPINK5, SPRR1B, TACSTD2, TFAP2A, TGM1, TP63, TUBA1, XP5
**Langerhans Cell**	CD1B, CD1C, CD1E, CD207
**LDG**	AZU1, CAMP, CEACAM3, CEACAM4, CEACAM6, CEACAM8, CTSG, DEFA4, ELANE, MPO, OLFM4, RNASE3
**Melanocyte**	ASIP, CITED1, DCT, GPNMB, GPR143, MITF, MLANA, MLPH, OCA2, PMEL, SLC24A5, SLC45A2, TYR, TYRP1
**Monocyte**	ADGRE1, C1QA, C1QB, C1QC, C2, CD14, CD300C, CD300E, CD5L, CD68, CLEC5A, CSF1R, CYBB, FOLR2, LILRA1, MARCO, MERTK, MS4A7, MSR1, SPIC
**Monocyte/Myeloid Cell**	ADGRE2, ADGRE3, AIF1, APOC1, BPI, BST1, C4A, C4B, C4BPA, C4BPB, C5, C6, C8A, C9, CD163, CD1D, CD209, CD300LF, CD33, CFD, CFP, CHIT1, CLEC12A, CLEC12B, CLEC1A, CLEC4A, CLEC4D, CLEC4E, CLEC4G, CLEC6A, CLEC7A, CRISP3, CSF2RA, CSF2RB, CST3, CTSS, F12, FCER1A, FCER1G, FCGR1A, FCGR1B, FCGR2A, FCGR2C, FLT3, GRN, IGSF6, ITGAX, LGALS12, LGALS4, LGALS9, LILRA2, LILRA5, LILRA6, LILRB2, LY86, LYVE1, LYZ, MEFV, MMP8, MNDA, MPEG1, MS4A4A, MS4A6A, NLRP12, NLRP3, NOD2, OLR1, OSCAR, OSM, PILRA, PRAM1, RETN, S100A12, S100A8, S100A9, SCARB1, SECTM1, SEMA4A, SERPING1, SGK1, SIGLEC1, SIGLEC10, SIGLEC14, SIGLEC5, SLC11A1, SLITRK4, SMPDL3B, SPI1, TEK, THBD, TLR2, TLR8, TNFSF13B, TREM1, TREML4, TYROBP, VENTX, VSIG4, VSTM1
**Neutrophil**	ARG1, BMX, CD177, CSF3R, DEFA1, DEFA1B, DEFA3, DEFB103A, DEFB103B, DEFB106B, DEFB136, DEFB4A, FPR2, OR1J2, PRTN3, SLC2A3, SLPI
**NK Cell**	KIR2DL4, KLRC3, KLRF1, NCAM1, NCR1, SH2D1B, TNFSF11, TXK
**pDC**	CLEC4C, IRF7, LILRA4, NRP1, PACSIN1, PLA2G5, PLAC8, PTCRA, SERPINF1, SLC15A4, TCF4
**Plasma Cell**	CD38, CRELD2, ELL2, FKBP11, IGKV4-1, IGLV2-14, ITM2C, JCHAIN, MANF, MZB1, PDIA4, PRDX4, SDF2L1, SPATS2, TNFRSF17, UAP1
**Platelet**	GP1BA, GP5, GP6, GP9, PF4, PF4V1, PLEK, PPBP, SLC35D3
**Skin-specific DC**	CLEC10A, CLEC9A, GPR31, MRC1, XCR1
**T Cell**	BCL11B, CAMK4, CD28, CD3D, CD3G, CD5, CD6, GPR171, ITK, RGCC, TESPA1, THEMIS, TRAT1

Table S2A: Genes within cell signatures.

Supplemental Table 2B: Genes within pathway signatures.

Pathway Signature	Genes in pathway signature
**AA Metabolism**	AASS, AGXT, AGXT2, ALDH4A1, ALDH7A1, ARG1, GAD1, GLUD1, GOT1, GPT2, GRHPR, IVD, MCCC2, OAT, OTC, OXCT1, PC, PSAT1, SDS, IL1RN, SOCS3, TNFAIP3
**Anti-inflammation**	IL1RN, SOCS3, TNFAIP3
**Apoptosis**	AIFM1, BAD, BAK1, BAX, BID, CASP10, CASP3, CASP6, CASP7, CASP8, CASP9, ENDOG, FAS, HTRA2, TNFRSF1A
**Cell Cycle**	ASPM, AURKA, AURKB, BRCA1, CCNB1, CCNB2, CCNE1, CDC20, CENPM, CEP55, E2F3, GINS2, MCM10, MCM2, MKI67, NCAPG, NDC80, PTTG1, TYMS
**Complement Proteins**	C1QA, C1QB, C1QC, C1R, C1S, C2, C3, C4A, C4B, C5, C6, C7, C8A, C8B, C8G, C9
**FAAO**	HAAO, HACL1, PEX13, PHYH, SLC27A2
**FABO**	ABCD1, ABCD2, ABCD3, ACAA2, ACACB, ACAD11, ACADL, ACADM, ACADS, ACADVL, ACAT1, ACAT2, ACOX1, ACOX2, ACOX3, ACOXL, ACSBG2, ACSL5, ADIPOQ, AKT2, AUH, BDH2, CPT1A, CPT2, CROT, DECR1, ECHDC1, ECHDC2, ECHS1, ECI1, ECI2, EHHADH, ETFA, ETFB, ETFDH, FABP1, GCDH, HADH, HADHA, HADHB, HIBCH, HSD17B4, IRS1, IRS2, IVD, LEP, PEX2, PEX5, PEX7, SESN2, SLC25A17, SLC27A2, TWIST1
**Glycolysis**	ALDOA, ALDOB, ALDOC, ENO1, ENO2, ENO3, GAPDH, GCK, GPI, HK1, HK2, HK3, HKDC1, LDHA, LDHAL6A, LDHAL6B, LDHB, LDHC, PFKFB1, PFKFB2, PFKFB3, PFKFB4, PFKL, PFKM, PFKP, PGAM1, PGAM2, PGK1, PGK2, PKM, SLC2A1, SLC2A3, SLC2A4, TPI1
**IFN**	EIF2AK2, GBP1, GBP2, GBP4, HERC5, HERC6, IFI27, IFI30, IFI35, IFI44, IFI44L, IFI6, IFIT1, IFIT2, IFIT3, IFIT5, IFITM1, IFITM2, IFITM3, ISG15, ISG20, MX1, MX2, OAS1, OAS2, OAS3, OASL, RSAD2, SAMD9, SAMD9L, SP100, SP110
**IL1 Cytokines**	IL18, IL1B
**IL12 Complex**	IL12A, IL12B, IL12RB1, IL12RB2
**IL12**	ACLY, AKAP10, APOL3, BACH2, BRCA2, CALD1, CASK, CASP1, CCR5, CDKN3, CXCL10, CXCL9, CYBB, DEFA1, ETAA1, FASLG, FBXL2, FCER2, FCGR1A, GBP1, GBP2, GLS, GNPDA1, GSTM5, GZMB, HHEX, HP, HSPA6, IFNG, IL16, IL18BP, IL18R1, IL1A, INPP5D, INSIG1, IRF1, KLF2, KRT8, LIMK1, LINC00597, LY75, MMP25, NIN, NLRP1, PCDH9, SELL, SERPIND1, SLAMF1, SOCS1, STAT1, TAP2, TBX21, TFF1, TNFAIP2, TNFAIP3, TNFSF10, TXK
**IL17 Complex**	IL17A, IL17F, IL17RA, IL17RC, TRAF3, TRAF3IP2
**IL21 Complex**	IL21, IL21R, IL2RG
**IL23 Complex**	IL12B, IL12RB1, IL23A, IL23R
**Immunoproteasome**	PSMB10, PSMB8, PSMB9
**Inflammasome**	AIM2, CASP1, CASP5, CTSB, GSDMB, GSDMD, NAIP, NEK7, NLRC4, NLRP1, NLRP3, NOD2, P2RX7, PANX1, PYCARD, RIPK1
**OXPHOS**	ATP5A1, ATP5B, ATP5D, ATP5E, ATP5F1, ATP5G1, ATP5G2, ATP5G3, ATP5H, ATP5I, ATP5J, ATP5J2, ATP5L, ATP5O, ATP5S, BCS1L, CEP89, COA1, COA3, COA4, COA5, COA6, COA7, COX10, COX10-AS1, COX11, COX14, COX15, COX16, COX17, COX18, COX19, COX20, COX4I1, COX4I2, COX5A, COX5B, COX6A1, COX6A2, COX6B1, COX6B2, COX6C, COX7A1, COX7A2, COX7A2L, COX7B, COX7B2, COX7C, COX8A, COX8C, CYC1, CYCS, DNAJC15, MT-ATP6, MT-ATP8, MT-CO1, MT-CO2, MT-CO3, MT-CYB, MT-ND1, MT-ND2, MT-ND3, MT-ND4, MT-ND4L, MT-ND5, MT-ND6, NDUFA1, NDUFA10, NDUFA11, NDUFA12, NDUFA13, NDUFA2, NDUFA3, NDUFA4, NDUFA4L2, NDUFA5, NDUFA6, NDUFA7, NDUFA8, NDUFA9, NDUFAB1, NDUFAF1, NDUFAF2, NDUFAF3, NDUFAF4, NDUFAF5, NDUFAF6, NDUFAF7, NDUFAF8, NDUFB1, NDUFB10, NDUFB11, NDUFB2, NDUFB2-AS1, NDUFB3, NDUFB4, NDUFB5, NDUFB6, NDUFB7, NDUFB8, NDUFB9, NDUFC1, NDUFC2, NDUFS1, NDUFS2, NDUFS3, NDUFS4, NDUFS5, NDUFS6, NDUFS7, NDUFS8, NDUFV1, NDUFV2, NDUFV3, NUBPL, OXA1L, RFESD, SCO1, SCO2, SLC25A4, SURF1, TACO1, TIMMDC1, TMEM126B, TRAP1, TTC19, UQCC1, UQCC2, UQCC3, UQCR10, UQCR11, UQCRB, UQCRC1, UQCRC2, UQCRFS1, UQCRH, UQCRHL, UQCRQ
**Pentose Phosphate**	G6PD, H6PD, PGD, PRPS1, PRPS1L1, PRPS2, RBKS, RGN, RPE, RPIA, TALDO1, TKT, TKTL1, TKTL2
**Peroxisome**	ABCD3, ACAA1, ACOX1, ACOX2, ACOX3, CAT, DDO, DECR2, EHHADH, HAO2, HMGCL, HSDL2, ISOC1, KXD1, PAOX, PEX1, PEX10, PEX11A, PEX12, PEX16, PEX19, PEX26, PEX3, PEX5, PEX6, PEX7, PHYH, PIPOX, PMVK, PXMP2, SCP2, SLC25A17
**Proteasome**	ADRM1, NGLY1, PAAF1, POMP, PSMA1, PSMA2, PSMA3, PSMA3-AS1, PSMA4, PSMA5, PSMA6, PSMA7, PSMA8, PSMB1, PSMB11, PSMB2, PSMB3, PSMB4, PSMB5, PSMB6, PSMB7, PSMC1, PSMC2, PSMC3, PSMC4, PSMC5, PSMC6, PSMD1, PSMD10, PSMD11, PSMD12, PSMD13, PSMD14, PSMD2, PSMD3, PSMD4, PSMD5, PSMD5-AS1, PSMD6, PSMD6-AS2, PSMD7, PSMD8, PSMD9, PSME1, PSME2, PSME3, PSME4, PSMF1, PSMG1, PSMG2, PSMG3, PSMG3-AS1, PSMG4, RAD23B, SHFM1, UBLCP1, UBQLN1, UCHL5, ZFAND2A
**ROS Production**	GPX1, GPX3
**T Cell IL12 Signature**	CCL5, CXCR1, GZMH, IFNG, IL12RB2, IL1RL1, LIFR, TNFSF10, TNFSF13B
**T Cell IL23 Signature**	CCL17, CCL20, CCL22, CCL7, CCR1, CSF2, CXCL2, CXCR5, IL17A, IL17F, IL17RC, IL1R1, IL23R, IL6, ITGA3, PDGFB, TNF
**TCA Cycle**	ACO2, CS, DLAT, DLD, DLST, FH, GLUD1, IDH1, IDH2, IDH3A, IDH3B, IDH3G, MDH2, MPC1, MPC2, OGDH, OGDHL, PDHA1, PDHA2, PDHB, PDHX, PDK1, PDK2, PDK3, PDK4, PDP1, PDP2, PDPR, SDHA, SDHAF1, SDHAF2, SDHAF3, SDHAF4, SDHB, SDHC, SDHD, SUCLA2, SUCLG1, SUCLG2, SUGCT
**Th17**	CCR6, CXCR3, IL17A, IL17F, IL22, IL26, KLRB1, RORA, RORC
**TNF**	ACLY, ACSL1, ADGRE2, AK3, AKAP10, AMPD3, APOL3, ARID3A, ARSE, ASAP1, B4GALT5, BCL2A1, BHLHE41, BHMT, BIRC3, BRCA1, CALD1, CASP1, CASP10, CCL15, CCL20, CCL23, CCL3L1, CD37, CD38, CD83, CDKN3, CKB, CR2, CTNND2, CXCL1, CXCL2, CXCL3, CXCL8, CYP27B1, DAB2, EBI3, EGR1, EGR2, EPB41, EREG, ETAA1, F3, FABP1, FBXL2, FCER2, FCGR2A, FLJ11129, FLNA, G0S2, GBP1, GCH1, GJB2, GLS, GMIP, GP1BA, GRK3, HCAR3, HHEX, HOMER2, HP, ICAM1, IDO1, IFI44, IKBKG, IL16, IL18, IL1A, IL1B, IL1RN, IL6, INHBA, INSIG1, ITGA6, KITLG, KLF1, KMO, LGALS3BP, MAP3K4, MARCKS, MGLL, MMP19, MN1, MRPS15, MSC, MTF1, MX1, NAMPT, NELL2, NFKB1, NFKB2, NFKBIA, NFKBIZ, NKX3-2, NR3C1, OAS3, PATJ, PDE4DIP, PDPN, PIAS4, PLAUR, PTGES, PTGS2, RELB, RPGR, RPS9, SDC4, SERPIND1, SFRP1, SH3BP5, SLAMF1, SLC30A4, SOD2, SPI1, SSPN, STAT4, TAF15, TAP2, TBX3, TFF1, TNF, TNFAIP2, TNFAIP3, TNFRSF11A, TRAF1, TSC22D1, TYROBP, UBE2C, VEGFA, WT1
**Unfolded Protein**	B4GALT3, CALR, CALU, CANX, CDS2, CHST12, CHST2, DERL1, DERL2, DNAJC3, EDEM2, EDEM3, EMC9, ERAP1, ERGIC2, ERO1L, EXT1, GALNT2, GOLT1B, HERPUD1, HYOU1, IER3IP1, IMPAD1, KDELC1, KDELR2, LMAN2, LPGAT1, MAN1A1, MANEA, MANF, NUCB2, PDIA4, PDIA6, PIGK, PPIB, SEC24D, SEC61G, SPCS3, SSR1, SSR3, TRAM1, TRAM2, UGGT1, XBP1
**TGFB Fibroblast**	ABTB2, ACOX1, ACTA2, ACTC, ACTN1, ACTN3, ADAM12, ADAM19, ADAMTS4, ADCY7, AK3, ALS2CR4, AMIGO2, ANGPTL4, AQP1, ARK5, ARL4A, ARNTL, ASE-1, ASNS, ATOH8, ATP10A, ATP1B1, AVP, AXUD1, B4GALT1, BAG3, BFAR, BHLHB2, BLOC1S2, BM039, BMP6, BMPR2, C10orf22, C10orf30, C14orf138, C14orf31, C16orf30, C18orf1, C20orf139, C20orf39, C21orf93, C5orf13, C6orf145, C6orf85, C9orf19, C9orf3, C9orf62, CALM2, CARD4, CBFB, CCDC8, CCL2, CDH2, CDKN2B, CEBPA, CH25H, CHIC2, CHST11, CHST5, CHSY1, CLC, CMKOR1, CNN3, COL4A1, COL4A2, COL5A1, COL5A2, COMP, CREB3L2, CRLF1, CRY1, CSRP1, CSRP2, CTGF, CTPS, CXCL12, CXXC5, CYR61, DACT1, DDIT4, DLC1, DLX2, DNAJB4, DNAJB5, DNAJB9, DOK5L, DSP, DTR, DUSP1, DYRK2, E2F7, EIF4EBP1, ELN, ENC1, ENPP1, EPHB3, ERN1, EYA2, FBXO32, FGF18, FGF2, FGFR3, FGFRL1, FHL2, FLJ10350, FLJ10357, FLJ10378, FLJ14054, FLJ20364, FLJ20366, FLJ20701, FLJ22938, FLJ39370, FLJ45248, FN5, FOXP1, FSTL3, FUS, FZD8, GABRE, GADD45B, GARS, GAS7, GATA6, GDF15, GDF6, GEM, GLS, GNPNAT1, GOPC, GPAM, GPR68, GPT2, GSTT2, HCMOGT-1, HES1, HIF1A, HILS1, HNRPAB, HNRPK, HOMER1, HOXB2, HOXC8, HSPA5, HSPB7, HSXIAPAF1, ID1, ID3, ID4, IER3, IER5L, IGF1, IL11, IL21R, IL4R, IL6, ITR, IVNS1ABP, JUNB, K-ALPHA-1, KCNE4, KCNG1, KCNK1, KCNN4, KCNS3, KCTD11, KIAA0033, KIAA0280, KIAA1102, KIAA1644, KIAA1754, KLF10, KLF13, KLF2, LDHA, LHFPL2, LIF, LIM, LIMK1, LIMK2, LIMS3, LMCD1, LMO4, LOC222171, LOC283824, LOC284454, LOC339047, LOC440502, LOC51333, LRIG1, LRRC8, LTBP2, MAP3K2, MBD4, MGC14376, MGC15476, MGC16121, MGC29875, MGC4504, MGC45871, MGC8685, MGLL, MICAL2, MICAL-L1, MIR100HG, MONDOA, MRC2, MSX1, MTCH1, MTHFD2, NEDD4, NEDD9, NET1, NFATC1, NFYC, NGEF, NID67, NKD2, NLF1, NNMT, NP, NPAS1, NPTX1, NRBF2, NRG1, NUP98, ODC1, P4HA2, P4HA3, PACSIN2, PAWR, PDGFA, PDLIM4, PFKP, PGK1, PGM2L1, PGM3, PHF17, PHLDA2, PHLDB1, PICALM, PIM1, PITX2, PKM2, PLAU, PLAUR, PLEKHA1, PLK3, PLOD2, PNMA1, PODXL, POFUT2, PPP1R13L, PPP1R14C, PPP1R3B, PRICKLE2, PRKAB2, PRO1855, PRPS1, PRPS1L1, PRRX2, PSAT1, PTDSR, PTPNS1, RAI14, RAI17, RASL11B, RGS3, RKHD3, RNF126, RPL21, RPL5, RTTN, RUNX1, RUNX2, RUSC2, S100A16, SAMD11, SARS, SCD, SCHIP1, SDFR1, SERP1, SERPINE1, SERTAD1, SERTAD4, SGCG, SGK, SH3MD1, SIAT4A, SKIL, SLC10A3, SLC16A3, SLC19A2, SLC1A5, SLC20A1, SLC26A1, SLC2A1, SLC38A5, SLC39A14, SLC4A2, SLC7A11, SLC7A5, SMAD7, SMARCB1, SNAI1, SNF1LK, SNX24, SOX4, SOX9, SPARC, SPHK1, SRF, STC2, STCH, STK38L, SYNJ2, SYVN1, TBX3, TD-60, TES, TGFB1, TGFBR1, TGM2, TIMP3, TIPARP, TMEPAI, TMPO, TNC, TNFRSF12A, TNFRSF19L, TPM1, TRIB1, TRIB2, TRIB3, TSK, TUBA3, TUBA6, TUBB2, TUBB3, TUBB4, TUBB6, TUFT1, UAP1, UCK2, UGDH, ULK1, UNC5B, UPP1, USP35, VEGF, VLDLR, VMP1, WNT5B, XBP1, ZNF281, ZNF336, ZNF469, ZNF537

Table S2B: Genes within pathway signatures.

Supplemental Table 2C: Genes within keratinocyte signatures.

Keratinocyte signature	Genes in the signature	Reference
IFNa2a	IFIT1, IFIT2, IFIT3, RSAD2, ISG15, MX1, DDX58, IFI44, PARP9, IFIH1, LAMP3, USP18, IFI44L, IFITM1, STAT1, OAS2, GBP1, PLSCR1, DTX3L, SP110, IFIT5, HERC5, IRF7, CXCL10, DDX60L, HERC6, TRIM21, EPSTI1, MX2, NLRC5, HELZ2, OAS1, DDX60	(*1*)
IFNb	IFIT1, IFIT2, IFIT3, RSAD2, ISG15, MX1, IFI44, PARP9, IFIH1, USP18, LAMP3, CXCL10, OAS2, DDX58, STAT1, IFI44L, GBP1, DTX3L, IFITM1, IFIT5, NLRC5, IRF7, MX2, DDX60L, HERC5, HERC6, TRIM21, XAF1, EPSTI1, PLSCR1, CH25H, PARP12, SP110, SAMD9	(*1*)
IFNg_A	CXCL10, CXCL9, UBD, CXCL11, C1S, HLA-DRA, C1R, HLA-DRB5, PLAAT4, CCL2, RSAD2, HLA-DMA, PSMB9, APOL3, GBP2, CD74, HLA-DRB1, ICAM1, ISG20, BST2, IRF1, CTSS, SERPING1, AIM2, IDO1, APOL1, HLA-DPA1, GLDC, IL32, RARRES1, SECTM1, ETV7, APOL6, IL15, GBP1, IFI35, HLA-DRB4, IFIT3, XAF1, CCL8, CFB, CEACAM1, CFH	(*2*)
IFNg_B	S100A7, S100A8, MMP1, MMP3, KLK7, KRTDAP, KRT10	(*3*)
IFNg_C	CXCL10, PARP9, NLRC5, GBP1, PSMB9, CXCL9, IRF1, DTX3L, PSMB8, IFIT5, TAP1, TRIM69, SERPING1, SECTM1, WARS1, RAD50, INPP1, MCUB, BTN3A3, TLDC2, APOL6, SP110, EDNRA, CASP7, NMI, IFIH1, IFIT3, IFIT2, APOL2, BTN3A2, GBP2, DDX58, STAT1, CACHD1, TRIM21, IRF9	(*1*)
IFNg_D	STAT1, MX2, MX1, ISG15, IRF7, IFI6	(*4*)
IFN	IFIT1, IFIT3, IFIT2, ISG15, RSAD2, MX1, IFI44, PARP9, IFIH1, LAMP3, USP18, STAT1, OAS2, DDX58, DTX3L, IFITM1, IFIT5, IRF7, IFI44L, PLSCR1, HERC6, TRIM21, IRF9, GBP1, PARP12, EIF2AK2, SP110, DDX60L, HERC5, HELZ2, OAS1, ZC3HAV1, EPSTI1, NLRC5, TRIM14	(*1*)
IL1	SERPINB4, SERPINB3, SLC6A14, TGFA, HEPHL1	(*5*)
IL1a	SERPINB4, SERPINB3, SLC6A14, TGFA, HEPHL1	(*5*)
IL1b	NCF2, SAA2, C3, GBP1, IFIT2, IFIT3, IRF1, PRDM1, CFB, CTSC, SERPINB1, SERPINB3, SERPINB4, MMP9, PLAT, SERPINA3, PSMB9, CCL20, CCL5, CXCL1, CXCL2, CXCL3, TYMP, EDN1, IL1A, IL1B, IL36G, CXCL8, INHBA, TGFA, TNFAIP2, MPZL2, NINJ1, IL32, TNFAIP6, CLDN4, KRT34, S100A12, S100A7, S100A8, S100A9, SPRR2B, PDZK1IP1, RHCG, PTP4A3, LYN, PLAU, SGK1, HCAR3, IFNGR1, IL1RN, HBEGF, PLAUR, PPIF, NDRG2, NFKB1, NFKB2, NFKBIA, NFKBIE, RELB, WNT4, NOD2, RIPK2, TNFSF10, BIRC3, IER3, TNFAIP3, PPP1R15A, RCAN1, BHLH340, MAFF, REL, TNIP1, ZFP36, ALDH1A3, APOBEC3A, OAS3, EPHX3, CYB5R2, SOD2, TAPBP, ATP1B1, C1QTNF1, LCN2, SLC11A2, SLC6A14, TFRC	(*6*)
IL17_A	SPRR2A, DEFB4A, IL36G, SPRR2C, SPRR2E, SPRR2F, NFKBIZ, ZC3H12A, PI3, LCN2, SERPINB4, SPRR2B, CXCL8, S100A8, S100A9, SPRR2D, NDRG2, SAA1, RHCG, IL1B, ATP1B1, IER3, CCL20, PDZK1IP1, ALDH1A3, CXCL2, C15orf48, NOD2, ALOX12B, ARHGEF37, SOX7, SLC11A2, ANKK1, NFKBIA, GJB6, TYMP, SAT1, MAFF, DNER, ISG20L2, RBM14, F2R, ACTA2, TNNI2, CPA4	(*7*)
IL17_B	CXCL1, CXCL2, CXCL5, CXCL6, CXCL8, CXCL9, CXCL10, CXCL11, CCL2, CCL5, CCL7, CCL11, CXCL12, CCL20, IL6, IL19, CSF2, CSF3, ICAM1, PTGS2, NOS2, LCN2, DEFB4A, S100A7, S100A8, S100A9, MUC5AC, MUC5B, EREG, SOCS3, TNFSF11, MMP1, MMP3, MMP9, MMP13, TIMP1, ADAMTS4, CEBPB, CEBPD, NFKBIZ	(*8*)
IL17_C	DEFB4A, S100A7, S100A12, CCL20, SERPINB4, CXCL6, PDZK1IP1, SAA2, SPRR2C, ZC3H12A, IL36G, CXCL8, SLC6A14, CXCL1, CXCL2, RHCG, CXCL5, NDRG2, NOD2, LCN2, PI3, C3, CXCL3, MAP3K8, SPRR2D, IL1B, CRABP2, NMB, SLC11A2, S100A9, SLC25A12, TFRC, NAV3, CYB5R2, IER3, SAT1, RPS6KA5, NAMPT, S100A8, ALDH1A3, TYW1	(*2*)
IL17a_D	C1orf68, LY6G6C, LCE1B, NIPAL4, SLC46A2, SERPINB4, SERPINB3, SLC6A14, TGFA, HEPHL1	(*5*)
IL17_TNF	IL19, CXCL8, IL23A, CCL20, IL6, CXCL1, TNF, IL17C, CXCL5, DEFB4A, CFB, S100A7A, S100A7, PLAT, IL36G, CXCL6, IL1B, IL36RN, TNFSF18, PI3, LCN2, S100A9, MAP3K8, S100P, S100A8	(*7*)
IL17a_TNF	CNFN, TREX2, SPRR2B, SPRR2D, SULT2B1	(*5*)
IL17c_3hr	CSF3, CXCL8, CXCL1, CXCL2, CXCL3, IL1B, IL36G, CCL2, CCL20, IL17D, NFKBID, NFKBIZ, TNFAIP6, IL1RL1, MMP12	(*9*)
IL17c_24hr	CSF3, CXCL1, IL1B, IL36G, CCL20, IL17D, C3, NFKBID, NFKBIZ, TNFAIP6, TNIP3, IL1RL1, MMP12, SAA4, VNN3, S100A7, S100A7A, S100A8, S100A9, DEFB1, DEFB4A	(*9*)
IL22_A	S100A7, SERPINB4, S100P, SERPINB1, CARHSP1, HRH1, CYP27B1, TM4SF1, CASP4, RAB27A, IL13RA1	(*2*)
IL22_B	S100A7, S100A8, MMP1, MMP3	(*3*)
IL4_IL13	HSD3B1, NTRK1, CLDN5, CCL26, NNMT, LOXL4, TNC, SERPINB3, SERPINB4, PDZK1IP1, KCNJ12, CA2, HSD3B2, RFLNA, IL33, MGAM, ST8SIA1, CXCL6, ANOS1, SIDT1, CAPN14, CISH, CYP1B1, TOX, P2RY1, MYH15, ID3, LSP1, CSF3, GJB2, GKN1, LRRC8B, TREML2, OGDHL, CTSC, PALMD, GUCA1C, SLCO2A1, BRINP1, E2F2, MYB, NPNT, NR3C2, PPFIBP2, EDNRA, GDPD2, SERPINB13, HAS3, BATF3, MMP7, CPM, APOL3, RGS16, MEST, MYOZ2, CENPV, STAR, KIF24, CRABP2, GJB6, TMEM45B, TENM2, MAP2K6, PCDH7, NRG1, SLC5A12, KLRG1, CFH, GLS2, KITLG, SLC7A4, BTBD3, NMU, POU2F3, RASL11B, KRT24, KLHL23, TF, HPDL, SLC2A5, MAN1C1, ZNF367, CFHR1, KIRREL3, NEFH, MMP12, DUSP19, PHGDH, VSNL1, LRFN2, KIAA1958, JADE1, DUOX1, FABP6, KCNRG, SEMA3A, PNPT1, C10orf82, RTP5, UFD1, NABP1, NPW, IL7R, TP73, HLA-DOA, CTRL, DESI2, RTKN2, FOLH1, SIPA1L2, DUSP13, SLC15A2, FJX1, IL15RA, GATA3, MAP3K14, KRT8, KRT18P45, TP53AIP1, SHF, TPK1, HSPD1, FZD10-AS1, ZNF697, CRACR2A, BLMH, SLC35G1, PRDM13, ANO1, CYP27B1, SNX5, LYAR, LDLRAD3, GPD1L, ELAVL2, MAL2, GAS2, PBX1, CD302, DEPDC4, ITPR1, SLIRP, SCN2B, NUP107, KRT18P32, RBM47, KRT18P28, SLC39A8, KRT18P20, CENPL, MESP1, SLC26A2, POLR1B, PTPN4, HS6ST2, RAE1, CFD, COBLL1, CHAC2, EAF2, PLA2G4A, CD47, NOX1	(*10*)
TGFa_30min	EREG, VEGFA, HBEGF, IL1B, IL6, IL1A, CXCL3, CXCL2, CXCL8, EDN1, ZYX, DUSP1, PTGS2, DUSP6, ARHGEF2, PHLDA1, JUN, EGR1, FOS, CITED2, DDIT3, DUSP4, PPP1R15A, TNFAIP3, NOP14-AS1	(*11*)
TGFa_60min	ARTN, VEGFA, HBEGF, IL1B, LIF, IL11, IL1A, IL36G, SERPINE1, FSTL3, SLPI, PI3, THBD, CLDN4, EPHA2, PLAUR, JMJD6, YRDC, RHCG, ULBP2, SPRY4, GNE, PLEKHG3, PTPRE, TUBB2A, TUBB2B, PKP1, MAT2A, PTGS2, HS3ST1, FUT1, PLK3, FBXL18, SLC20A1, ALAS1, ISG20L2, SPRR2B, NLRP1, GRPEL1, CAPN3, SPRR1A, IVL, PHLDA1, FOSL1, EGR3, ID1, FOS, ZFP36L2, CDKN1A, IER2, DDIT4, COA1, KDM6B, INAVA, LGALSL, LRRC8E, AKIP1, NUAK2, C1orf56, PDXDC1	(*11*)
TGFa_120min	EREG, IL1B, IL36G, CXCL2, CXCL8, MMP1, MMP10, PTP4A1, MAT2A, CYP27B1, SCG5, PHLDA1, ARL4C	(*11*)
TNF_A	CNFN, TREX2, SPRR2B, SPRR2D, SULT2B1	(*5*)
TNFa_B	MMP9, C1QTNF1, IL36G, TNFAIP3, CXCL8, IRAK2, CXCL10, TYMP, IL1B, SOD2, NFKBIA, S100A9, HBEGF, PLAU, HLA-B, MMP10, PI3, BIRC3, KREMEN2, WNT4, ISG15, PRDM1, TNFSF10, IL1RN, TNIP1, SERPINB2, IRF1, S100A8, DRAM1, LYN, NFKBIZ, CCL27, C1orf74, MUC1, ELF3, IFIT3, RELB, RHCG, DUSP1, C1R, CSF2, TNFRSF6B, CXCL16, PIK3IP1	(*7*)
TNFa_C	BIRC3, ZC3H12C, CXCL10, NINJ1, LACC1, PPP1R3C, RELB, CPNE8, COL12A1, BID, PLAU, STEAP4, DDX58, DRAM1, BDKRB1, GCH1, SDC4, IFIH1, TNFAIP3, TAP1, BCL3, SRCAP, CCL20, CYLD, SOD2, PSMB8, TNFRSF10B, FZD8, FAS, PSTPIP2, TSLP, MMP28	(*1*)
TNFa_early	CEP135, EFNA1, MARCKSL1, ICAM1, NINJ1, CFLAR, CFB, IVL, S100A8, S100A9, SPRR2A, TGM1, NEFM, TPST1, H1-10, IFIT3, KRT34, PDPN, SCO2, MMP10, MMP9, RAB32, TNFAIP2, CCL20, CCL27, CCL5, CXCL10, CXCL11, CXCL1, CXCL2, CXCL3, CXCL8, BMP2, IL1RN, LTB, IL32, TNF, TNFSF10, VEGFC, IRF1, NFKBIA, SOX4, TNFAIP3, TAP1, ATP12A	(*12*)
TNFa_late	KYNU, TNC, TRAF1, PSME2, PSMB10, PSMB9, BTG3, C1R, C1S, NEDD9, CSPG4, COL16A1, GALT, H2AC18, H2AC20, HLA-F, HLA-A, MR1, SAA1, OAS1, OASL, CD47, ITGB6, CDH3, KRT2, LYN, ACOX1, CD58, PDZK1IP1, GPD2, SOD2, PML, DUSP1, ALOX12B, GGH, MMP13, WFDC2, SPINK5, TLR1, NCK1, BST2, TYMP, LSS, HIVEP1, LITAF, SMAD1, ZFYVE9, MEOX1, RELB, SLC39A8, DLC1	(*12*)
TNFa_IFNg	IL32, CCL8, CXCL3, CXCL8, CCL20, CXCL5, CCL17, CCL2, CXCL14, CCL5, CCL22, IFNL1, IFNB1, IFNL2, IFNL3, IL6, IL1B, CCL4, TNF, LTB, IL36G, IL33, CSF2, CXCL1, IL24, CCL7, IL11, IFNE, CXCL16, IL16	(*13*)

References:

1. Y. Yao, L. Richman, C. Morehouse, M. de los Reyes, B. W. Higgs, A. Boutrin, B. White, A. Coyle, J. Krueger, P. A. Kiener, B. Jallal, Type I interferon: Potential therapeutic target for psoriasis?, *PLoS One* **3** (2008), doi:10.1371/journal.pone.0002737.

2. K. E. Nograles, L. C. Zaba, E. Guttman-Yassky, J. Fuentes-Duculan, M. Suárez-Fariñas, I. Cardinale, A. Khatcherian, J. Gonzalez, K. C. Pierson, T. R. White, C. Pensabene, I. Coats, I. Novitskaya, M. A. Lowes, J. G. Krueger, Th17 cytokines interleukin (IL)-17 and IL-22 modulate distinct inflammatory and keratinocyte-response pathways, *Br. J. Dermatol.* **159**, 1092–1102 (2008).

3. K. Wolk, E. Witte, E. Wallace, W. D. Döcke, S. Kunz, K. Asadullah, H. D. Volk, W. Sterry, R. Sabat, IL-22 regulates the expression of genes responsible for antimicrobial defense, cellular differentiation, and mobility in keratinocytes: A potential role in psoriasis, *Eur. J. Immunol.* **36**, 1309–1323 (2006).

4. R. Mansourian, D. M. Mutch, N. Antille, J. Aubert, P. Fogel, J. M. Le Goff, J. Moulin, A. Petrov, A. Rytz, J. J. Voegel, M. A. Roberts, The Global Error Assessment (GEA) model for the selection of differentially expressed genes in microarray data, *Bioinformatics* **20**, 2726–2737 (2004).

5. B. Li, L. C. Tsoi, W. R. Swindell, J. E. Gudjonsson, T. Tejasvi, A. Johnston, J. Ding, P. E. Stuart, X. Xing, J. J. Kochkodan, J. J. Voorhees, H. M. Kang, R. P. Nair, G. R. Abecasis, J. T. Elder, Transcriptome analysis of psoriasis in a large case-control sample: RNA-seq provides insights into disease mechanisms, *J. Invest. Dermatol.* **134**, 1828–1838 (2014).

6. S. Yano, T. Banno, R. Walsh, M. Blumenberg, Transcriptional responses of human epidermal keratinocytes to cytokine interleukin-1, *J. Cell. Physiol.* **214**, 1–13 (2008).

7. A. Chiricozzi, E. Guttman-Yassky, M. Suárez-Farĩas, K. E. Nograles, S. Tian, I. Cardinale, S. Chimenti, J. G. Krueger, Integrative responses to IL-17 and TNF-α in human keratinocytes account for key inflammatory pathogenic circuits in psoriasis, *J. Invest. Dermatol.* **131**, 677–687 (2011).

8. F. Shen, S. L. Gaffen, Structure-function relationships in the IL-17 receptor: Implications for signal transduction and therapy*Cytokine* **41**, 92–104 (2008).

9. V. Ramirez-Carrozzi, A. Sambandam, E. Luis, Z. Lin, S. Jeet, J. Lesch, J. Hackney, J. Kim, M. Zhou, J. Lai, Z. Modrusan, T. Sai, W. Lee, M. Xu, P. Caplazi, L. Diehl, J. De Voss, M. Balazs, L. Gonzalez, H. Singh, W. Ouyang, R. Pappu, IL-17C regulates the innate immune function of epithelial cells in an autocrine manner, *Nat. Immunol.* **12**, 1159–1166 (2011).

10. S. Hirakawa, R. Saito, H. Ohara, R. Okuyama, S. Aiba, Dual Oxidase 1 Induced by Th2 Cytokines Promotes STAT6 Phosphorylation via Oxidative Inactivation of Protein Tyrosine Phosphatase 1B in Human Epidermal Keratinocytes, J. Immunol. 186, 4762–4770 (2011).

11. C. F. Cheng, J. Fan, B. Bandyopahdhay, D. Mock, S. Guan, M. Chen, D. T. Woodley, W. Li, Profiling motility signal-specific genes in primary human keratinocytes, *J. Invest. Dermatol.* **128**, 1981–1990 (2008).

12. T. Banno, A. Gazel, M. Blumenberg, Pathway-specific profiling identifies the NF-κB-dependent tumor necrosis factor α-regulated genes in epidermal keratinocytes, *J. Biol. Chem.* **280**, 18973–18980 (2005).

13. H. Fujita, The role of IL-22 and Th22 cells in human skin diseases, *J. Dermatol. Sci.* **72**, 3–8 (2013).

Table S2C: Genes within keratinocyte signatures.

Supplemental Table 2D: Genes within T cell signatures.

T cell Signature	Genes in the signature	Reference
Dermal Aner/Act T Cell	CD160, CTLA4, ICOS, KLRG1, LAG3, PDCD1	(*1-5*)
Dermal CD8 T Cell	CD8A, CD8B	
Dermal Tfh	BTLA, IL21, SH2D1A	
Dermal Th1	CCL5, CXCR3, EOMES, IFNG, PRF1, TBX21, GZMK	
Dermal Th17	CCR6, IL12RB1, IL17A, IL17F, IL22, IL23R, IL26, KLRB1, RORC	
Dermal Th2	GATA3, IL13, IL4, IL4R, IL5	
Dermal Treg	FOXP3, IKZF2, TNFRSF9	

References:

1. S. Crotty, Follicular helper CD4 T cells (TFH), *Annu Rev Immunol*. **29**, 621–663 (2011), doi: 10.1146/annurev-immunol-031210-101400.

2. T. Duhen, C. Ni, D. Campbell, Identification of a specific gene signature in human Th1/17 cells (BA13P.126), *J. Immunol.* **192**, 177.12 LP-- 177.12 (2014).

3. N. Kutukculer, E. Azarsiz, G. Aksu, N. E. Karaca, CD4+CD25+Foxp3+ T regulatory cells, Th1 (CCR5, IL-2, IFN-γ) and Th2 (CCR4, IL-4, Il-13) type chemokine receptors and intracellular cytokines in children with common variable immunodeficiency, *Int. J. Immunopathol. Pharmacol.* **29**, 241–251 (2016).

4. J. B. Wing, Y. Kitagawa, M. Locci, H. Hume, C. Tay, T. Morita, Y. Kidani, K. Matsuda, T. Inoue, T. Kurosaki, S. Crotty, C. Coban, N. Ohkura, S. Sakaguchi, A distinct subpopulation of CD25− T-follicular regulatory cells localizes in the germinal centers, *Proc. Natl. Acad. Sci. U. S. A.* **114**, E6400–E6409 (2017).

5. B. Höllbacher, T. Duhen, S. Motley, M. M. Klicznik, I. K. Gratz, D. J. Campbell, Transcriptomic profiling of human effector and regulatory T cell subsets identifies predictive population signatures, *Immunohorizons* **4**, 585–596 (2021).

Table S2D: Genes within T cell signatures.

Supplemental Table 3A: Class balance stratagy used for machine learning classification.

Skin Type	Comparison		Original Samples	ML Samples	Class balance stratagy	Class		Greedy Elimination
Lesional Skin	(A)	DLE vs CTL	90 DLE vs 164 CTL	90 DLE vs 164 CTL	None	Class 0	CTL	IL12, Monocyte / Myeloid Cell
						Class 1	DLE	
	(B)	AD vs CTL	132 AD vs 164 CTL	132 AD vs 164 CTL	None	Class 0	CTL	Monocyte, IL12, Peroxisome
						Class 1	AD	
	(C)	SSc vs CTL	97 SSc vs 164 CTL	97 SSc vs 97CTL	Random Undersampling	Class 0	CTL	Monocyte, IL12, Complement Proteins, Peroxisomes, Monocyte /Myeloid Cell
						Class 1	SSc	
	(D)	PSO vs CTL	183 PSO vs 164 CTL	183 PSO vs 164 CTL	None	Class 0	CTL	IL12, Monocyte
						Class 1	PSO	
	(E)	DLE vs SUB	90 DLE vs 54 SUB	90 DLE vs 90 SUB	Random Oversample	Class 0	SUB	None
						Class 1	DLE	
	(F)	DLE vs PSO	90 DLE vs 183 PSO	90 DLE vs 110 PSO	Excluded GSE117468	Class 0	PSO	None
						Class 1	DLE	
	(G)	DLE vs AD	90 DLE vs 132 AD	90 DLE vs 132 AD	None	Class 0	AD	Monocyte / Myeloid Cell, TNF
						Class 1	DLE	
	(H)	DLE vs SSc	90 DLE vs 97 SSc	90 DLE vs 97 SSc	None	Class 0	SSc	FABO, TNF
						Class 1	DLE	
Nonlesional Skin	(I)	DLE vs CTL	21 DLE vs 164 CTL	98 DLE vs 122 CTL	SMOTE over and undersample	Class 0	CTL	None
						Class 1	DLE	
	(J)	PSO vs CTL	163 PSO vs 164 CTL	163 PSO vs 164 CTL	Excluded PSO from GSE52471 and GSE109248	Class 0	CTL	Monocyte
						Class 1	PSO	
	(K)	AD vs CTL	132 AD vs 164 CTL	164 AD vs 164 CTL	Random Oversample	Class 0	CTL	Monocyte
						Class 1	AD	
	(L)	DLE vs PSO	21 DLE vs 163 PSO	97 DLE vs 121 PSO	SMOTE over and undersample	Class 0	PSO	Monocyte, FABO, IL12
						Class 1	DLE	
	(M)	DLE vs AD	21 DLE vs 132 AD	79 DLE vs 98 AD	SMOTE over and undersample	Class 0	AD	Monocyte, T Cell, FABO,IL12
						Class 1	DLE	
	(N)	PSO vs AD	163 PSO vs 132 AD	163 PSO vs 132 AD	None	Class 0	AD	Monocyte/Myeloid Cell, Complement Proteins, Peroxisome, IL12
						Class 1	PSO	

Table S3A: Class balance strategy used for machine learning classification.

Supplemental Table 3B: Number of samples pooled from each skin dataset to create input for machine learning.

	Samples	Datasets	Number of samples
Lesional samples	90 DLE	GSE52471	7
		GSE72535	9
		GSE81071_A	26
		GSE81071_B	21
		GSE109248	6
		GSE100093	15
		GSE120809	6
	132 AD	GSE130588	19
		GSE137430	38
		GSE157194	54
		GSE121212	21
	183 PSO	GSE52471	3
		GSE109248	17
		GSE117239	63
		GSE117468	73
		GSE121212	27
	97 SSc	GSE58095	39
		GSE130955	58

	Samples	Datasets	Number of samples
Non Lesional samples	21 DLE	GSE100093	15
		GSE120809	6
	132 AD	GSE130588	19
		GSE137430	38
		GSE157194	54
		GSE121212	21
	163 PSO	GSE117239	63
		GSE117468	73
		GSE121212	27

	Samples	Datasets	Number of samples
Healthy control samples	164 CTL	GSE52471	3
		GSE72535	8
		GSE81071_A	7
		GSE81071_B	6
		GSE109248	13
		GSE130588	20
		GSE121212	38
		GSE58065	36
		GSE130955	33

Table S3B: Number of samples pooled from each skin dataset to create input for machine learning.

Supplemental Table 4: Comparison between mean of Z-score per gene signature and Z-score GSVA enrichment of nonlesional skin samples.

		P value Welch's T test						Hedges' g Effect Size					
		NL DLE and CTL		NL PSO and CTL		NL ATD and CTL		NL DLE and CTL		NL PSO and CTL		NL ATD and CTL	
**Gene Signature**	**# of genes**	**Z-score mean**	**GSVA score**	**Z-score mean**	**GSVA score**	**Z-score mean**	**GSVA score**	**Z-score mean**	**GSVA score**	**Z-score mean**	**GSVA score**	**Z-score mean**	**GSVA score**
AA Metabolism	12	**	*	*	**	*	ns	0.85	0.44	0.25	0.35	-0.23	0.17
Anti inflammation	3	ns	ns	ns	ns	ns	*	-0.12	-0.12	0.00	0.16	0.07	0.25
Apoptosis	13	*	ns	****	****	****	****	0.24	0.15	-0.89	-1.08	-1.08	-1.23
B Cell	4	**	***	ns	ns	ns	ns	0.72	0.95	0.08	0.10	-0.17	-0.08
Cell Cycle	18	ns	ns	****	ns	****	ns	-0.01	-0.19	0.88	-0.01	0.46	0.14
Complement Proteins	7	**	****	ns	*	****	****	0.38	0.45	-0.10	0.24	-1.87	-1.85
Endothelial Cell	11	****	****	****	****	****	****	-1.79	-1.71	-1.16	-0.61	-0.84	-0.93
FAAO	4	****	****	****	****	**	**	1.46	1.47	1.72	1.67	-0.35	-0.39
FABO	44	*	**	****	***	**	ns	1.09	0.92	-0.53	-0.36	-0.36	-0.13
Fibroblast	63	****	****	****	***	****	****	1.64	1.00	1.33	0.43	1.73	1.35
Glycolysis	20	*	**	****	****	ns	*	-0.86	-0.82	-2.07	-1.83	0.08	0.24
Granulocyte	2	****	****	****	*	****	****	-1.26	-1.44	-0.67	-0.23	-0.60	-0.74
IFN	21	ns	ns	****	****	****	****	0.39	-0.04	-0.63	-0.63	-1.53	-0.69
IL12	36	****	****	****	****	ns	*	-1.54	-2.16	-0.99	-0.63	0.13	0.26
IL17 Complex	4	*	****	****	****	*	**	0.46	0.87	0.84	0.89	0.26	0.36
Inflammasome	12	****	****	ns	ns	****	****	-0.78	-1.20	0.02	-0.17	-0.66	-0.83
Keratinocyte	57	ns	ns	***	*	ns	ns	0.74	0.62	-0.40	-0.26	-0.18	-0.09
Langerhans Cell	4	****	****	****	****	ns	ns	-1.44	-1.37	-1.19	-1.31	-0.16	-0.21
Melanocyte	8	****	**	****	****	****	*	0.91	0.62	0.68	0.62	0.75	0.28
Monocyte	8	ns	ns	ns	ns	ns	ns	-0.19	-0.21	0.10	0.14	0.08	-0.03
Monocyte/Myeloid Cell	40	****	****	****	****	ns	ns	0.67	0.71	1.24	1.15	-0.15	-0.12
Neutrophil	5	****	**	****	****	****	****	0.62	-0.40	0.68	0.79	0.68	0.87
NK Cell	2	ns	*	****	****	****	****	0.18	0.56	1.16	1.13	1.69	1.79
OXPHOS	74	ns	ns	****	****	ns	ns	0.49	0.12	-0.94	-1.15	0.02	0.15
pDC	3	ns	*	****	****	ns	ns	-0.74	-0.87	-1.89	-1.79	-0.05	-0.01
Pentose Phosphate	9	**	**	****	****	****	****	-0.91	-1.14	-1.17	-1.18	1.48	0.95
Peroxisome	25	****	****	****	**	ns	ns	1.36	1.12	1.31	0.31	0.13	-0.01
Plasma Cell	10	****	***	****	**	****	****	0.79	0.58	0.62	0.36	0.72	0.49
Platelet	3	****	****	****	****	****	****	-1.18	-0.89	-0.86	-0.44	-0.61	-0.66
Proteasome	41	ns	ns	****	****	*	ns	-0.17	-0.32	-1.13	-0.98	0.29	0.15
ROS Production	2	ns	*	ns	****	****	****	0.47	0.75	0.16	0.77	1.17	1.64
Skin-specific DC	3	ns	***	****	*	****	****	0.17	-0.92	1.00	0.24	2.03	1.79
T Cell	9	ns	ns	****	****	**	ns	-0.20	-0.10	0.78	0.66	-0.35	-0.08
T Cell IL12 Signature	3	****	****	****	****	ns	ns	-0.89	-0.78	-1.00	-0.71	0.08	0.19
T Cell IL23 Signature	7	*	ns	ns	ns	**	****	-0.46	0.31	-0.11	-0.16	-0.32	0.46
TCA Cycle	32	ns	ns	****	****	***	****	0.34	0.73	-1.32	-0.72	0.39	0.75
Th17	2	**	ns	ns	***	***	****	-0.34	-0.09	-0.18	0.41	0.39	0.74
TNF	86	****	****	ns	*	****	****	-1.02	-1.09	-0.08	-0.26	0.72	0.55
TGFB Fibroblast	229	ns	ns	****	****	ns	ns	0.24	0.10	-0.84	-0.63	0.03	0.22
Unfolded Protein	40	ns	ns	****	****	ns	*	-0.21	-0.24	-0.90	-0.65	-0.08	0.24

Table S4: Comparison between mean of Z-score per gene signature and GSVA enrichment scores using Z-score transformation of nonlesional samples. Left indicates statistics from Welch's t-test between NL and CTL samples. Right indicates statistics from Hedges' g Effect size of NL compared to CTL samples.

REFERENCES AND NOTES

1. B. Tebbe, C. Orfanos, Epidemiology and socioeconomic impact of skin disease in lupus erythematosus. *Lupus* **6**, 96–104 (1997).

2. M. P. Maz, J. Michelle Kahlenberg, Cutaneous and systemic connections in lupus. *Curr. Opin. Rheumatol.* **32**, 583–589 (2020).

3. L. Uva, D. Miguel, C. Pinheiro, J. P. Freitas, M. Marques Gomes, P. Filipe, Cutaneous manifestations of systemic lupus erythematosus. *Autoimmune Dis.* **2012**, 834291 (2012).

4. J. Wenzel, Cutaneous lupus erythematosus: New insights into pathogenesis and therapeutic strategies. *Nat. Rev. Rheumatol.* **15**, 519–532 (2019).

5. S. Ribero, S. Sciascia, L. Borradori, D. Lipsker, The cutaneous spectrum of lupus erythematosus. *Clin. Rev. Allergy Immunol.* **53**, 291–305 (2017).

6. P. Vashisht, K. Borghoff, J. R. O'Dell, M. Hearth-Holmes, Belimumab for the treatment of recalcitrant cutaneous lupus. *Lupus* **26**, 857–864 (2017).

7. E. F. Morand, R. Furie, Y. Tanaka, I. N. Bruce, A. D. Askanase, C. Richez, S.-C. Bae, P. Z. Brohawn, L. Pineda, A. Berglind, R. Tummala, Trial of anifrolumab in active systemic lupus erythematosus. *N. Engl. J. Med.* **382**, 211–221 (2020).

8. A. Menter, B. E. Strober, D. H. Kaplan, D. Kivelevitch, E. F. Prater, B. Stoff, A. W. Armstrong, C. Connor, K. M. Cordoro, D. M. R. Davis, B. E. Elewski, J. M. Gelfand, K. B. Gordon, A. B. Gottlieb, A. Kavanaugh, M. Kiselica, N. J. Korman, D. Kroshinsky, M. Lebwohl, C. L. Leonardi, J. Lichten, H. W. Lim, N. N. Mehta, A. S. Paller, S. L. Parra, A. L. Pathy, R. N. Rupani, M. Siegel, E. B. Wong, J. J. Wu, V. Hariharan, C. A. Elmets, Joint AAD-NPF guidelines of care for the management and treatment of psoriasis with biologics. *J. Am. Acad. Dermatol.* **80**, 1029–1072 (2019).

9. D. Deleanu, I. Nedelea, Biological therapies for atopic dermatitis: An update. *Exp. Ther. Med.* **17**, 1061–1067 (2019).

10. A. Jabbari, M. Suárez-Fariñas, J. Fuentes-Duculan, J. Gonzalez, I. Cueto, A. G. Franks, J. G. Krueger, Dominant Th1 and minimal Th17 skewing in discoid lupus revealed by transcriptomic comparison with psoriasis. *J. Invest. Dermatol.* **134**, 87–95 (2014).

11. B. F. Chong, L. C. Tseng, G. A. Hosler, N. M. Teske, S. Zhang, D. R. Karp, N. J. Olsen, C. Mohan, A subset of CD163$^+$ macrophages displays mixed polarizations in discoid lupus skin. *Arthritis Res. Ther.* **17**, 324 (2015).

12. A. M. S. Barron, J. C. Mantero, J. D. Ho, B. Nazari, K. L. Horback, J. Bhawan, R. Lafyatis, C. Lam, J. L. Browning, Perivascular adventitial fibroblast specialization accompanies T Cell retention in the inflamed human dermis. *J. Immunol.* **202**, 56–68 (2019).

13. P. Mande, B. Zirak, W. C. Ko, K. Taravati, K. L. Bride, T. Y. Brodeur, A. Deng, K. Dresser, Z. Jiang, R. Ettinger, K. A. Fitzgerald, M. D. Rosenblum, J. E. Harris, A. Marshak-Rothstein, Fas ligand promotes an inducible TLR-dependent model of cutaneous lupus–like inflammation. *J. Clin. Invest.* **128**, 2966–2978 (2018).

14. J. Liu, C. C. Berthier, J. M. Kahlenberg, Enhanced inflammasome activity in systemic lupus erythematosus is mediated via type I interferon–induced up-regulation of interferon regulatory factor 1. *Arthritis Rheumatol.* **69**, 1840–1849 (2017).

15. L. C. Tsoi, G. A. Hile, C. C. Berthier, M. K. Sarkar, T. J. Reed, J. Liu, R. Uppala, M. Patrick, K. Raja, X. Xing, E. Xing, K. He, J. E. Gudjonsson, J. M. Kahlenberg, Hypersensitive IFN responses in lupus keratinocytes reveal key mechanistic determinants in cutaneous lupus. *J. Immunol.* **202**, 2121–2130 (2019).

16. V. P. Werth, D. Fiorentino, B. A. Sullivan, M. J. Boedigheimer, K. Chiu, C. Wang, G. E. Arnold, M. A. Damore, J. Bigler, A. A. Welcher, C. B. Russell, D. A. Martin, J. B. Chung, Brief Report: Pharmacodynamics, safety, and clinical efficacy of AMG 811, a human anti–interferon-γ antibody, in patients with discoid lupus erythematosus. *Arthritis Rheumatol.* **69**, 1028–1034 (2017).

17. M. D. Catalina, P. Bachali, N. S. Geraci, A. C. Grammer, P. E. Lipsky, Gene expression analysis delineates the potential roles of multiple interferons in systemic lupus erythematosus. *Commun. Biol.* **2**, 140 (2019).

18. K. E. Nograles, L. C. Zaba, E. Guttman-Yassky, J. Fuentes-Duculan, M. Suárez-Fariñas, I. Cardinale, A. Khatcherian, J. Gonzalez, K. C. Pierson, T. R. White, C. Pensabene, I. Coats, I. Novitskaya, M. A. Lowes, J. G. Krueger, Th17 cytokines interleukin (IL)-17 and IL-22 modulate distinct inflammatory and keratinocyte-response pathways. *Br. J. Dermatol.* **159**, 1092–1102 (2008).

19. Y. Asano, Systemic sclerosis. *J. Dermatol.* **45**, 128–138 (2018).

20. P. M. Brunner, E. Guttman-Yassky, D. Y. M. Leung, The immunology of atopic dermatitis and its reversibility with broad-spectrum and targeted therapies. *J. Allergy Clin. Immunol.* **139**, S65–S76 (2017).

21. K. M. Kingsmore, P. Bachali, M. D. Catalina, A. R. Daamen, S. E. Heuer, R. D. Robl, A. C. Grammer, P. E. Lipsky, Altered expression of genes controlling metabolism characterizes the tissue response to immune injury in lupus. *Sci. Rep.* **11**, 14789 (2021).

22. S. J. Waddell, S. J. Popper, K. H. Rubins, M. J. Griffiths, P. O. Brown, M. Levin, D. A. Relman, Dissecting interferon-induced transcriptional programs in human peripheral blood cells. *PLOS ONE* **5**, e9753 (2010).

23. J. L. Sargent, A. Milano, S. Bhattacharyya, J. Varga, M. K. Connolly, H. Y. Chang, M. L. Whitfield, A TGFB-responsive gene signature is associated with a subset of diffuse scleroderma with increased disease severity. *J. Invest. Dermatol.* **130**, 694–705 (2010).

24. C. L. Langrish, Y. Chen, W. M. Blumenschein, J. Mattson, B. Basham, J. D. Sedgwick, T. McClanahan, R. A. Kastelein, D. J. Cua, IL-23 drives a pathogenic T cell population that induces autoimmune inflammation. *J. Exp. Med.* **201**, 233–240 (2005).

25. A. Blauvelt, A. Chiricozzi, The immunologic role of IL-17 in psoriasis and psoriatic arthritis pathogenesis. *Clin. Rev. Allergy Immunol.* **55**, 379–390 (2018).

26. J. E. Hawkes, B. Y. Yan, T. C. Chan, J. G. Krueger, Discovery of the IL-23/IL-17 signaling pathway and the treatment of psoriasis. *J. Immunol.* **201**, 1605–1613 (2018).

27. M. D. Catalina, P. Bachali, A. E. Yeo, N. S. Geraci, M. A. Petri, A. C. Grammer, P. E. Lipsky, Patient ancestry significantly contributes to molecular heterogeneity of systemic lupus erythematosus. *JCI Insight* **5**, e140380 (2020).

28. T. Vazquez, R. Feng, K. J. Williams, V. P. Werth, Immunological and clinical heterogeneity in cutaneous lupus erythematosus. *Br. J. Dermatol.* **185**, 481–483 (2021).

29. J. L. Zhu, L. T. Tran, M. Smith, F. Zheng, L. Cai, J. A. James, J. M. Guthridge, B. F. Chong, Modular gene analysis reveals distinct molecular signatures for subsets of patients with cutaneous lupus erythematosus. *Br. J. Dermatol.* **185**, 563–572 (2021).

30. L. C. Tsoi, E. Rodriguez, F. Degenhardt, H. Baurecht, U. Wehkamp, N. Volks, S. Szymczak, W. R. Swindell, M. K. Sarkar, K. Raja, S. Shao, M. Patrick, Y. Gao, R. Uppala, B. E. Perez White, S. Getsios, P. W. Harms, E. Maverakis, J. T. Elder, A. Franke, J. E. Gudjonsson, S. Weidinger, Atopic dermatitis is an IL-13–Dominant disease with greater molecular heterogeneity compared to psoriasis. *J. Invest. Dermatol.* **139**, 1480–1489 (2019).

31. H. Valdimarsson, J. E. Gudjonsson, A. Johnston, H. Sigmundsdottir, H. Valdimarsson, Immunopathogenic mechanisms in psoriasis. *Clin. Exp. Immunol.* **135**, 1–8 (2004).

32. L. Pasquali, A. Srivastava, F. Meisgen, K. Das Mahapatra, P. Xia, N. Xu Landén, A. Pivarcsi, E. Sonkoly, The keratinocyte transcriptome in psoriasis: Pathways related to immune responses, cell cycle and keratinization. *Acta Derm. Venereol.* **99**, 196–205 (2019).

33. H. Jin, M. K. Oyoshi, Y. Le, T. Bianchi, S. Koduru, C. B. Mathias, L. Kumar, S. Le Bras, D. Young, M. Collins, M. J. Grusby, J. Wenzel, T. Bieber, M. Boes, L. E. Silberstein, H. C. Oettgen, R. S. Geha, IL-21R is essential for epicutaneous sensitization and allergic skin inflammation in humans and mice. *J. Clin. Invest.* **119**, 47–60 (2009).

34. F. Gong, Q. Su, Y. H. Pan, X. Huang, W. H. Shen, The emerging role of interleukin-21 in allergic diseases (Review). *Biomed. Rep.* **1**, 837–839 (2013).

35. A. P. Sappino, I. Masouye, J. H. Saurat, G. Gabbiani, Smooth muscle differentiation in scleroderma fibroblastic cells. *Am. J. Pathol.* **137**, 585–591 (1990).

36. J. D'Orazio, S. Jarrett, A. Amaro-Ortiz, T. Scott, UV radiation and the skin. *Int. J. Mol. Sci.* **14**, 12222–12248 (2013).

37. M. Laporte, P. Galand, D. Fokan, C. De Graef, M. Heenen, Apoptosis in established and healing psoriasis. *Dermatology* **200**, 314–316 (2000).

38. A. Trautmann, M. Akdis, S. Klunker, K. Blaser, C. A. Akdis, Role of apoptosis in atopic dermatitis. *Int. Arch. Allergy Immunol.* **124**, 230–232 (2001).

39. B. Franz, B. Fritzsching, A. Riehl, N. Oberle, C. D. Klemke, J. Sykora, S. Quick, C. Stumpf, M. Hartmann, A. Enk, T. Ruzicka, P. H. Krammer, E. Suri-Payer, A. Kuhn, Low number of regulatory T cells in skin lesions of patients with cutaneous lupus erythematosus. *Arthritis Rheum.* **56**, 1910–1920 (2007).

40. R. Webb, J. T. Merrill, J. A. Kelly, A. Sestak, K. M. Kaufman, C. D. Langefeld, J. Ziegler, P. Robert, J. C. Edberg, R. Ramsey-goldman, M. Petri, J. D. Reveille, G. S. Alarcón, L. M. Vilá, M. E. Alarcón-Riquelme, J. A. James, G. S. Gilkeson, C. O. Jacob, K. L. Moser, P. M. Gaffney, T. J. Vyse, S. K. Nath, P. Lipsky, J. B. Harley, A. H. Sawalha, A polymorphism within interleukin-21 receptor (IL21R) confers risk for systemic lupus erythematosus. *Arthritis Rheumatol.* **60**, 2402–2407 (2009).

41. A. Pușcaș, A. Cătană, C. Pușcaș, I. Roman, C. Vornicescu, M. Șomlea, R. Orăsan, Psoriasis: Association of interleukin-17 gene polymorphisms with severity and response to treatment (Review). *Exp. Ther. Med.* 875–880 (2019).

42. A. C. Allison Billi, F. Ma, O. Plazyo, M. Gharaee-Kermani, R. Wasikowski, G. A. Hile, X. Xing, C. M. Yee, S. M. Rizvi, M. P. Maz, F. Wen, L. C. Tsoi, M. Pellegrini, R. L. Modlin, J. E. Gudjonsson, J. M. Kahlenberg, A. C. Billi, F. Ma, O. Plazyo, M. G.-Kermani, R. Wasikowski, G. A. Hile, X. Xing, C. M. Yee, S. M. Rizvi, M. P. Maz, F. Wen, L. C. Tsoi, M. Pellegrini, R. L. Modlin, J. E. Gudjonsson, J. M. Kahlenberg, Non-lesional and lesional lupus

skin share inflammatory phenotypes that drive activation of CD16+ dendritic cells. bioRxiv 2021.09.17.460124 [**Preprint**]. 20 September 2021. https://doi.org/10.1101/2021.09.17.460124.

43. E. Der, H. Suryawanshi, P. Morozov, M. Kustagi, B. Goilav, S. Ranabathou, P. Izmirly, R. Clancy, H. M. Belmont, M. Koenigsberg, M. Mokrzycki, H. Rominieki, J. A. Graham, J. P. Rocca, N. Bornkamp, N. Jordan, E. Schulte, M. Wu, J. Pullman, K. Slowikowski, S. Raychaudhuri, J. Guthridge, J. James, J. Buyon, T. Tuschl, C. Putterman, J. Anolik, W. Apruzzese, A. Arazi, C. Berthier, M. Brenner, J. Buyon, R. Clancy, S. Connery, M. Cunningham, M. Dall'Era, A. Davidson, E. Der, A. Fava, C. Fonseka, R. Furie, D. Goldman, R. Gupta, J. Guthridge, N. Hacohen, D. Hildeman, P. Hoover, R. Hsu, J. James, R. Kado, K. Kalunian, D. Kamen, M. Kretzler, H. Maecker, E. Massarotti, W. McCune, M. McMahon, M. Park, F. Payan-Schober, W. Pendergraft, M. Petri, M. Pichavant, C. Putterman, D. Rao, S. Raychaudhuri, K. Slowikowski, H. Suryawanshi, T. Tuschl, P. Utz, D. Waguespack, D. Wofsy, F. Zhang, Tubular cell and keratinocyte single-cell transcriptomics applied to lupus nephritis reveal type I IFN and fibrosis relevant pathways. *Nat. Immunol.* **20**, 915–927 (2019).

44. T. M. Li, K. R. Veiga, N. Schwartz, Y. Chinenov, D. J. Oliver, J. Lora, A. Jabbari, Y. Liu, W. D. Shipman, M. J. Sandoval, I. F. Sollohub, W. G. Ambler, M. Rashighi, J. G. Krueger, N. Anandasabapathy, C. P. Blobel, T. T. Lu, Type I interferon modulates Langerhans cell ADAM17 to promote photosensitivity in lupus. bioRxiv 2021.08.18.456792 [**Preprint**]. 18 August 2021. https://doi.org/10.1101/2021.08.18.456792.

45. K. A. Kirou, C. Lee, S. George, K. Louca, M. G. E. Peterson, M. K. Crow, Activation of the interferon-α pathway identifies a subgroup of systemic lupus erythematosus patients with distinct serologic features and active disease. *Arthritis Rheum.* **52**, 1491–1503 (2005).

46. Q. Z. Li, J. Zhou, Y. Lian, B. Zhang, V. K. Branch, F. Carr-Johnson, D. R. Karp, C. Mohan, E. K. Wakeland, N. J. Olsen, Interferon signature gene expression is correlated with autoantibody profiles in patients with incomplete lupus syndromes. *Clin. Exp. Immunol.* **159**, 281–291 (2010).

47. E. L. Hubbard, D. S. Pisetsky, P. E. Lipsky, Anti-RNP antibodies are associated with the interferon gene signature but not decreased complement levels in SLE. *Ann. Rheum. Dis.* (2022).

48. C. C. Berthier, L. C. Tsoi, T. J. Reed, J. N. Stannard, E. M. Myers, R. Namas, X. Xing, S. Lazar, L. Lowe, M. Kretzler, J. E. Gudjonsson, J. M. Kahlenberg, Molecular profiling of cutaneous lupus lesions identifies subgroups distinct from clinical phenotypes. *J. Clin. Med.* **8**, 1244 (2019).

49. S. Zampieri, M. Alaibac, L. Iaccarino, R. Rondinone, A. Ghirardello, P. Sarzi-Puttini, A. Peserico, A. Doria, Tumour necrosis factor α is expressed in refractory skin lesions from patients with subacute cutaneous lupus erythematosus. *Ann. Rheum. Dis.* **65**, 545–548 (2006).

50. L. E. Tomalin, C. B. Russell, S. Garcet, D. A. Ewald, P. Klekotka, A. Nirula, H. Norsgaard, M. Suàrez-Fariñas, J. G. Krueger, Short-term transcriptional response to IL-17 receptor-A antagonism in the treatment of psoriasis. *J. Allergy Clin. Immunol.* **145**, 922–932 (2020).

51. M. Robert, P. Miossec, Interleukin-17 and lupus: Enough to be a target? For which patients?. *Lupus* **29**, 6–14 (2020).

52. A study to assess the safety and efficacy of secukinumab in alleviating symptoms of discoid lupus erythematosus. *U.S. Natl. Libr. Med. Clin. Trials* (2021).

53. B. Ungar, A. B. Pavel, R. Li, G. Kimmel, J. Nia, P. Hashim, H. J. Kim, M. Chima, A. S. Vekaria, Y. Estrada, H. Xu, X. Peng, G. K. Singer, D. Baum, Y. Mansouri, M. Taliercio, E. Guttman-Yassky, Phase 2 randomized, double-blind study of IL-17 targeting with secukinumab in atopic dermatitis. *J. Allergy Clin. Immunol.* **147**, 394–397 (2021).

54. S. Tyring, A. Gottlieb, K. Papp, K. Gordon, C. Leonardi, A. Wang, D. Lalla, M. Woolley, A. Jahreis, R. Zitnik, D. Cella, R. Krishnan, Etanercept and clinical outcomes, fatigue, and depression in psoriasis: Double-blind placebo-controlled randomised phase III trial. *Lancet* **367**, 29–35 (2006).

55. K. Reich, F. O. Nestle, K. Papp, J. P. Ortonne, R. Evans, C. Guzzo, S. Li, L. T. Dooley, C. E. M. Griffiths, Infliximab induction and maintenance therapy for moderate-to-severe psoriasis: A phase III, multicentre, double-blind trial. *Lancet* **366**, 1367–1374 (2005).

56. A. Menter, S. K. Tyring, K. Gordon, A. B. Kimball, C. L. Leonardi, R. G. Langley, B. E. Strober, M. Kaul, Y. Gu, M. Okun, K. Papp, Adalimumab therapy for moderate to severe psoriasis: A randomized, controlled phase III trial. *J. Am. Acad. Dermatol.* **58**, 106–115 (2008).

57. A. Blauvelt, K. Reich, M. Lebwohl, D. Burge, C. Arendt, L. Peterson, J. Drew, R. Rolleri, A. B. Gottlieb, Certolizumab pegol for the treatment of patients with moderate-to-severe chronic plaque psoriasis: Pooled analysis of week 16 data from three randomized controlled trials. *J. Eur. Acad. Dermatol. Venereol.* **33**, 546–552 (2019).

58. A. Lorenzo-Vizcaya, D. A. Isenberg, The use of anti-TNF-alpha therapies for patients with systemic lupus erythematosus. Where are we now?. *Expert Opin. Biol. Ther.* **21**, 639–647 (2021).

59. A. Jacobi, C. Antoni, B. Manger, G. Schuler, M. Hertl, Infliximab in the treatment of moderate to severe atopic dermatitis. *J. Am. Acad. Dermatol.* **52**, 522–526 (2005).

60. N. Cassano, F. Loconsole, C. Coviello, Infliximab in recalcitrant severe atopic eczema associated with contact allergy. *Int. J. Immunopathol. Pharmacol.* **19**, 237–240 (2006).

61. M. Yuzaiful, M. Yusof, M. Wittmann, C. Fernandez, D. Wilson, S. Edward, G. Abignano, A. Alase, P. Laws, M. Goodfield, P. Emery, E. Vita, Targeted therapy using intradermal injection of etanercept for remission induction in discoid lupus erythematosus (TARGET-DLE): Results from a proof-of-concept phase II trial. *Lupus Sci. Med.* **6**, A1–A227 (2019).

62. Targeted therapy using intradermal injection of etanercept for remission induction in discoid lupus erythematosus (TARGET-DLE). *U.S. Natl. Libr. Med. Clin. Trials* (2019).

63. A. B. Gottlieb, A. M. Goldminz, Ustekinumab for psoriasis and psoriatic arthritis. *J. Rheumatol.* **39**, 86–89 (2012).

64. A study of ustekinumab in participants with active systemic lupus erythematosus (2021); https://clinicaltrials.gov/ct2/show/NCT03517722?term=ustekinumab&cond=lupus&draw=2&rank=3.

65. Janssen Pharmaceuticals, Janssen announces discontinuation of phase 3 LOTUS study evaluating ustekinumab in systemic lupus erythematosus (2020); www.jnj.com/janssen-announces-discontinuation-of-phase-3-lotus-study-evaluating-ustekinumab-in-systemic-lupus-erythematosus.

66. R. F. van Vollenhoven, B. H. Hahn, G. C. Tsokos, C. L. Wagner, P. Lipsky, Z. Touma, V. P. Werth, R. M. Gordon, B. Zhou, B. Hsu, M. Chevrier, M. Triebel, J. L. Jordan, S. Rose, Efficacy and safety of ustekinumab, an IL-12 and IL-23 inhibitor, in patients with active systemic lupus erythematosus: Results of a multicentre, double-blind, phase 2, randomised, controlled study. *Lancet* **392**, 1330–1339 (2018).

67. W. D. Shipman, S. Chyou, A. Ramanathan, P. M. Izmirly, S. Sharma, T. Pannellini, D. C. Dasoveanu, X. Qing, C. M. Magro, R. D. Granstein, M. A. Lowes, E. G. Pamer, D. H. Kaplan, J. E. Salmon, B. J. Mehrara, J. W. Young, R. M. Clancy, C. P. Blobel, T. T. Lu, A protective Langerhans cell keratinocyte axis that is dysfunctional in photosensitivity. *Sci. Transl. Med.* **10**, eaap9527 (2018).

68. S. Tian, J. G. Krueger, K. Li, A. Jabbari, C. Brodmerkel, M. A. Lowes, M. Suárez-Fariñas, Meta-analysis derived (MAD) transcriptome of psoriasis defines the “Core” pathogenesis of disease. *PLOS ONE* **7**, e44274 (2012).

69. L. Möbus, E. Rodriguez, I. Harder, D. Stölzl, N. Boraczynski, S. Gerdes, A. Kleinheinz, S. Abraham, A. Heratizadeh, C. Handrick, E. Haufe, T. Werfel, J. Schmitt, S. Weidinger, Atopic dermatitis displays stable and dynamic skin transcriptome signatures. *J. Allergy Clin. Immunol.* **147**, 213–223 (2021).

70. L. C. Tsoi, M. T. Patrick, S. Shuai, M. K. Sarkar, S. Chi, B. Ruffino, A. C. Billi, X. Xing, R. Uppala, C. Zang, J. Fullmer, Z. He, E. Maverakis, N. N. Mehta, B. E. Perez White, S. Getsios, Y. Helfrich, J. J. Voorhees, J. M. Kahlenberg, S. Weidinger, J. E. Gudjonsson,

Cytokine responses in nonlesional psoriatic skin as clinical predictor to anti-TNF agents. *J. Allergy Clin. Immunol.* **75**, 15–18 (2021).

71. E. Eisenberg, E. Y. Levanon, Human housekeeping genes, revisited. *Trends Genet.* **29**, 569–574 (2013).

72. S. Hänzelmann, R. Castelo, J. Guinney, GSVA: Gene set variation analysis for microarray and RNA-Seq data (2013); www.biomedcentral.com/1471-2105/14/7http://www.bioconductor.org.Background.

73. C. Cheadle, M. P. Vawter, W. J. Freed, K. G. Becker, Analysis of microarray data using z score transformation.*J. Mol. Diagn.* **5**, 73–81.

74. J. Menche, E. Guney, A. Sharma, P. J. Branigan, M. J. Loza, F. Baribaud, R. Dobrin, A. L. Barabási, Integrating personalized gene expression profiles into predictive disease-associated gene pools. *npj Syst. Biol. Appl.* **3**, 10 (2017).

75. M. Uhlén, L. Fagerberg, B. M. Hallström, C. Lindskog, P. Oksvold, A. Mardinoglu, Å. Sivertsson, C. Kampf, E. Sjöstedt, A. Asplund, I. M. Olsson, K. Edlund, E. Lundberg, S. Navani, C. A. K. Szigyarto, J. Odeberg, D. Djureinovic, J. O. Takanen, S. Hober, T. Alm, P. H. Edqvist, H. Berling, H. Tegel, J. Mulder, J. Rockberg, P. Nilsson, J. M. Schwenk, M. Hamsten, K. Von Feilitzen, M. Forsberg, L. Persson, F. Johansson, M. Zwahlen, G. Von Heijne, J. Nielsen, F. Pontén, Tissue-based map of the human proteome. *Science* **347**, 394 (2015).

76. A. Gazel, P. Ramphal, M. Rosdy, B. De Wever, C. Tornier, N. Hosein, B. Lee, M. Tomic-Canic, M. Blumenberg, Transcriptional profiling of epidermal keratinocytes: Comparison of genes expressed in skin, cultured keratinocytes, and reconstituted epidermis, using large DNA microarrays. *J. Invest. Dermatol.* **121**, 1459–1468 (2003).

77. L. Breiman, J. H. Friedman, R. A. Olshen, C. J. Stone, *Classification and Regression Trees* (1984).

78. L. Breiman, Random forests. *Mach. Learn.* **45**, 5–32 (2001).

79. R. Blagus, L. Lusa, SMOTE for high-dimensional class-imbalanced data. *BMC Bioinformatics* **14**, 106 (2013).

80. Z. Gu, R. Eils, M. Schlesner, Complex heatmaps reveal patterns and correlations in multidimensional genomic data. *Bioinformatics* **32**, 2847–2849 (2016).
